# Atrial arrhythmogenicity of *KCNJ2* mutations in short QT syndrome: Insights from virtual human atria

**DOI:** 10.1371/journal.pcbi.1005593

**Published:** 2017-06-13

**Authors:** Dominic G. Whittaker, Haibo Ni, Aziza El Harchi, Jules C. Hancox, Henggui Zhang

**Affiliations:** 1 Biological Physics Group, School of Physics & Astronomy, The University of Manchester, Manchester, United Kingdom; 2 Department of Physiology, Pharmacology and Neuroscience, and Cardiovascular Research Laboratories, School of Medical Sciences, University of Bristol, Bristol, United Kingdom; 3 School of Computer Science and Technology, Harbin Institute of Technology, Harbin, China; 4 Space Institute of Southern China, Shenzhen, China; Universiteit Gent, BELGIUM

## Abstract

Gain-of-function mutations in *KCNJ2-*encoded Kir2.1 channels underlie variant 3 (SQT3) of the short QT syndrome, which is associated with atrial fibrillation (AF). Using biophysically-detailed human atria computer models, this study investigated the mechanistic link between SQT3 mutations and atrial arrhythmogenesis, and potential ion channel targets for treatment of SQT3. A contemporary model of the human atrial action potential (AP) was modified to recapitulate functional changes in I_K1_ due to heterozygous and homozygous forms of the D172N and E299V Kir2.1 mutations. Wild-type (WT) and mutant formulations were incorporated into multi-scale homogeneous and heterogeneous tissue models. Effects of mutations on AP duration (APD), conduction velocity (CV), effective refractory period (ERP), tissue excitation threshold and their rate-dependence, as well as the wavelength of re-entry (WL) were quantified. The D172N and E299V Kir2.1 mutations produced distinct effects on I_K1_ and APD shortening. Both mutations decreased WL for re-entry through a reduction in ERP and CV. Stability of re-entrant excitation waves in 2D and 3D tissue models was mediated by changes to tissue excitability and dispersion of APD in mutation conditions. Combined block of I_K1_ and I_Kr_ was effective in terminating re-entry associated with heterozygous D172N conditions, whereas I_Kr_ block alone may be a safer alternative for the E299V mutation. Combined inhibition of I_Kr_ and I_Kur_ produced a synergistic anti-arrhythmic effect in both forms of SQT3. In conclusion, this study provides mechanistic insights into atrial proarrhythmia with SQT3 Kir2.1 mutations and highlights possible pharmacological strategies for management of SQT3-linked AF.

## Author summary

Atrial fibrillation (AF) is the most common cardiac arrhythmia, and is characterised by complex and irregular electrical activation of the upper chambers of the heart. One rare, genetic condition associated with increased risk of AF is the short QT syndrome (SQTS), which is caused by mutations in genes involved in normal electrical function of the heart. Underlying mechanisms by which SQTS-related gene mutations facilitate development of arrhythmias in the human atria are not well understood. In this study, sophisticated computer models representing ‘virtual’ human atria, incorporating detailed electrophysiological data at the ‘ion channel’ protein level into both idealised and realistic multi-scale tissue geometries, were used to dissect mechanisms by which two mutations in the *KCNJ2* gene responsible for SQTS variant 3 (SQT3) promote initiation and sustenance of arrhythmias. It was found that the D172N and E299V mutations to *KCNJ2* accelerated the repolarisation process at the cellular level through distinct mechanisms. This, along with the way the mutations affected heterogeneity in electrical behaviour at the organ level, mediated stability of arrhythmias and response to simulated ion channel block. This study improves understanding of mechanisms underlying increased AF risk associated with D172N and E299V *KCNJ2* mutations, and outlines potential therapeutic strategies.

## Introduction

The cardiac inward rectifier potassium current (I_K1_) is responsible for stabilising the resting membrane potential (RMP) and contributes to terminal repolarisation of both atrial and ventricular action potentials (APs) [1]. The K^+^ channels that mediate I_K1_ are comprised of Kir2.x family of subunits, of which the *KCNJ2*-encoded Kir2.1 is strongly expressed in both the atria and ventricles [2]. Loss-of-function of Kir2.1 channels has been implicated in the Andersen-Tawil syndrome (long QT syndrome type 7) and causes ventricular arrhythmias and a range of extracardiac abnormalities [3]. Gain-of-function *KCNJ2* mutations are also potentially life-threatening, underlying variant 3 of the short QT syndrome (SQT3) [4–6], as well as familial atrial fibrillation (AF) [7].

The short QT syndrome (SQTS) is a genetic disorder that has been associated with increased risk of ventricular *and* atrial arrhythmias, and of sudden cardiac death [8,9]. The SQTS phenotype is characterised by an abbreviated QT interval, tall and peaked T waves, and loss of rate-adaptation of the QT interval [9]. To date, four missense mutations have been identified in *KCNJ2*-linked short QT syndrome (SQT3): D172N [4], M301K [5], E299V [6], and K346T [10], with reports of AF in some patients [5,6]. The first reported SQT3 mutation, D172N Kir2.1 [4], was shown to increase significantly outward I_K1_ at potentials between −75 mV and −45 mV. The proband and her father exhibited significantly shortened QT_c_ intervals (315 ms and 320 ms, respectively) and presented with a history of presyncopal events and palpitations [4]. The subsequently discovered E299V Kir2.1 mutation was shown to differ from D172N in that it strongly affects inward rectification, resulting in large outward I_K1_ currents at potentials above −55mV [6]. The proband presented with an extremely short QT interval (200 ms) which showed no rate-adaptation, and recurring episodes of paroxysmal AF [6].

A link between increased I_K1_ and *acquired* AF is well-established, with upregulation of I_K1_ consistently observed in chronic AF-induced electrical remodelling of the human atrial action potential (AP) [11–14]. Previously it has been shown that increased I_K1_ shortened the action potential duration (APD) and stabilised rotors in computational models of human atrial electrophysiology [15–18]. Increased I_K1_ arising from the E299V Kir2.1 mutation has also been shown to be pro-arrhythmic at cellular and 2D tissue levels [6]. However, the atria are characterised by marked electrical heterogeneity [19–22], which has been shown previously to generate an arrhythmia substrate *in silico* [23,24]. Mechanisms underlying initiation and maintenance of atrial arrhythmias at cellular and organ levels arising from interactions between genetic mutations and intrinsic electrical heterogeneities in human atria have yet to be investigated using anatomically-detailed, 3D phenotypically-accurate models of SQT3 mutant I_K1_.

Furthermore, possible similarities and/or differences in the atrial pro-arrhythmic mechanisms of Kir2.1 mutations due to their distinct effects on I_K1_ in the SQTS have not yet been characterised. The D172N and E299V mutations have distinct effects on currents carried by Kir2.1 channels; the D172 residue is involved in the steep, highly voltage-dependent portion of rectification [4,25,26], whereas E299 is involved in the shallow part of rectification which shows less voltage dependence [6,27]. The effects of the E299V mutation on Kir2.1 currents are more pervasive than are those of Kir2.1 D172N, as the E299V mutation significantly alters the membrane potential range over which I_K1_ is active, fundamentally changing its role in repolarisation of the AP. The comparative pro-arrhythmic effects of these two distinct mutations have yet to be elucidated.

This study aimed to evaluate and compare the influence of the SQT3 D172N and E299V Kir2.1 mutations on atrial repolarisation and susceptibility to re-entrant excitation, using heterogeneous, multi-scale models of human atrial electrophysiology. It also aimed to investigate the effects of simulated ion channel inhibition on SQT3-mediated AF, as this may provide theoretical insights into possible pharmacological approaches for the management of AF in SQT3.

## Results

### Model validation

**Fig 1:**
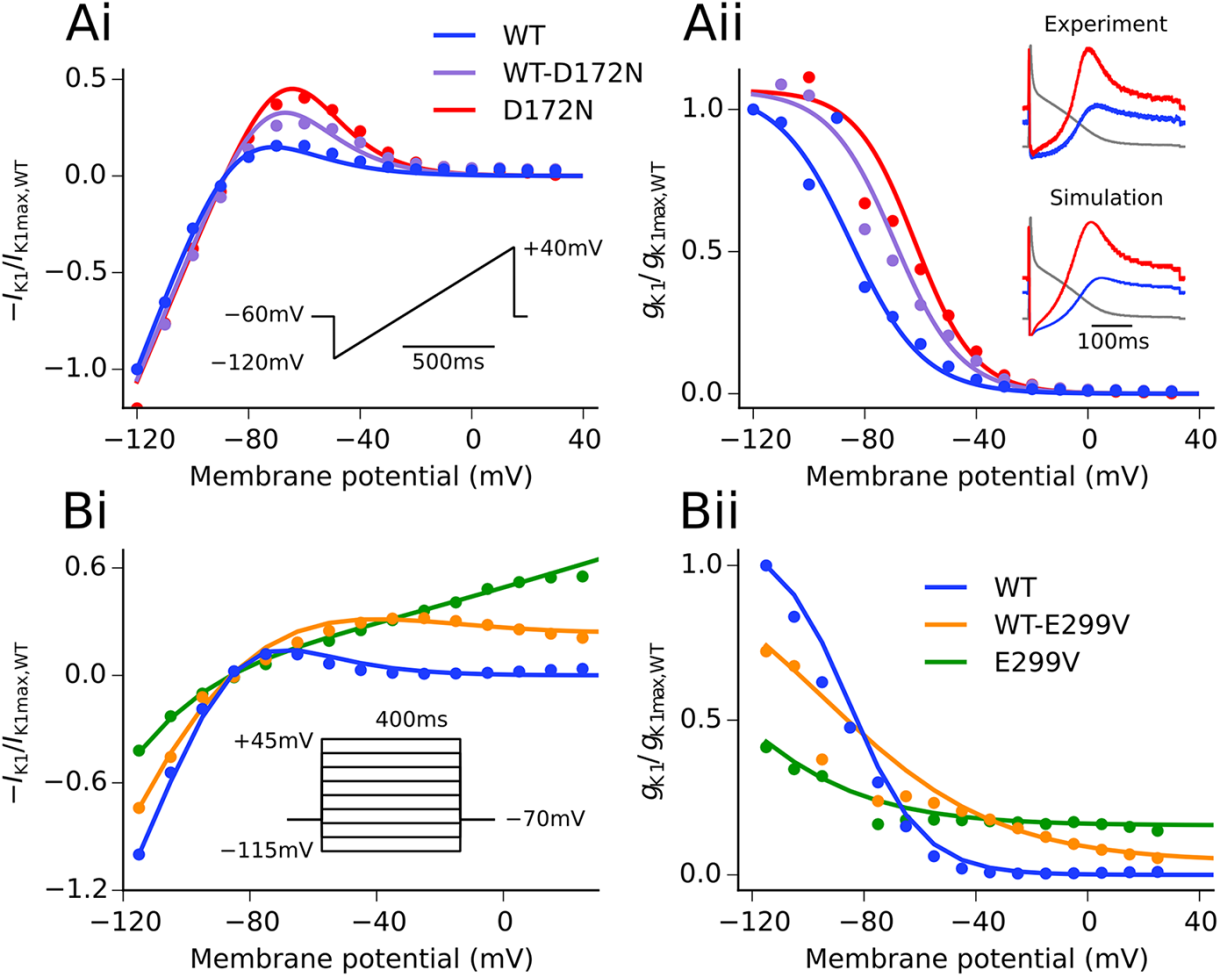
**Experimental and simulated I-V and *g***_**K1**_**-V relationships.** Comparison of model I-V relationship (Ai) and computed *g*_K1_-V relationship (Aii) in WT, WT-D172N, and D172N conditions (solid lines) with the experimental data of El Harchi *et al*. [26] (points). Simulated AP clamps using a digitised AP waveform from the Nygren *et al*. human atrial cell model [30] as described in [26] are shown inset. Comparison of model I-V relationship (Bi) and computed *g*_K1_-V relationship (Bii) in WT, WT-E299V, and E299V conditions (solid lines) with the experimental data of Deo *et al*. [6] (points). The voltage clamp protocol used in each case is shown inset in (i).

**Fig 2:**
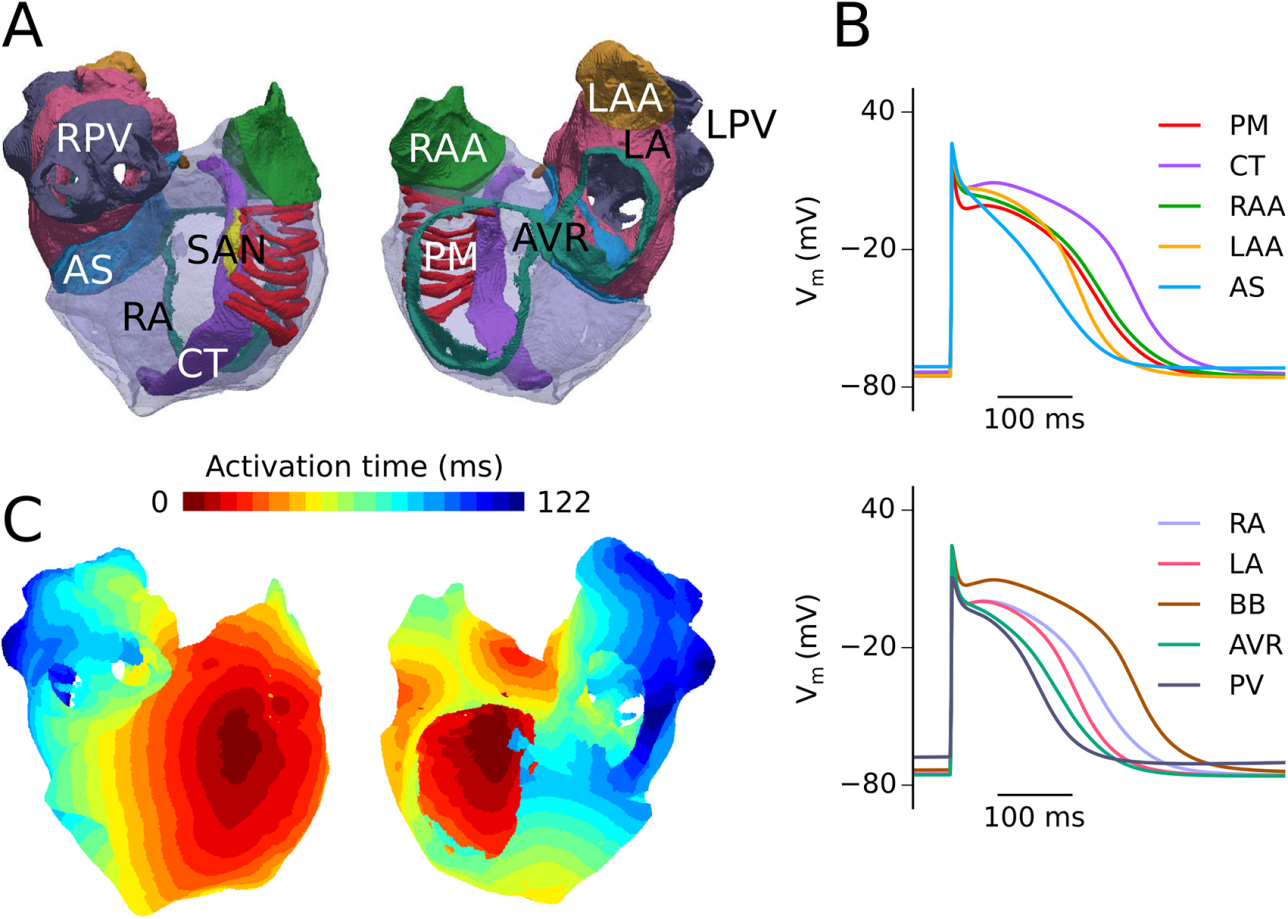
3D anatomical atria model and regional APs. The anatomical atria geometry shown from two views–looking at the RA posterior wall and into the cavities (A), with regions of atrial septum (AS), atrio-ventricular ring (AVR), crista terminalis (CT), left atrium (LA), left atrial appendage (LAA), left pulmonary veins (LPV), pectinate muscles (PM), right atrium (RA), right atrial appendage (RAA), right pulmonary veins (RPV), and sinoatrial node (SAN) shown for reference. Action potentials for regional cell models including the Bachmann’s bundle (BB) at 1 Hz (B), with colours corresponding to tissue segmentation in (A). Activation sequence in the heterogeneous 3D anatomical atria model, following initiation of an AP in the SAN region (C).

I_K1_ formulations developed in wild-type (WT) and SQT3 mutant conditions accurately reproduced experimental I-V relationship data under voltage clamp conditions; namely (i) significantly increased outward currents at potentials in the range of −75 to −45 mV in D172N mutation conditions, and (ii) impaired inward rectification and increased outward I_K1_ at potentials above −55 mV associated with the E299V mutation, as shown in Fig 1. Furthermore, simulated AP clamps using the same digitised human atrial AP waveform as employed in previous *in vitro* experiments showed strong agreement with those obtained experimentally in WT and D172N conditions [26]. The Colman *et al*. (CZ) human atrial cell model updated with our WT I_K1_ formulation gave AP characteristics which were in excellent agreement with experimental data from human atrial myocytes (Supplemental S1 and S2 Figs). Incorporation of the modified family of CZ regional cell models into our 3D human atria geometry, as shown in Fig 2, gave a total activation time (AT) and conduction velocity (CV) in good agreement with experimental measurements (total AT 122 ms versus 116 ± 18 ms [28]; CV 0.71 ms^−1^ in the RA versus 0.7–0.9 ms^−1^ in RA free wall [29]).

### Effects of SQT3 mutant I_K1_ on single cell atrial APs

**Fig 3:**
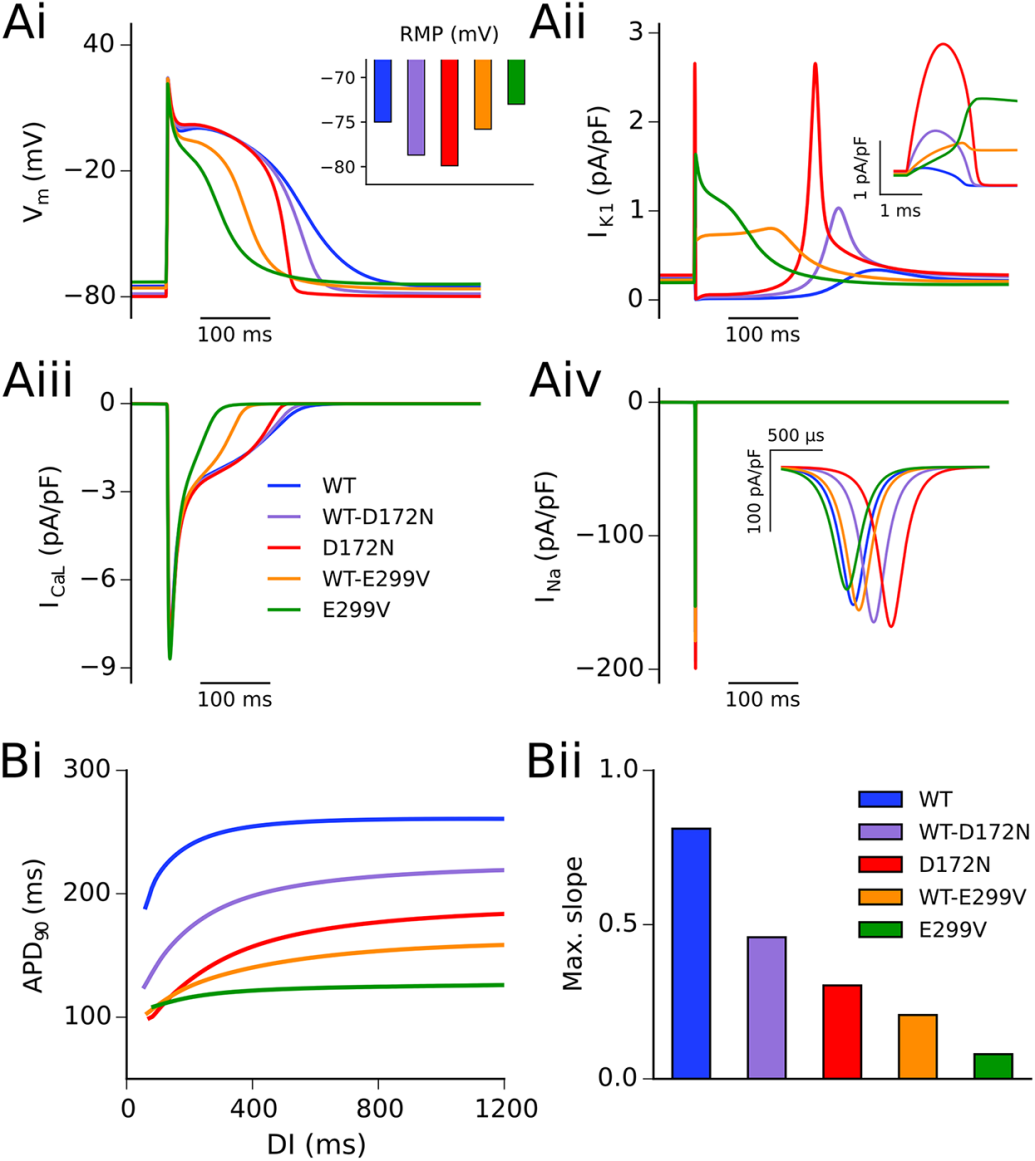
Simulated AP profiles and current traces in WT and mutation conditions. Action potential profiles in WT (blue), WT-D172N (mauve), D172N (red), WT-E299V (orange), and E299V (green) conditions at a pacing frequency of 1 Hz (Ai), with inset showing RMP. Corresponding current traces are shown for I_K1_ (Aii), I_CaL_ (Aiii), and I_Na_ (Aiv). Insets for panels (Aii) and (Aiv) show current profiles during the AP upstroke for I_K1_ and I_Na_, respectively. (Bi) Restitution of the APD_90_ as determined using an S1-S2 protocol, and (Bii) maximal slope of restitution.

Alterations to I_K1_ due to SQT3 mutations accelerated the repolarisation phase of action potentials in all conditions investigated, as shown in Fig 3A. The D172N mutation resulted in shortening of APD_90_ and hyperpolarisation of the resting membrane potential (RMP) (Fig 3Ai), with the homozygous condition exerting a more profound effect than the heterozygous condition. This was associated with an increased outward I_K1_ without altered rectification (see Fig 1A), providing a stronger repolarising current during terminal repolarisation (Fig 3Aii). In contrast, as the E299V mutation markedly altered (WT-E299V) or abolished (E299V) inward rectification of I_K1_ over the physiological range of membrane potentials (see Fig 1B), it resulted in a significant contribution of outward I_K1_ during the entire AP, suppressing the AP plateau and uniformly shortening the APD (Fig 3Ai and 3Aii). A summary of the effects of the studied mutations on AP characteristics at a pacing frequency of 1 Hz is shown in Table 1.

**Table 1:** AP properties in WT and SQT3 mutant conditions in the baseline single cell model.

	APA (mV)	RMP (mV)	APD_50_ (ms)	APD_90_ (ms)	MUV (V/s)
**WT**	99.2	-75.0	171.6	259.9	194.5
**WT-D172N**	103.0	-78.6	166.6	213.9	214.4
**D172N**	103.5	-79.9	154.7	177.5	200.5
**WT-E299V**	99.8	-75.8	99.4	154.4	199.4
**E299V**	94.5	-73.0	60.4	125.0	173.1

A summary of atrial AP characteristics: action potential amplitude (APA), resting membrane potential (RMP), action potential duration at 50% and 90% repolarisation (APD_50_ and APD_90_, respectively), and maximum upstroke velocity (MUV) in the baseline single cell model at a pacing frequency of 1 Hz.

Fig 3 also shows secondary effects of mutant I_K1_ on I_CaL_ (Fig 3Aiii) and I_Na_ (Fig 3Aiv). The D172N mutation conditions affected the AP during terminal repolarisation; at this phase of the AP little inward current is passed through the L-type calcium channel, thus the profile of I_CaL_ was not significantly affected. The E299V mutation conditions, which reduced or abolished inward rectification of I_K1_ over physiological voltages, caused a decreased plateau potential of the AP, reducing I_CaL_ during this phase. The effect of mutations on the RMP altered the profile of I_Na_ in two ways; (i) hyperpolarised RMP as seen in the D172N conditions increased sodium current availability thus increasing peak current density, and (ii) time for activation of I_Na_ following stimulation increased as a function of the voltage difference between the RMP and threshold potential for AP initiation (or decreased in the case of the homozygous E299V mutation which was associated with more depolarised RMP). Whereas the WT-E299V mutation did not significantly affect the RMP, the homozygous E299V mutation condition led to a small depolarisation of the RMP, due to decreased outward currents in the region of ~70–80 mV. Elevation of the RMP, as seen for the E299V mutation, resulted in partial inactivation of sodium channels, decreasing I_Na_ amplitude (Fig 3Aiv), the action potential amplitude (APA) and maximum upstroke velocity (MUV) (see Table 1).

All mutation conditions investigated shortened the APD_90_ across a wide range of diastolic intervals (DIs) (Fig 3Bi). The maximal slope of APD restitution (Fig 3Bii) was decreased in all SQT3 mutation conditions compared to the WT, with the homozygous E299V mutation in particular showing almost no rate adaptation. Whereas the APD measured in the homozygous D172N condition was longer than that in both heterozygous and homozygous E299V mutations at normal pacing rates, at fast pacing rates the homozygous D172N restitution curve crossed over the E299V curves.

### Effects of SQT3 mutant I_K1_ on tissue APD distribution

**Fig 4:**
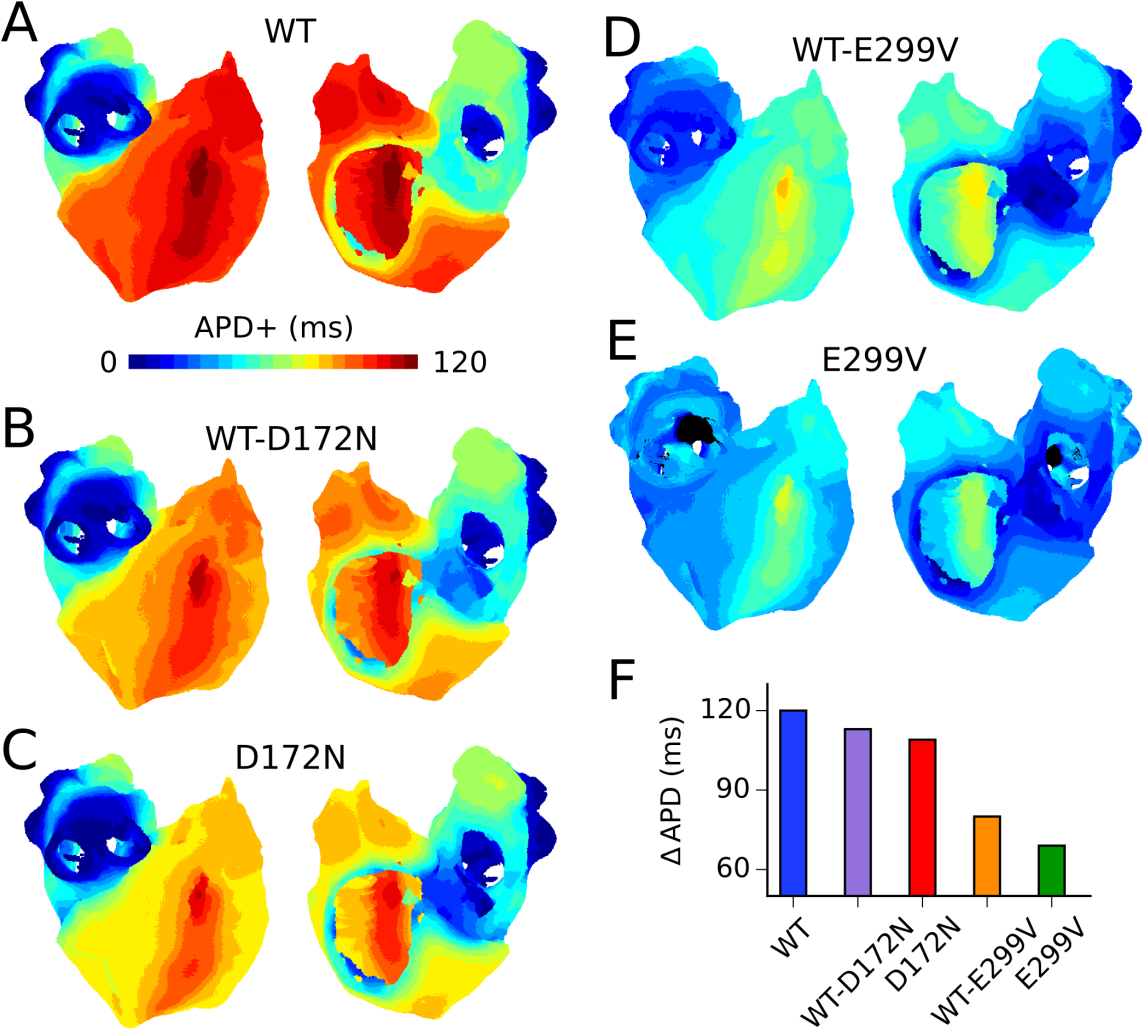
Spatial dispersion of APD in 3D anatomical atria model. APD distribution maps in WT (A), WT-D172N (B), D172N (C), WT-E299V (D), and E299V (E) mutation conditions, with corresponding global ΔAPD (F). The colour bar shows APD relative to the shortest APD measured in each condition, designated APD+. The scale of the colour bar is fixed at the value of ΔAPD in the WT condition (120 ms) to facilitate comparison between the mutation conditions. The colour black shows regions where membrane potentials failed to exceed a threshold value (−20 mV).

Each regional cell model incorporated into the 3D virtual human atria has a different AP profile due to intrinsic different electrophysiological properties of the atria [19,22,31]. The spatial distribution of the action potential duration, ΔAPD, in coupled tissue is influenced by electrotonic coupling, with regional APD depending on region size and the APD of neighbouring regions. ΔAPD across the 3D human atria geometry at a pacing frequency of 1 Hz for all mutations is shown in Fig 4. The D172N mutation conditions showed only a modest decrease in global ΔAPD, whilst increasing APD heterogeneity at the CT/PM junction and preserving a high degree of heterogeneity at the PV/LA junction (summarised in S3 Fig). The E299V mutation conditions, however, caused a more marked decrease in ΔAPD, with the homozygous E299V mutant showing the most homogenous APD distribution (overall ΔAPD of 69 ms versus 120 ms in the WT case). Whereas ΔAPD at the CT/PM junction was increased for the E299V mutation conditions, there was almost no difference in APD at the PV/LA junction for the WT-E299V condition, and APs failed to propagate uniformly across the PV region in the E299V condition (conduction blocks shown in black). This was due to the combination of reduced CV and increased electrotonic suppression arising from differences in RMP from the surrounding LA region. Augmented regional differences at the CT/PM junction led to an increase in the temporal vulnerability to uni-directional conduction block in all mutation conditions (see S4 Fig).

### Effects of SQT3 mutant I_K1_ on tissue restitution properties

**Fig 5:**
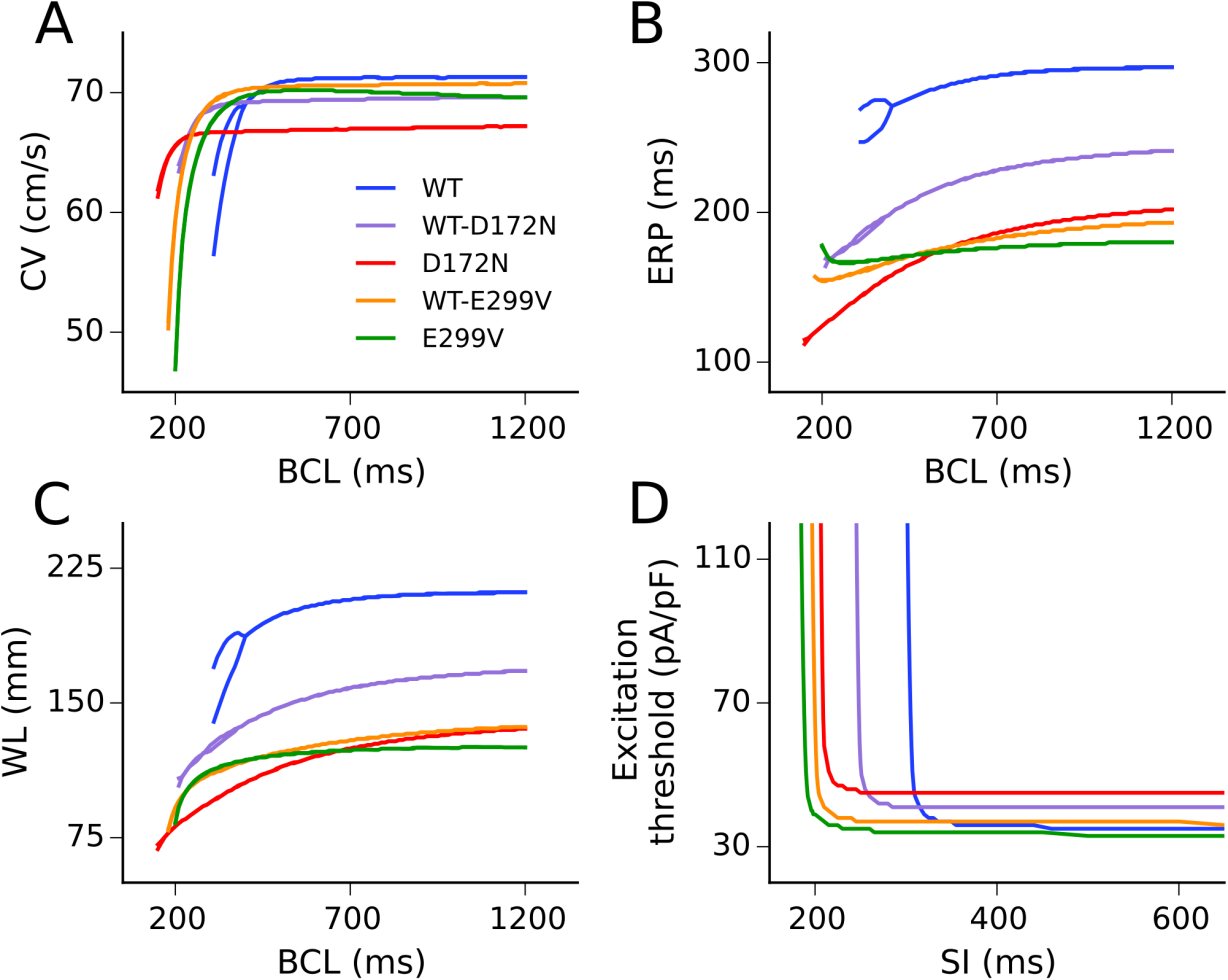
Tissue restitution properties in WT and mutation conditions. Restitution of the CV (A), ERP (B), and WL of re-entry (C) against a range of basic cycle lengths (BCL), and excitation threshold (D) against a range of S1-S2 stimulus intervals (SI), as measured in the 1D tissue model.

Restitution curves for the conduction velocity (CV), effective refractory period (ERP), wavelength (WL) of re-entry, and tissue excitability measured in the 1D strand are shown in Fig 5. Alterations to CV restitution (Fig 5A) were mediated primarily by effects of the mutations on the RMP. The D172N mutation conditions hyperpolarised the RMP; this resulted in increased sodium channel availability, outweighing any antagonistic effect of increased outward I_K1_ during membrane depolarisation and increasing single cell MUV (see Table 1) which might be expected to lead to an increase in CV. However, the increased voltage gradient between active and resting cells in tissue resulted in higher electrotonic suppression at the wavefront, causing an overall decrease in conduction velocity. The CV in the WT-E299V condition was decreased by similar mechanisms. For the homozygous E299V mutation condition, the voltage gradient between active and resting cells was reduced, yet the combination of depolarised RMP (which decreases sodium channel availability) and increased outward currents during membrane depolarisation led to a more significant reduction in CV than for the heterozygous mutant form.

The ERP was shortened in all mutation conditions compared to the WT, with the homozygous E299V condition showing the least rate adaptation (Fig 5B). ERP restitution in the E299V and WT-E299V mutation conditions showed a deflection to higher values at fast rates, with minima at basic cycle lengths (BCL) of ~270 ms and ~200 ms, respectively. As with the single cell APD restitution, the D172N ERP restitution curve crossed those of the E299V mutation conditions at fast rates. The WL of re-entry was shortened for all SQT3 mutants across the range of DIs investigated (Fig 5C). AP alternans arising from fast rate pacing were reduced in all mutation conditions, consistent with a decrease in the steepness of restitution.

The rate-dependent effect of the SQT3 mutations on tissue excitability, measured as excitation threshold, is shown in Fig 5D. At low rates the D172N mutation conditions decreased tissue excitability, the WT-E299V mutation did not significantly affect excitability, and the homozygous E299V mutation increased excitability. At high rates, all mutations investigated increased excitability compared to the WT. For example, at a stimulus interval (SI) of 300 ms the excitation threshold for the WT tissue was 169 pA/pF compared with 41 pA/pF for WT-D172N, 45 pA/pF for D172N, 37 pA/pF for WT-E299V, and 34 pA/pF for E299V mutant tissue. Differences in excitation threshold between mutation conditions can be explained by how the mutations affected the RMP, i.e. mutations which hyperpolarised the RMP increased the voltage gradient between the RMP and the threshold for AP firing, thus larger stimulus currents were required to elicit APs which propagate in tissue. The SI at which the excitation threshold began to tend towards an infinite value is determined by the degree of APD shortening in each of the mutation conditions.

### Characterisation of spiral wave dynamics in 2D due to SQT3 mutant I_K1_

Further simulations were performed using an idealised area of 2D tissue to investigate the functional impacts of the SQT3 mutations on the dynamic behaviours of re-entrant excitation waves, including the lifespan, stability and meandering pattern of the re-entry (see S5 and S6 Figs). In the WT condition, re-entrant waves had an average lifespan of <300 ms, as the tip of the re-entry consistently meandered out of the tissue boundaries. As for the WT-D172N mutation condition, re-entry sustained (lasted for the 5.0 s duration of the simulation) in 3/5 simulations, with spiral waves drifting unpredictably and following relatively linear trajectories (see S5 Fig) [32]. In the homozygous D172N mutation condition, sustained re-entry occurred in 5/5 simulations. When the S2 stimulus was applied early after the ERP, the emergent spiral wave interacted with the refractory tail causing transient wave break and meander, before settling into a stationary, tightly-clustered hyper-meandering trajectory [32].

In the WT-E299V condition, re-entry sustained in 5/5 simulations. However, this mutation condition was the most prone to wave break, due to drift-induced differences in refractoriness across the 2D tissue patch and a high frequency of rotation. The homozygous E299V mutation produced sustained re-entry in 3/5 simulations. This mutation produced spiral waves which rotated with a slower dominant frequency (DF) than the heterozygous form of the mutation (see S3 Table). Spiral wave trajectories in the E299V condition drifted significantly and showed distinctive ‘outward petals’, also known as a hypocycloidal meander pattern [32].

The temporal evolution of spiral wave core trajectories in 2D tissue is shown in S5 Fig for all mutation conditions and the average lifespan of spiral waves and area of meander in time are summarised in S6 Fig. Whereas both the D172N and WT-E299V mutation conditions sustained in all simulations, spiral waves in the WT-E299V condition meandered over a much larger area on average (representative simulations shown in S1–S5 Videos). The average lifespan of spiral waves in the homozygous E299V mutation condition was shorter than in the heterozygous condition due to increased tissue excitability, which led to a more unstable form of re-entry.

### Arrhythmia simulations in the 3D virtual human atria

**Fig 6:**
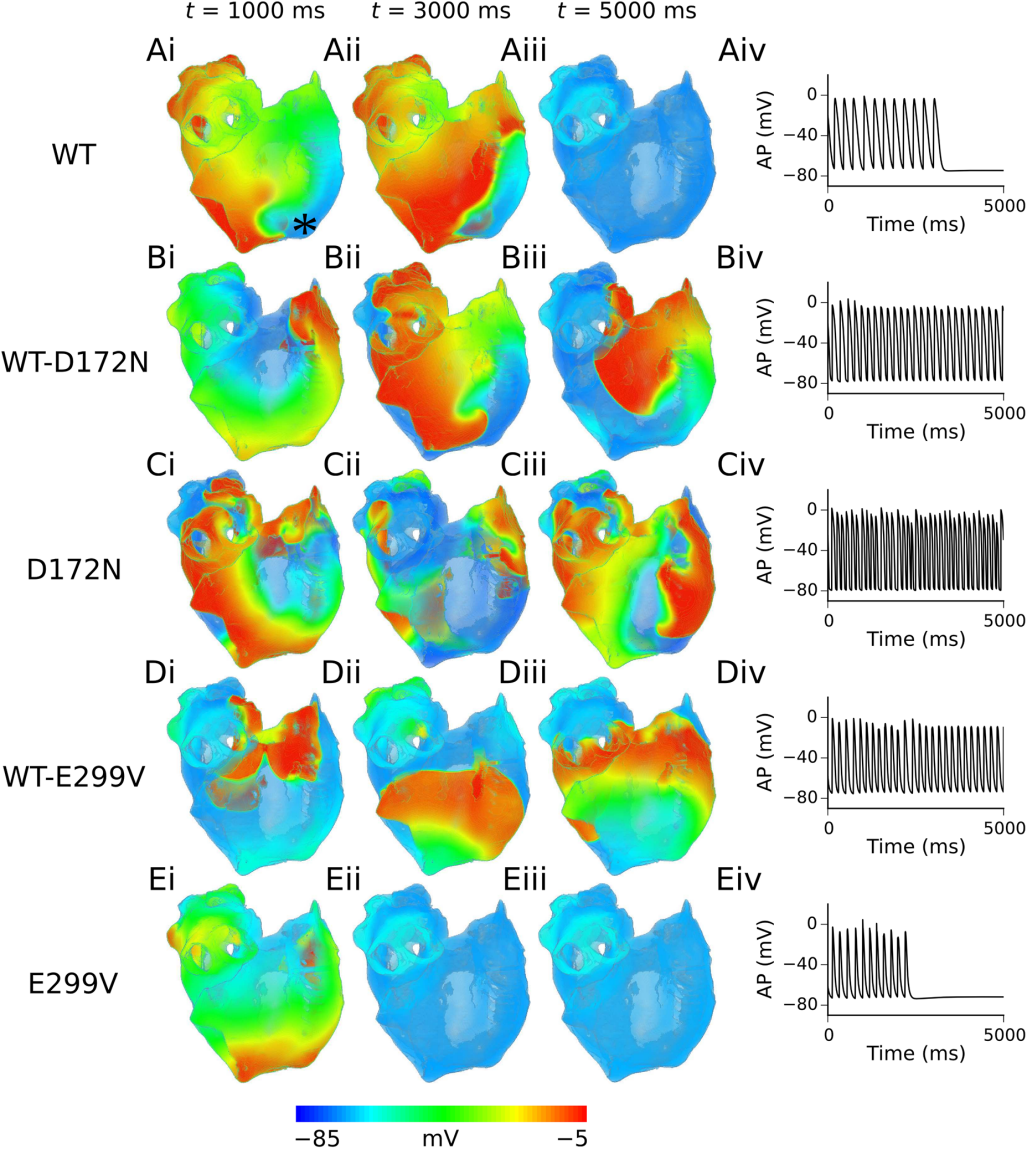
Scroll waves and corresponding APs in 3D virtual human atria. Evolution of scroll waves following initiation of re-entry, from time *t* = 1000 ms (i) to *t* = 3000 ms (ii) to *t* = 5000 ms (iii) in WT (A), WT-D172N (B), D172N (C), WT-E299V (D), and E299V (E) conditions, with corresponding APs extracted from the RA (recording location is marked with an asterisk in panel Ai) (iv). The viewpoint is looking at the RA posterior wall.

The results of 3D simulations, where re-entrant scroll waves were initiated using the phase distribution method, are summarised in Fig 6. Re-entrant excitation waves sustained for ~3.3 s in the WT condition, before self-terminating (S6 Video). In the heterozygous and homozygous D172N mutation conditions, re-entrant scroll waves were sustained for the 5.0 s duration of the simulation, with co-existence of multiple wavelets in the homozygous D172N mutation condition (S7 and S8 Videos). In the WT-E299V mutation condition re-entry was also sustained for the duration of the simulation, though scroll waves meandered significantly for ~2.0 s before settling into a stable, anatomical re-entry around the opening of the inferior vena cava (S9 Video). In the homozygous E299V condition, the initiated scroll wave meandered over a large area, before ultimately colliding with its own refractory tail and self-terminating at ~2.4 s (S10 Video). The DF and lifespan of re-entry, where applicable, are summarised in Table 2. It should be noted that while SQT3 mutation conditions favoured development of sustained re-entry, the resulting patterns were relatively organized and did not cause chaotic fibrillatory behaviour as seen in atrial fibrillation.

**Table 2:** A summary of dominant frequency and lifespan in 3D simulations.

	WT	WT-D172N	D172N	WT-E299V	E299V
**DF (Hz)**	N/A	5.2	7.5	5.3	N/A
**Lifespan re-entry (s)**	3.3	5.0 (sustained)	5.0 (sustained)	5.0 (sustained)	2.4

Dominant frequency (DF) was calculated for all continuous time series of APs.

### ‘Pharmacological’ investigations in the 3D virtual human atria

The effects of all drug interactions simulated on APD prolongation and dispersion (ΔAPD) at 1 Hz are summarised in S7 Fig. All reductions to potassium channel conductances investigated in the WT-D172N condition prolonged the APD to some extent, whilst also restoring ΔAPD to roughly the same level seen in the WT condition (see S8 Fig). Agonism of the L-type calcium channel prolonged APD, however, it also resulted in a significant increase in ΔAPD. In the WT-E299V condition, inhibition of potassium channels prolonged the APD but only caused a modest increase in ΔAPD. Individual or combined channel blocks involving I_K1_ resulted in incomplete excitation of the PV region. A 100% increase in I_CaL_ was effective in prolonging APD and restoring ΔAPD; however, excessive agonism of the L-type calcium channel (such as 250% in our simulations) was shown to hugely increase ΔAPD beyond the WT level, similar to the situation observed in the WT-D172N mutation condition.

**Fig 7:**
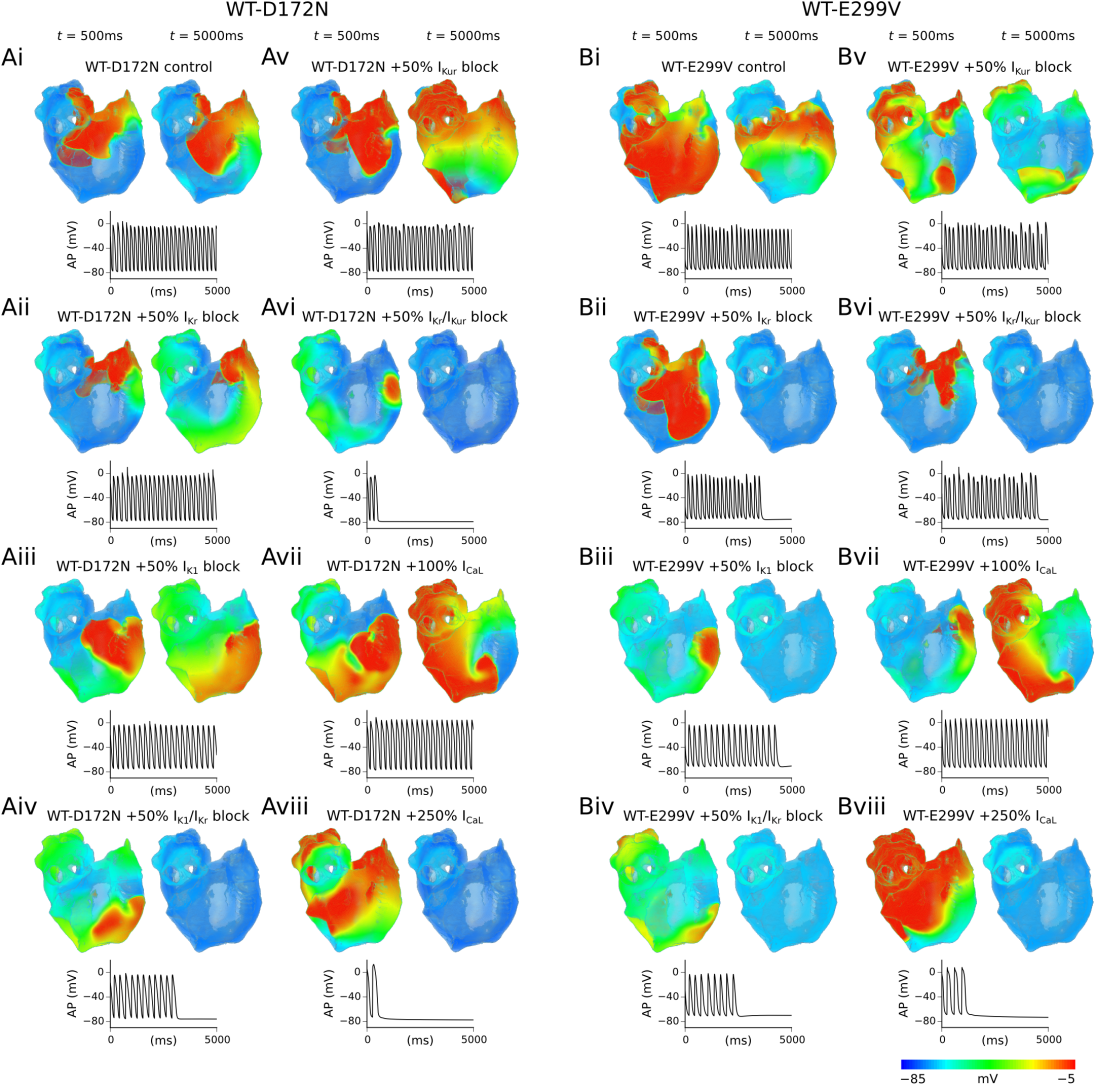
Simulated drug effects in 3D virtual human atria. Snapshots of simulated drug effects on re-entrant scroll waves in WT-D172N (A) and WT-E299V (B) conditions. For both mutation conditions panels correspond to the following cases: control (i), 50% I_Kr_ block (ii), 50% I_K1_ block (iii), combined 50% I_K1_+I_Kr_ block (iv), 50% I_Kur_ block (v), combined 50% I_Kr_+I_Kur_ block (vi), 100% increase in I_CaL_ (vii), and 250% increase in I_CaL_ (viii). For each case, two columns are shown which correspond to *t* = 500 ms and *t* = 5000 ms after initiation of re-entry, and corresponding APs extracted from the RA are shown below.

Snapshots of simulated pharmacological modulation of re-entry in WT-D172N and WT-E299V conditions are shown in Fig 7, with corresponding localised atrial excitations extracted from the RA. 50% individual reductions of I_K1_ and I_Kr_ alone were insufficient to terminate re-entry in the WT-D172N condition; although both caused a decrease in the DF (I_K1_ block produced a more significant decrease in DF–Table 3). However, combined 50% block of I_K1_ and I_Kr_ resulted in self-termination of the re-entrant wave after ~3.1 s, as shown in Fig 7. A 50% reduction in the atrial-specific I_Kur_ exhibited only a very mild anti-arrhythmic effect, reducing the DF from 5.2 Hz to 5.0 Hz but failing to terminate re-entry. When this was paired with a 50% reduction in I_Kr_, however, re-entrant excitation waves self-terminated after ~0.5 s (S11 Video).

**Table 3:** A summary of simulated drug effects in the WT-D172N and WT-E299V conditions.

	WT-D172N		WT-E299V	
	DF (Hz)	Lifespan re-entry (s)	DF (Hz)	Lifespan re-entry (s)
**Control**	5.2	5.0 (sustained)	5.3	5.0 (sustained)
**+50% I**_**K1**_ **block**	4.0	5.0 (sustained)	N/A	4.4
**+50% I**_**Kr**_ **block**	4.8	5.0 (sustained)	N/A	3.6
**+50% I**_**K1**_**/I**_**Kr**_ **block**	N/A	3.1	N/A	2.5
**+50% I**_**Kur**_ **block**	5.0	5.0 (sustained)	4.8	5.0 (sustained)
**+50% I**_**Kr**_**/I**_**Kur**_ **block**	N/A	0.5	N/A	4.5
**+100% I**_**CaL**_	4.2	5.0 (sustained)	4.2	5.0 (sustained)
**+250% I**_**CaL**_	N/A	0.6	N/A	1.5

Averaged dominant frequency (DF) and lifespan of re-entry were computed from time series of APs from several locations on the 3D human atria geometry recorded during re-entry simulations.

A 100% increase in I_CaL_ in the WT-D172N condition significantly reduced the DF of re-entry, from 5.2 Hz to 4.2 Hz, yet was insufficient to terminate re-entrant excitation waves. Increasing I_CaL_ by 250% resulted in transient re-entrant excitations which self-terminated after ~0.6 s.

The combination of 50% I_K1_+I_Kr_ reduction was successful in preventing sustained re-entry in the WT-E299V condition, as well as 50% I_K1_ reduction alone. However, measurements of the APD dispersion at a normal pacing rate showed that reducing I_K1_ in the WT-E299V condition precluded complete excitation of the PV region (S7 Fig). 50% reduction of I_Kr_, on the other hand, was also sufficient to terminate re-entry (Fig 7) whilst maintaining excitability across the entire human atria.

As in the WT-D172N condition, 50% reduction of I_Kur_ alone in the WT-E299V mutation condition demonstrated a mild anti-arrhythmic effect, causing a small decrease in DF from 5.3 Hz to 4.8 Hz which, when combined with 50% block of I_Kr_, was sufficient to terminate re-entry (S12 Video). Simulated calcium channel agonism in the WT-E299V condition also yielded similar results to the WT-D172N mutation; increasing I_CaL_ by 100% significantly reduced the DF of re-entry but was insufficient to cause termination of re-entry, whereas a 250% increase in I_CaL_ rapidly terminated re-entry. Summaries of all pharmacological investigations in the human atria in WT-D172N and WT-E299V conditions are presented in Table 3.

### Comparison with an alternative human atria model

Model-specificity of results was investigated by conducting further simulations using the Grandi *et al*. (GB) model of the human atrial AP [33]. The GB model was previously employed by Deo *et al*. in order to demonstrate human atrial APD shortening, loss of rate-adaptation of the APD, and increased vulnerability to re-entry in homogeneous tissue associated with the E299V-Kir2.1 mutation [6]. The APD shortening observed in our study in E299V mutation conditions using the GB model is consistent with that observed in the study of Deo *et al*. [6], and is qualitatively similar with our simulation results using the CZ model (see S10A Fig). Results with the two models were also similar for the D172N mutation, although this caused a larger relative degree of APD shortening in the GB model than in the CZ model, as the GB model AP exhibits a slow repolarisation tail and lack of prominent plateau phase, showing greater sensitivity to changes in repolarising I_K1_. Changes to single cell properties such as APA, RMP, and MUV observed in the GB model mirrored those found in the CZ model (see S4 Table).

The maximal slope of APD restitution was decreased in all SQT3 mutation conditions compared to the WT, as observed in the CZ model (see S10B Fig). In addition, global dispersion of APD was decreased more significantly by the E299V mutation than the D172N mutation in the GB model, reproducing another finding in the CZ model (S11 Fig). These findings are reassuring; changes to tissue excitability and stability of re-entry in SQT3 mutant I_K1_ conditions identified in the CZ model were mediated by the RMP, APD, and dispersion of APD–alterations to all of which were reproduced using an alternative, well-established human atrial cell model [33].

## Discussion

The 3D virtual human atrium is a simulation tool for the clinical study of excitation waves in the human atria in sinus rhythm and AF [23,34,35], and has been employed in several of our previous studies [23,34,36]. This study builds on previous *in silico* work [6,37,38] on investigating the substrate for AF in potassium channel-linked SQTS by utilising multi-scale idealised atrial tissue models as well as the 3D virtual human atrium, which incorporates heterogeneous atrial electrophysiology in an anatomically-realistic setting [23,34]. This study is the first to our knowledge to characterise *in silico* the effects of genetic mutations associated with *any* form of the SQTS with inclusion of intrinsic regional differences in cellular electrophysiology and anatomical details in the human atria. Furthermore, we present a theoretical exploration of pharmacological interventions for atrial arrhythmias in SQT3, which has potential clinical relevance.

### Main findings

Our major findings are as follows. (1) Heterozygous and homozygous D172N and E299V mutations shortened the APD through different mechanisms–the D172N mutations through increased outward I_K1_ during the terminal phase of repolarisation and the E299V mutations through increased I_K1_ at depolarised membrane potentials throughout the AP. (2) SQT3 mutations shortened the wavelength of re-entrant excitation waves through a reduction in both ERP and CV, which is mediated primarily by effects on the RMP that affects tissue excitability. (3) A higher degree of APD dispersion was preserved in the D172N mutations than E299V mutations, with co-existence of short APDs and larger regional APD differences leading to a more stable form of re-entry. (4) Pharmacological modulation of AF in SQT3 showed some differences between D172N and E299V mutations. However, combined I_Kr_ and I_Kur_ block emerged as a safe pharmacological option in both forms of SQT3 in our simulations.

### Pro-arrhythmic effects of SQT3 mutant I_K1_

Up-regulation of I_K1_ is an unequivocal finding in ionic remodelling of the human atrial AP associated with AF [11–14]. Computational studies of human atrial cells have suggested this to be the primary determinant of APD shortening underlying self-perpetuation of AF [15], as well as an important determinant of rotor stability and frequency in tissue [16]. An *in silico* investigation of the familial AF V93I-Kir2.1 mutation to I_K1_ by Kharche *et al*. [17] showed that both heterozygous and homozygous forms of the mutation (which increased both inward and outward I_K1_) shortened APD, hyperpolarised RMP, flattened restitution curves of APD, ERP, and CV, and stabilised rotors in the 3D virtual human atria. Unlike the present investigation, however, that study [17] lacked incorporation of heterogeneity of human atrial electrophysiology, which has been found to be an intrinsic characteristic of the atria [19–22].

In this study, differences in the I_K1_ I-V relationship caused by the SQT3 mutations were investigated, shown in Fig 1, manifesting differences in the way the mutations affected the AP. The SQT3 proband identified in [4], who was heterozygous for the D172N Kir2.1 mutation, presented with a history of presyncope and palpitations, which are often caused by supraventricular tachycardias originating in the atria [39]. In our simulations, the D172N mutation decreased overall ΔAPD by only a small degree in the 3D virtual human atria, whilst preserving a high degree of heterogeneity at the PV/LA junction, and increasing ΔAPD at the CT/PM junction (S3 Fig). It has previously been shown that short APDs co-existing with regional differences in APD creates a substrate favourable to the sustenance of re-entry in human [23] and canine [24] AF remodelled atria. Our results are consistent with this finding, as both the heterozygous and homozygous D172N mutation conditions were capable of sustaining re-entry in the 3D virtual human atria. In our previous study of SQT3 and ventricular repolarisation, the D172N-Kir2.1 mutations were shown to significantly shorten APD and QT interval in a computational model of human ventricular myocytes, accelerating and stabilising re-entrant excitation waves in the human ventricles [40]. As in the present study, electrical heterogeneities were shown to play an important role in the arrhythmia substrate, as augmented transmural dispersion of APD at localised regions of the ventricles led to increased temporal vulnerability to the genesis of uni-directional conduction block and spatial vulnerability to the initiation and sustenance of re-entry.

The E299V mutation differed from the D172N mutation in that it shortened the AP by increasing outward I_K1_ throughout the duration of the AP, not just during terminal repolarisation. In the heterozygous form of the mutation, this manifested itself as a small hyperpolarisation of the RMP, reduced APA and MUV, and dramatically shortened APD_90_. These findings are in qualitative agreement with a recent study which investigated the effects of injecting virtual ‘wild-type’ I_K1_, ‘loss-of-function’ I_K1_ associated with Andersen-Tawil syndrome, and ‘gain-of-function’ heterozygous E299V mutant I_K1_ via dynamic clamp on the action potential profile of human induced pluripotent stem cell derived cardiomyocytes [41].

The SQT3 proband in [6], who was heterozygous for the E299V-Kir2.1 mutation, presented with multiple episodes of paroxysmal AF. In our simulations the E299V mutation conditions significantly reduced APD heterogeneity across the human atria, whilst affecting heterogeneity at critical junctions differentially. Whereas the profound loss of overall ΔAPD in the pure E299V condition resulted in only transient re-entrant wave behaviour in 3D simulations, the intermediate loss of APD heterogeneity combined with abbreviated APD resulted in sustained re-entry in the WT-E299V condition. Interestingly, in our 2D simulations which were electrically-homogeneous, re-entry sustained in 3/5 simulations in E299V mutant tissue and the area of meander was comparable to the WT-E299V mutation condition (see S6 Fig), suggesting APD heterogeneity played an important role in re-entry dynamics in the 3D anatomical atria. The difference in effects of the homozygous E299V mutant conditions in 2D and 3D simulations underscores the value of conducting simulations at both levels. The long term behaviour of scroll waves in the 3D virtual human atria was not investigated; it can, however, be speculated that the combination of significant drift and reduced APD heterogeneity decreases the long-term stability of re-entrant circuits in tissue which increases the likelihood of eventual self-termination of re-entrant activity, mimicking the paroxysmal AF of the proband in [6]. This is different to the more stable form of re-entry predicted for the D172N mutation conditions (especially homozygous D172N which showed stationary meander patterns–see S5 Fig and S3 Video).

Not only does a large APD dispersion facilitate the sustenance of re-entrant excitations as suggested by our simulations, but significant regional differences in APD facilitate the *genesis* of re-entrant excitations, as temporal vulnerability of tissue to uni-directional conduction block is increased. This is consistent with prior experimental evidence that large dispersion of APD in the atria increases susceptibility to re-entry through local conduction blocks [42,43]. This suggests that large regional differences in ΔAPD associated with the SQT3 mutations increase the susceptibility to the initiation of re-entrant arrhythmias. This was confirmed in supplementary simulations by an increase in the temporal window of vulnerability to uni-directional conduction block at the CT/PM junction associated with all SQT3 mutation conditions, as shown in S4 Fig.

In addition to multiple pro-arrhythmic effects identified in the SQT3 mutations, both the D172N and E299V mutations were shown to flatten the restitution of APD, which may have a mixed anti- and pro-arrhythmic effect. Whereas steep restitution slopes facilitate alternans at the cellular level leading to wave-break in tissue, flattened restitution curves represent a loss of rate-adaptation, which can facilitate maintenance of high frequency excitation waves [17,36,44]. In the case of the D172N mutation, this finding is at variance with that reported in our previous study of human ventricles, in which simulated restitution curves were steepened [40]. The reason for this difference is not completely clear. It is likely due to differences in the models used, as this study used an atrial model and our previous study used a ventricle model [40]. It is known that there are atrio-ventricular differences in the relative roles of I_K1_, i.e. I_K1_ has a much larger current density in the ventricles [2,45], which is likely to affect responses of the models to ‘gain-in-function’ mutations to I_K1_. By way of further comparison, in our previous study of a *KCNJ2* mutation associated with familial AF, both the Kir2.1 V93I mutation which increases outward I_K1_ and a simple linear increase in maximal conductance of I_K1_ were shown to flatten APD and ERP restitution curves [17], consistent with observations in the present study.

### Pharmacological effects on re-entry in 3D virtual human atria

Use of the 3D virtual human atria in this study was extended in order to glean some insight into potential pharmacological targets in the treatment of SQT3-mediated AF. To date, no specific blockers of I_K1_ are in clinical use. In the case of the WT-D172N mutation, combined 50% reduction of I_K1_ and I_Kr_ was shown to be superior to 50% reduction of either I_K1_ or I_Kr_ alone in terminating re-entry. Chloroquine, which has been shown to be an effective inhibitor of D172N mutant Kir2.1 channels [26,46,47], blocks Kir2.1 and hERG channels at overlapping concentrations [26]. The results of our modelling thus provide evidence that a drug such as chloroquine, which inhibits both I_K1_ and I_Kr_, may be effective in treating AF associated with the D172N mutation. Previous modelling studies have highlighted the potential of chloroquine to normalize ventricular repolarisation in D172N Kir2.1-linked SQT3 and to terminate rotors in paroxysmal and chronic AF, which are associated with increased I_K1_ [47,48]. Our findings are also consistent with a study by Noujaim *et al*. [49], in which chloroquine was shown to successfully terminate re-entry associated with up-regulated Kir2.1.

In the case of the WT-E299V mutation condition, the combination of 50% block of I_K1_ and I_Kr_ was effective in preventing sustained re-entry, suggesting that an agent combining these actions may also be effective in this form of SQT3. However, I_K1_ block in the WT-E299V condition, either alone or combined with I_Kr_ block, was also shown to cause incomplete excitation of the pulmonary vein region at a normal pacing rate. This could potentially result in further abnormalities of conduction or provide a re-entrant circuit for excitation waves around the partially inexcitable pulmonary veins, suggesting I_K1_ blockers may be unsuitable for management of AF mediated by the E299V-Kir2.1 mutation.

A 50% reduction in I_Kr_ alone was also sufficient to terminate re-entry in the WT-E299V mutation condition. At the single-cell level 50% I_Kr_ block had a relatively modest effect on the baseline APD (S7 Fig). This finding could, therefore, be a consequence of the inherently unstable nature of re-entry associated with the WT-E299V mutation, which is associated with paroxysms of AF [6]. Measurement of APD dispersion at 1 Hz (S7 Fig) revealed that the PV region was affected quite significantly by blocking I_Kr_ (this region has higher expression of I_Kr_ [20,21]), potentially de-stabilising circuits around this region. Again, this highlights the usefulness of conducting multi-scale simulations, as changes to the APD at the single cell level may not necessarily be a good predictor of drug effects in electrically-heterogeneous, realistic geometries [24]. Notably, the SQT3 proband in [6] was given amiodarone, which exerts its class III anti-arrhythmic action predominantly through block of I_Kr_ [50], to effectively control AF.

Whereas a selective reduction in I_Kur_ was unable to terminate re-entrant excitation waves, combined block of I_Kr_ and I_Kur_ was shown to be effective in terminating re-entry in both WT-D172N and WT-E299V mutation conditions. Combined inhibition of I_Kr_ and I_Kur_ has previously been shown to cause a synergistic increase in APD in a mathematical model of AF-remodelled human atrial cells [51]. As I_Kur_ is not expressed in ventricles, this combination would be expected to exert a greater APD prolongation in the atria, making it more of an atrial-specific preventative measure against AF, which was the primary clinical presentation of the proband identified in [6].

Deo *et al*. [6], who first identified the E299V mutation in KCNJ2, suggested that an I_CaL_ activator of the BayK 8644 type may have some therapeutic value in this form of SQT3. We tested this hypothesis in the 3D virtual human atria by increasing maximal conductance of I_CaL_. A 100% increase in I_CaL_ significantly reduced the DF of re-entrant AP waveforms, from 5.3 Hz to 4.2 Hz in the WT-E299V condition. A 200% increase in I_CaL_ further reduced the DF to 3.8 Hz, yet was still insufficient to terminate re-entrant wave behaviour. We found that the increase in maximal I_CaL_ conductance necessary to terminate re-entry in the WT-E299V condition (250% in our simulations) significantly depolarised the RMP, reduced MUV, and significantly increased global APD dispersion in the human atria (S8 Fig). Deo *et al*. reported that increasing I_CaL_ to the level necessary to restore the APD produced unphysiologically large calcium transients in a model of human ventricular myocytes [6], which is likely to significantly increase hypertensive risk. Furthermore, such a large increase in inward calcium currents could promote a torsadogenic effect in the ventricles. Effects of simulated I_CaL_ agonists in the WT-D172N condition were qualitatively similar to those obtained in the WT-E299V mutation condition in that a high degree of I_CaL_ agonism demonstrated anti-arrhythmic effects but profoundly increased dispersion of APD across the human atria. Our simulations thus provide further evidence that I_CaL_ agonists may be undesirable therapeutic candidates for AF mediated by both forms of SQT3 investigated.

### Limitations

Limitations of the CZ model and 3D virtual human atria, such as the validation of regional cell models using primarily canine data and lack of a complete tissue fibre structure, have been discussed in detail elsewhere [23,34]. This study aimed to investigate arrhythmia substrates arising from purely cellular electrophysiological differences in SQT3 *KCNJ2* mutations, and as such did not consider effects of electrical or intracellular gap junction remodelling or fibrosis. Evidence of electrical and structural remodelling in SQT3 patients is currently lacking; nevertheless, these factors may contribute to the arrhythmia substrate [23,52] and give rise to multiple wavelets characteristic of AF. A further limitation may come from the fact that cardiac mechanical contraction was not considered, which, for ventricular function at least, has previously been suggested to be affected in SQTS patients [53–55], and may affect re-entrant wave dynamics [56,57].

The I_K1_ formulations in this study are based on current recordings from Kir2.1 channels. The WT I_K1_ formulation recapitulated the most salient difference between human atrial and ventricular I_K1_, i.e. the vastly reduced maximal current density [2,45] (compared with our previous study in human ventricles [40]), whilst retaining a high degree of similarity in kinetics with leading human atria models [30,33,58] (see S12 Fig). Native I_K1_ is carried by homo- or heteromeric assemblies of each of the Kir2.x (Kir2.1, Kir2.2, Kir2.3) subunits expressed in cardiac myocytes [59]. Human atrial I_K1_ has been reported to show weaker rectification than its ventricular counterpart [45], suggesting the possibility of a more prominent than in ventricle role for the Kir2.3 isoform [59]. However, whereas the Kir2.3 protein has been shown to be more abundant in human atrium than ventricle (10% versus <1%), the Kir2.1 protein was nevertheless measured to have the highest relative abundance amongst Kir2.x isoforms in human atrium (81% versus 92% in ventricle) [2]. Interestingly, a recent study found that altering the I-V profile of I_K1_ to resemble Kir2.3 in mathematical tissue models of the human atrial PV/LA junction did not affect the role that I_K1_ played in determining gradients of sodium availability, MDP, and MUV [60] which dictate drift direction; this suggests that any alteration to re-entry dynamics by incorporation of a Kir2.3-like component would be minimal, and thus unlikely to change the fundamental conclusions drawn in our study.

### Conclusions

The findings of this study add to the growing weight of evidence implicating increased I_K1_ in increased susceptibility to the initiation and maintenance of AF, and further highlight the influence of regional heterogeneities in overall response to gain-of-function mutations that increase I_K1_. Functional differences exist between the effects of SQT3-related D172N and E299V mutations to I_K1_ on human atrial electrophysiology: distinct mechanisms of APD shortening between D172N and E299V mutants at the single cell level manifested themselves as differences in how the mutations affected APD dispersion in the human atria and influenced re-entry dynamics. Heterozygous and homozygous forms of the D172N mutation resulted in co-existence of short APDs with marked regional heterogeneities which facilitated maintenance of re-entrant excitation waves in the human atria. In contrast, the E299V mutation presented with more unstable re-entrant wave behaviour due to significantly reduced global APD dispersion. Combined reduction of I_K1_ and I_Kr_, mimicking the effects of chloroquine, was established as an effective inhibitor of WT-D172N mutant I_K1_ in 3D re-entry simulations, whereas class III anti-arrhythmic agents which block I_Kr_ may offer a safer alternative to I_K1_ block in the WT-E299V mutation. In both SQT3 mutation conditions, I_Kr_ and I_Kur_ block combined synergistically to increase APD and terminate re-entrant wave behaviour.

## Methods

### I_K1_ and human atrial cell models

A contemporary human atrial cell model developed by Colman *et al*. [23] (CZ model) was used for the simulations in this study. The original equations for I_K1_ in the CZ model are native to the parent Courtemanche-Ramirez-Nattel (CRN) model [58]. In this study, a WT formulation of I_K1_ was developed based on WT I_Kir2.1_ currents recorded in Chinese Hamster Ovary cells at physiological temperature [26], and the maximal conductance chosen in order to give AP characteristics such as APD and RMP within the experimental range measured for human atrial myocytes (S1A Fig). I_K1_ formulations for heterozygous (WT-D172N) and homozygous (D172N) SQT3 mutants were also developed based on fitting voltage clamp data from [26], and validated using AP clamp data [26]. The waveform used, which is the same as in [26], had small step-changes in the voltage during late repolarisation, which was reflected in both the experimental and simulated AP clamp current traces at corresponding time points. Data from [6] (kindly provided by Deo *et al*.) were used to describe homozygous E299V and heterozygous WT-E299V mutant channels. The Nelder-Mead simplex algorithm [61] was used to minimise the least-squared difference between simulated and experimental I-V relationship data for all mutation conditions. Simulated I-V relationships (and AP clamp data [26] for the D172N mutation) are shown in Fig 1, as well as the *g*_K1_-V relationship computed from the same data. Details of model equations are given in Supporting S1 Text (S1 Table).

### APD restitution

Restitution of the steady-state APD at 90% repolarisation (APD_90_) was recorded in a single cell model, with multiple conditioning stimuli (of amplitude 20 pA/pF and duration 2.0 ms) employed until a stable solution was reached. A standard S1-S2 protocol was used in order to calculate the restitution of the APD against DI, details of which can be found in Supporting S1 Text. The maximal slope of restitution was calculated as the maximum ΔAPD/ΔDI.

### Tissue simulations

Propagation of APs in tissue was described using the monodomain equation [62],

$$\frac{\partial V}{\partial t}=\nabla ∙D(\nabla V)-\frac{\left(I_{\text{ion}}+I_{\text{stim}}\right)}{C_{\text{m}}},$$ (1)

where *V* is the transmembrane voltage, ***D*** is the diffusion coefficient tensor, *I*_ion_ is the total ionic current, *I*_stim_ is an externally-applied stimulus current used to initiate APs, and *C*_m_ is the membrane capacitance. Eq (1) was discretised in space using a central differences finite difference method as used in previous modelling studies [23,34,63], and integrated at each time step using a combination of the Rush-Larsen method [64] for gating variables and the forward Euler method for the cellular membrane potential. In tissue simulations, ***D*** was set to 0.21 mm^2^ms^−1^ with a 9-fold increase along the fibre direction [23,34,63] in order to give values of AT and CV consistent with experimental measurements [28,29].

In multicellular tissue simulations where sustained re-entry was initiated, a power spectrum was obtained through Fourier transform analysis of time series of APs recorded from tissue. The DF was computed using Matlab based on the largest peak in the power spectrum density. This simple approach to computing the DF, which has been described in our previous studies [17,23], is different from the more commonly used definition (e.g. see Ng *et al*. [65]).

### Tissue restitution properties

Tissue restitution properties for WT and SQT3 mutant I_K1_ channels were determined using a 1D strand model with spatial step 0.25 mm and length 25 mm. After being paced until a stable solution was reached in a single cell environment, the 1D strand was paced using these initial conditions from one end with five consecutive conditioning S1 stimuli of spatial size 2.5 mm before application of an S2 stimulus and subsequent analysis of the final two APs (to account for APD alternans [66]). Using this protocol, restitution curves for the CV, ERP, and re-entry WL were computed.

The ERP was defined as the minimal S1-S2 coupling required to elicit an AP which propagated at least three-quarters (about 16.25 mm) of the distance down the strand. The CV was determined by dividing the distance of the centre section of the strand (measured at nodes one and three quarters along the strand) by the difference in activation times. WL was computed using WL = CV × ERP. The excitation threshold, a measure of atrial tissue excitability, in each of the WT/mutant cases was determined as described previously [17,40]. Briefly, the 1D strand model was paced using a standard S1-S2 protocol for five beats, following which a stimulus of constant duration but variable amplitude was applied, and the minimum amplitude required to elicit an AP which propagated for a given S2 was recorded as a measure of tissue excitability.

### Re-entry in a 2D sheet

In supplementary simulations, spiral wave dynamics were investigated using an idealised 2D sheet of isotropic, electrically-homogeneous tissue with dimensions 100 × 100 mm^2^ and spatial step 0.25 mm. A cross-field S1-S2 stimulation protocol was used to initiate re-entrant spiral waves, as in previous modelling studies [17,37]. After being paced until a stable solution was reached in a single cell environment, the 2D tissue patch was then paced using these initial conditions from one edge with four consecutive conditioning S1 stimuli before application of an S2 stimulus of area 50 × 50 mm^2^ in the lower left quadrant of the patch. The S2 stimulus was applied at variable times after the ERP, inducing a wave which propagated uni-directionally and subsequently developed into a spiral wave. The cores of spiral waves were tracked using the method of locating phase singularities [67], and the lifespan and area of meander computed as measures of the stability of re-entry.

### Simulations in 3D anatomical human atria geometry

In order to characterise the behaviour of re-entrant excitation waves in an anatomically-realistic setting, the 3D virtual human atrium [34], which is based on the visible human dataset [63], was employed, as in several of our previous modelling studies [23,34,36]. The 3D geometry was taken to be electrically heterogeneous, as it is segmented into distinct regions of the atria, described fully in [23] and shown in Fig 2. The CZ model incorporates a family of regional cell models, accounting for distinct electrophysiological differences in the right atrium (RA), left atrium (LA), right atrial appendage (RAA), left atrial appendage (LAA), crista terminalis (CT), pectinate muscles (PM), atrio-ventricular ring (AVR), atrial septum (AS), Bachmann’s bundle (BB), and pulmonary veins (PV). Details are provided in Supporting S1 Text, along with S1B Fig which shows model validation of regional cell models, and S2 Fig which gives a detailed validation of the PV model. In addition, a degree of fibre anisotropy along the bundles of the CT, PM, and BB is included. Re-entry was initiated using the phase distribution method [68], whereby an artificial asymmetric conduction pattern is created, leading to the development of a 3D spiral (scroll) wave (see S9 Fig and Supporting S1 Text for details). Where applicable, averaged DF and lifespan of re-entry were calculated based on time series of APs taken from several locations on the human atria geometry.

### Investigating ion channel targets in SQT3-mediated AF

In order to investigate potential pharmaceutical strategies in SQT3-induced AF consequent to abbreviated APD, effects of prolonging APD via modulating variant ion channels on atrial excitation were investigated. This was done by either inhibiting potassium channel currents or augmenting the L-type calcium channel current that prolongs the repolarising phase of the cellular AP. Seeing that SQTS mutations are expressed heterozygously *in vivo*, we only investigated pharmacological modulation of the WT-D172N and WT-E299V mutants–inhibiting potassium channel currents (namely I_K1_, I_Kr_, and I_Kur_) by decreasing their maximal channel conductance (as done in previous studies [24,50,69]) or increasing I_CaL_ (mimicking the effects of a calcium channel agonist [70]). As multichannel agents may show superiority in anti-arrhythmic effects over single channel modulators [71–74], combined single and multiple ion channel blockers were screened, evaluating their actions first on tissue APD dispersion in the 3D virtual human atria, and then on the dynamic behaviours of re-entrant excitation waves initiated by the phase distribution method [68] in the 3D model. In these simulations, the DF and lifespan of re-entry were calculated, where applicable, as indicators of the efficacy of ion channel block combinations.

### Comparison with an alternative human atria model

To draw model independent conclusions, comparative simulations were also performed using the GB model of the human atrial AP [33] as used in [6], with simplifications to Ca^2+^ handling described in [75]. The model is mostly derived from human ventricular data [76], and thus exhibits different properties to the CZ model. The GB has a type 3 AP morphology [77], compared to the type 1 AP morphology of the model used in the present study. The aforementioned newly-developed formulations of WT and SQT3 mutant I_K1_ were incorporated into the GB model, and the conductances scaled as in the CZ model. Any additional changes to the baseline model are detailed in Supporting S1 Text.

## Supporting information

S1 Text Detailed information on development of simulation tools and supplementary investigations. (DOCX)

S1 Fig Comparison of baseline and regional cell model APs with experimental data. (Ai) A comparison of the baseline right atrium (RA) model AP used in this study with experimental recordings of action potentials taken from human RA myocytes (Bosch *et al*., 1999; Wang *et al*., 1993; Wettwer *et al*., 2004; Koumi *et al*., 1995; Workman *et al*., 2001). (Aii) Validation of baseline model against experimentally-measured metrics; action potential amplitude (APA), maximum upstroke velocity (MUV), action potential duration at 50% and 90% (APD_50_ and APD_90_, respectively), and resting membrane potential (RMP), using data from Gong *et al*., 2008; Poulet *et al*., 2015; Hordof *et al*., 1976; Gelband *et al*., 1972; Pau *et al*., 2007; Redpath *et al*., 2006; Katoh *et al*., 2005; Bosch *et al*., 1999; Dobrev & Ravens, 2003; Kim *et al*., 2002). (Bi) Regional cell model action potentials from the crista terminalis (CT), right atrial appendage (RAA), atrio-ventricular ring (AVR), atrial septum (AS), Bachmann’s bundle (BB), left atrium (LA), pectinate muscles (PM), and RA. (Bii) Comparison of APD_90_ ratios (APD_95_ for Feng *et al*., 1998) in regional cell models using experimental data from Gong *et al*., 2008; Katoh *et al*., 2005; Feng *et al*., 1998; Burashnikov *et al*., 2004; Li *et al*., 2001.
(DOCX)

S2 Fig Validation of the pulmonary vein model. (A) A comparison of model changes in maximal ionic conductances of I_to_, I_Ks_, I_Kr_, I_K1_, and I_CaL_ between PV and LA models against experimental measurements. (B) Comparison of AP characteristics, namely APD_90_, MUV, and APA, between PV and LA models against experimental data. (C) Action potentials from the LA and PV models at a pacing frequency of 2 Hz compared with experimentally-recorded APs from canine atrial myocytes (Cha *et al*., 2005) shown inset. (D) Comparison of difference in RMP between LA and PV models against experimental measurements. All experimental data are taken from Ehrlich *et al*., 2003; Datino *et al*., 2010; Cha *et al*., 2005).
(DOCX)

S3 Fig Differences in regional cell model tissue APD. ΔAPD at the CT/PM and PV/LA junctions as determined in 1D tissue models.
(DOCX)

S4 Fig Temporal vulnerability window to uni-directional conduction block. Space-time plots of AP propagation in 1D models of the CT/PM junction used to compute temporal vulnerability to re-entry and corresponding vulnerability window (VW) widths. Three scenarios are shown which correspond to different S2 timings: bi-directional conduction block (A), uni-directional conduction (B), and bi-directional conduction (C). A summary of VW measurements in WT and SQT3 mutation conditions (D).
(DOCX)

S5 Fig Re-entry simulations in idealised 2D sheet. A summary of rotor trajectories in 2D re-entry simulations for different S2 timings after the effective refractory period (ERP).
(DOCX)

S6 Fig Spiral wave characteristics in 2D re-entry simulations. (A) Bar charts showing the average lifespan of re-entrant excitations in 5 re-entry simulations corresponding to 5 different S2 timings; and (B) the average area of meander over time.
(DOCX)

S7 Fig APD dispersion in 3D geometry under simulated pharmacological modulation conditions. (A) WT ΔAPD is shown for reference. ΔAPD in WT-D172N (B) and WT-E299V (C) tissue is shown under the following conditions: (i) control, (ii) 50% I_Kr_ block, (iii) 50% I_K1_ block, (iv) 50% I_K1_+I_Kr_ block, (v) 50% I_Kur_ block, (vi) 50% I_Kr_+I_Kur_ block, (vii) 100% increase in I_CaL_, and (viii) 250% increase in I_CaL_. The colour bar shows APD relative to the shortest APD measured in each condition, designated APD+. The scale of the colour bar is fixed at the value of ΔAPD in the WT condition (120 ms). Regions where APs failed to exceed −20 mV are shown in black. Single cell AP traces at 1 Hz pacing are shown in WT-D172N (Di, Dii) and WT-E299V (Diii, Div) conditions.
(DOCX)

S8 Fig Summary of ΔAPD and relative APD prolongation in pharmacological simulation conditions. Floating bar charts showing ΔAPD in WT-D172N (A) and WT-E299V (B) mutant tissue under various simulated drug effect conditions. The WT ΔAPD is shown in light blue for reference.
(DOCX)

S9 Fig Illustration of the phase distribution method and evolution of scroll waves. (A) Mapping of the membrane potential from an action potential onto the realistic 3D human atria geometry. (B) Evolution of scroll waves in the RA at different time points using phase distribution method initial conditions.
(DOCX)

S10 Fig AP and I_K1_ profile in WT and mutation conditions using the GB model. Action potential waveforms in WT, WT-D172N, D172N, WT-E299V, and E299V conditions at a pacing frequency of 1 Hz (Ai), with corresponding current trace for I_K1_ (Aii). Restitution of the APD_90_ (Bi), and maximal slope of restitution (Bii).
(DOCX)

S11 Fig Regional cell models and spatial dispersion of APD in GB model. Regional cell models in the GB model (A), including PM, RAA, CT, BB, AS, LA, LAA, AVR, PV. APD distribution maps in WT (Bi), WT-D172N (Bii), D172N (Biii), WT-E299V (Biv), and E299V (Bv) mutation conditions, with corresponding ΔAPD (C). The colour bar shows APD relative to the shortest APD measured in each condition, designated APD+. The scale of the colour bar is fixed at the value of ΔAPD in the WT condition (72 ms). The colour black shows regions where membrane potentials failed to exceed a threshold value (−20 mV).
(DOCX)

S12 Fig Comparison of I_K1_ kinetics with previous studies. (A) Simulated I-V relationships for the WT I_K1_ formulation used in the human atrial cell model in the present study compared with the WT I_K1_ formulation used in our previous study in human ventricular cells (Adeniran *et al*., 2012)–the inset shows native I_K1_ recordings in human atrial and ventricular myocytes taken from Wang *et al*., 1998. (B) Simulated I-V relationships for I_K1_ used in this study compared with I_K1_ from the GPB model (Grandi *et al*., 2011), CRN model (Courtemanche *et al*., 1998), and the NFG model (Nygren *et al*., 1998).
(DOCX)

S1 Table I_K1_ formulation parameters. Parameters of I_K1_ for WT, WT-D172N, D172N, WT-E299V, and E299V mutation conditions, obtained by fitting Equation S1 to experimental data (El Harchi *et al*., 2009; Deo *et al*., 2013). For comparison, formulations of the WT model used in Kharche *et al*., 2008; and the CRN model I_K1_ (Courtemanche *et* al., 1998) are shown.
(DOCX)

S2 Table Ionic differences in regional cell models. A summary of conductance scaling factors, G_X_, for maximal conductance of ionic current I_X_ relative to the baseline (RA) cell model and corresponding experimental data sources. Abbreviations are as follows: CT = crista terminalis, BB = Bachmann’s bundle, PM = pectinate muscles, AVR = atrio-ventricular ring, RAA = right atrial appendage, AS = atrial septum, LA = left atrium, LAA = left atrial appendage, PV = pulmonary veins.
(DOCX)

S3 Table Dominant frequency in 2D re-entry simulations. A summary of dominant frequencies (DF) in SQT3 mutation conditions in a representative 2D spiral wave re-entry simulation.
(DOCX)

S4 Table AP properties in WT and SQT3 mutant conditions in the GB model. A summary of AP properties such as action potential amplitude (APA), resting membrane potential (RMP), action potential duration at 50% and 90% repolarisation (APD_50_ and APD_90_, respectively), and maximum upstroke velocity (MUV) in the GB model in WT and SQT3 mutation conditions at a pacing frequency of 1 Hz.
(DOCX)

S1 Video WT re-entry in 2D idealised geometry. A representative video of initiation and conduction of spiral waves in a 2D idealised geometry in the WT condition. Re-entry was induced using an S1-S2 protocol: following propagation of a planar wave elicited with four conditioning S1 stimuli at a BCL of 400 ms, an S2 stimulus was applied 40 ms after the effective refractory period in the lower left quadrant of the patch. The initiated spiral wave meanders out of the tissue boundaries in <200 ms. The evolution of spiral wave core trajectories (marked by white circles) is superimposed onto the video.
(AVI)

S2 Video WT-D172N re-entry in 2D idealised geometry. A representative video of initiation and conduction of spiral waves in a 2D idealised geometry in the WT-D172N condition. Re-entry was induced using an S1-S2 protocol: following propagation of a planar wave elicited with four conditioning S1 stimuli at a BCL of 400 ms, an S2 stimulus was applied 40 ms after the effective refractory period in the lower left quadrant of the patch. The initiated spiral wave persists for the duration of the simulation. The evolution of spiral wave core trajectories (marked by white circles) is superimposed onto the video.
(AVI)

S3 Video D172N re-entry in 2D idealised geometry. A representative video of initiation and conduction of spiral waves in a 2D idealised geometry in the D172N condition. Re-entry was induced using an S1-S2 protocol: following propagation of a planar wave elicited with four conditioning S1 stimuli at a BCL of 400 ms, an S2 stimulus was applied 40 ms after the effective refractory period in the lower left quadrant of the patch. The initiated spiral wave persists for the duration of the simulation. The evolution of spiral wave core trajectories (marked by white circles) is superimposed onto the video.
(AVI)

S4 Video WT-E299V re-entry in 2D idealised geometry. A representative video of initiation and conduction of spiral waves in a 2D idealised geometry in the WT-E299V condition. Re-entry was induced using an S1-S2 protocol: following propagation of a planar wave elicited with four conditioning S1 stimuli at a BCL of 400 ms, an S2 stimulus was applied 40 ms after the effective refractory period in the lower left quadrant of the patch. The initiated spiral wave persists for the duration of the simulation. The evolution of spiral wave core trajectories (marked by white circles) is superimposed onto the video.
(AVI)

S5 Video E299V re-entry in 2D idealised geometry. A representative video of initiation and conduction of spiral waves in a 2D idealised geometry in the E299V condition. Re-entry was induced using an S1-S2 protocol: following propagation of a planar wave elicited with four conditioning S1 stimuli at a BCL of 400 ms, an S2 stimulus was applied 40 ms after the effective refractory period in the lower left quadrant of the patch. The initiated spiral wave persists for >4.0 s before meandering out of the tissue boundary shortly before the end of the simulation. The evolution of spiral wave core trajectories (marked by white circles) is superimposed onto the video.
(AVI)

S6 Video WT re-entry in 3D anatomical human atria geometry. Re-entrant scroll waves in the WT condition initiated in the 3D human atria shown from two views–looking at the RA posterior wall (left) and into the cavities (right). A single scroll wave persists for ~3.3 s before colliding with its own refractory tail and self-terminating.
(AVI)

S7 Video WT-D172N re-entry in 3D anatomical human atria geometry. Re-entrant scroll waves in the WT-D172N condition initiated in the 3D human atria shown from two views–looking at the RA posterior wall (left) and into the cavities (right). A single scroll wave follows a stable trajectory in the right atrium along the junction of the crista terminalis and pectinate muscles, persisting for the 5.0 s duration of the simulation.
(AVI)

S8 Video D172N re-entry in 3D anatomical human atria geometry. Re-entrant scroll waves in the D172N condition initiated in the 3D human atria shown from two views–looking at the RA posterior wall (left) and into the cavities (right). The initial scroll wave quickly degenerates into multiple wavelets, persisting for the 5.0 s duration of the simulation.
(AVI)

S9 Video WT-E299V re-entry in 3D anatomical human atria geometry. Re-entrant scroll waves in the WT-E299V condition initiated in the 3D human atria shown from two views–looking at the RA posterior wall (left) and into the cavities (right). Scroll waves meander significantly around the right atrial appendage and superior vena caval opening for ~2.0 s, before settling into a stable, anatomical re-entry around the opening of the inferior vena cava which persists for the 5.0 s duration of the simulation.
(AVI)

S10 Video E299V re-entry in 3D anatomical human atria geometry. Re-entrant scroll waves in the E299V condition initiated in the 3D human atria shown from two views–looking at the RA posterior wall (left) and into the cavities (right). Scroll waves meander significantly around the right atrial appendage and right atrium, before self-terminating at ~2.4 s.
(AVI)

S11 Video WT-D172N +50% I_Kr_/I_Kur_ block re-entry in 3D anatomical human atria geometry. Re-entrant scroll waves in the WT-D172N +50% I_Kr_/I_Kur_ block condition initiated in the 3D human atria shown from two views–looking at the RA posterior wall (left) and into the cavities (right). The initial scroll wave quickly breaks up, de-stabilising the re-entrant circuit and resulting in self-termination of scroll waves after ~0.5s.
(AVI)

S12 Video WT-E299V +50% I_Kr_/I_Kur_ block re-entry in 3D anatomical human atria geometry. Re-entrant scroll waves in the WT-E299V +50% I_Kr_/I_Kur_ block condition initiated in the 3D human atria shown from two views–looking at the RA posterior wall (left) and into the cavities (right). The initial scroll wave breaks into multiple wavelets which persist for ~4.5 s, before self-terminating.
(AVI)
