# Correction: Atrial arrhythmogenicity of *KCNJ2* mutations in short QT syndrome: Insights from virtual human atria

**DOI:** 10.1371/journal.pcbi.1007145

**Published:** 2019-06-13

**Authors:** The PLOS Computational Biology Staff

## Notice of Republication

This article was republished on September 12, 2017, to correct an error in the author byline and article citation. The publisher apologizes for the error. Please download this article again to view the correct version.

## Supporting information

S1 File Republished, corrected article. (PDF)
