# Supplementary material for: Atrial arrhythmogenicity of KCNJ2 mutations in short QT syndrome: Insights from virtual human atria
Source: PLoS Comput Biol. 2017 Jun 13;13(6):e1005593. doi: 10.1371/journal.pcbi.1005593 (PMC5487071; doi:10.1371/journal.pcbi.1005593)
Supplement: S1 Text — (DOCX) [file pcbi.1005593.s001.docx]

**Supporting text S1**

**Atrial arrhythmogenicity of KCNJ2-linked short QT syndrome mutations: insights from virtual human atria**

Dominic G. Whittaker, Haibo Ni, Aziza El Harchi, Jules C. Hancox, Henggui Zhang

**I_K1_ formulation**

The formulation for I_K1_ used was based on our previous study [1], further modified to account for the square root dependence of I_K1_ slope conductance on [K^+^]_o_, the extracellular potassium concentration [2,3]. This dependence is considered to be a hallmark of cardiac inward rectifiers [4], and was a necessary consideration due to different potassium concentrations used in cellular electrophysiology experiments on which the mutation data was based [5,6]. I_K1_ was formulated as follows:

$I_{K1}=g_{K1}\left( V-E_{K} \right),$ where $g_{K1}=\sqrt{\frac{K_{o}}{5.4}}g_{K1,max}\left( a+\frac{(1-a)}{1+\exp(b\left( V-c \right))} \right)$ (S1)

where *E*_K_ is the reversal potential for potassium channels, *K*_o_ is the extracellular potassium concentration, and *g*_K1,max_ is the maximal conductance. Additional parameters introduced as in [1] are as follows: *a* is the fraction of the channel conductance that is voltage-independent, *b* represents the steepness of the *g*_K1_-V relationship, and *c* is the half-point of the *g*_K1_-V relationship.

The ‘wild-type’ (WT) formulation for I_K1_ was developed based on absolute magnitude I_Kir2.1_ recordings from Chinese Hamster Ovary cells at physiological temperature with [K^+^]_o_ = 4.0 mM [5]. The Nelder-Mead simplex algorithm [7] within the cross platform data analysis and visualisation software QtiPlot (free basic version available at [www.qtiplot.com](http://www.qtiplot.com)) was used to minimise a cost function defined as the least-squared difference between simulated and experimental currents in the I-V relationship [5]. Maximal WT conductance was then clamped and *a*, *b*, and *c* allowed to vary freely in order to develop formulations of I_K1_ for homozygous and heterozygous forms of the D172N mutant, with the reversal potential, *E*_rev_, fixed at −88.4 mV as measured in [5]. As in our previous study [8], maximal conductances were scaled using relative proportions of peak I_Kir2.1_ measured in AP clamp experiments [5]. Maximal conductance of WT I_K1_ was 0.118 nS/pF, compared with 0.09 nS/pF in the Courtemanche *et al*. (CRN) model [9], giving current densities within the range measured in human atrial myocytes [10]. AP clamp and I-V relation data for the D172N mutation can be found in Supplementary dataset S1.

Formulations for homozygous and heterozygous E299V mutations were developed based on data from [6] (original I-V curve data was kindly provided by Dr. Makarand Deo). The previously-developed WT formulation of I_K1_ was matched to WT I_Kir2.1_ recordings from [6], with only the maximal conductance allowed to vary (and *E*_rev_ adjusted to −86.0 mV [6]). Maximal conductance was then fixed when modifying I_K1_ parameters (*a*, *b*, and *c*) to develop formulations for heterozygous and homozygous E299V mutations. As [K^+^]_o_ = 4.8 mM was used in the cellular electrophysiology experiments, maximal conductance of I_Kir2.1_ would be expected to be ~9-10% greater than in the experiments of El Harchi *et al*. [5] based on the square root dependence of conductance on extracellular potassium. This was accounted for by lowering *g*_K1,max_ accordingly. Model parameters are detailed in Table S1.

Table S1. I_K1_ formulation parameters.

|  | ***g*_K1,max_ (nS/pF)** | ***a*** | ***b* (mV^-1^)** | ***c* (mV)** |
| --- | --- | --- | --- | --- |
| **WT** | 0.118 | 0 | 0.077 | -85.00 |
| **WT-D172N** | 0.154 | 0 | 0.0847 | -69.26 |
| **D172N** | 0.277 | 0 | 0.09317 | -62.00 |
| **WT-E299V** | 0.107 | 0.0385 | 0.0323 | -94.27 |
| **E299V** | 0.107 | 0.1456 | 0.0369 | -139.54 |
| **Kharche *et al*. WT** [1] | 3.650 | 0.0482 | 0.086 | -77.80 |
| **CRN model** [9] | 0.090 | 0 | 0.070 | -80.00 |

Parameters of I_K1_ for WT, WT-D172N, D172N, WT-E299V, and E299V mutation conditions obtained by fitting Equation S1 to experimental data [5,6]. For comparison, formulations of the WT model used in [1], and the CRN model I_K1_ are shown [9].

**Single cell models and AP simulations**

The cellular membrane potential in single cell simulations was modelled using the electric circuit model, and thus evolved according to the following differential equation:

$\frac{dV}{dt}=-\frac{I_{\text{ion}}+I_{\text{stim}}}{C_{\text{m}}}$ (S2),

where *V* is the membrane potential, I_ion_ is the total sum of ionic currents, I_stim_ is a stimulus current used to initiate APs, and C_m_ is the membrane capacitance (set to a constant value of 100 pF [11]).

The Colman *et al*. (CZ) model [11,12], was utilised in this study, which is well suited to the study of re-entrant arrhythmias. The I_K1_ equations (native to the parent CRN model [9]) were replaced with the previously-developed WT formulation of I_K1_. The baseline model was validated extensively against experimental measurements of the following metrics in human atrial myocytes: action potential amplitude (APA), maximum upstroke velocity (MUV), action potential duration at 50% and 90% repolarisation (APD_50_ and APD_90_, respectively), and resting membrane potential (RMP), as shown in Fig S1A. Experimental data are taken from [13–22].

Regional cell models were developed based on experimentally-measured changes in maximal current density of several ionic currents [11] relative to the baseline right atrium (RA) model, and validated based on regional differences in APD as shown in Fig S1B. An extensive validation of the PV model against the LA model is shown in Fig S2. Although some human data is available [13,19,23], primarily data from canine atrial myocytes was used [24–29], as this is much more readily available. Changes to maximal ionic conductances implemented were largely the same as our previous study [11], and can be seen in Table S2.

Table S2. Ionic differences in regional cell models.

|  | **G_CaL_** | **G_to_** | **G_Kur_** | **G_Na_** | **G_Kr_** | **G_Ks_** | **G_K1_** | **Source** |
| --- | --- | --- | --- | --- | --- | --- | --- | --- |
| **CT** | 1.68 | 1.0 | 1.0 | 1.0 | 1.0 | 1.0 | 1.0 | [24] |
| **BB** | 1.72 | 1.0 | 1.0 | 1.0 | 1.0 | 1.0 | 1.0 | [24,25] |
| **PM** | 0.94 | 1.0 | 1.0 | 1.0 | 1.0 | 1.0 | 1.0 | [24] |
| **AVR** | 0.67 | 0.6 | 1.0 | 1.0 | 1.63 | 1.0 | 1.0 | [24] |
| **RAA** | 1.0 | 0.68 | 1.0 | 1.0 | 1.0 | 1.0 | 1.0 | [13,24] |
| **AS** | 0.4 | 0.212 | 0.667 | 1.3 | 1.0 | 1.0 | V_1/2_ −6 | [13] |
| **LA** | 1.0 | 1.0 | 1.0 | 1.0 | 1.6 | 1.0 | 1.0 | [24,26,27] |
| **LAA** | 1.0 | 0.68 | 0.8 | 1.0 | 1.6 | 1.0 | 1.0 | [23,24,26] |
| **PV** | 0.7 | 0.75 | 1.0 | 1.0 | 2.4 | 1.5 | 0.62  V_1/2_ −7 | [27–29] |

A summary of conductance scaling factors, G_X_, for maximal conductance of ionic current I_X_ relative to the baseline (RA) cell model and corresponding experimental data sources. Abbreviations are as follows: CT = crista terminalis, BB = Bachmann’s bundle, PM = pectinate muscles, AVR = atrio-ventricular ring, RAA = right atrial appendage, AS = atrial septum, LA = left atrium, LAA = left atrial appendage, PV = pulmonary veins.


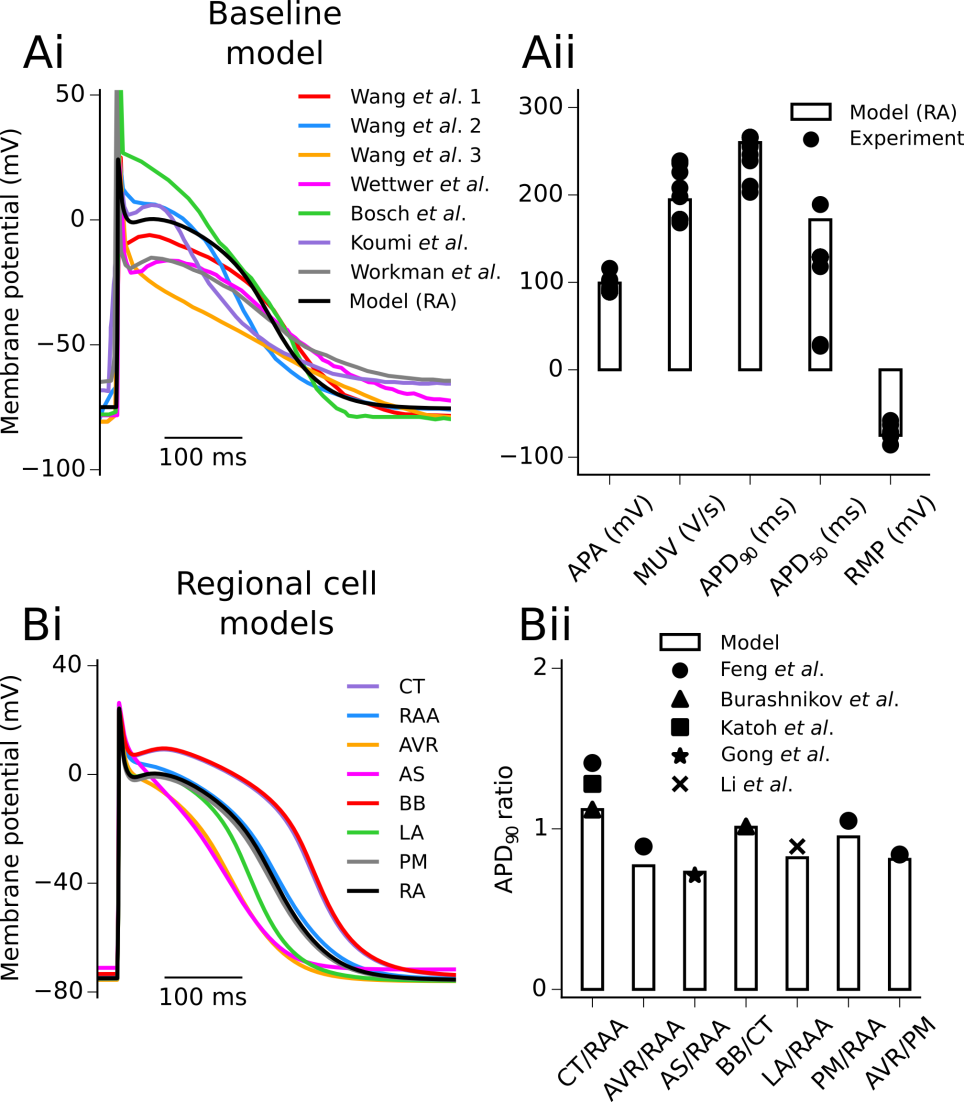


Fig S1. Comparison of baseline and regional cell model APs with experimental data. (Ai) A comparison of the baseline right atrium (RA) model AP used in this study with experimental recordings of action potentials taken from human RA myocytes [20,30–33]. (Aii) Validation of baseline model against experimentally-measured metrics; action potential amplitude (APA), maximum upstroke velocity (MUV), action potential duration at 50% and 90% (APD_50_ and APD_90_, respectively), and resting membrane potential (RMP), using data from [13–22]. (Bi) Regional cell model action potentials from the crista terminalis (CT), right atrial appendage (RAA), atrio-ventricular ring (AVR), atrial septum (AS), Bachmann’s bundle (BB), left atrium (LA), pectinate muscles (PM), and RA. (Bii) Comparison of APD_90_ ratios (APD_95_ for Feng *et al*. [24]) in regional cell models using experimental data from [13,19,24–26].


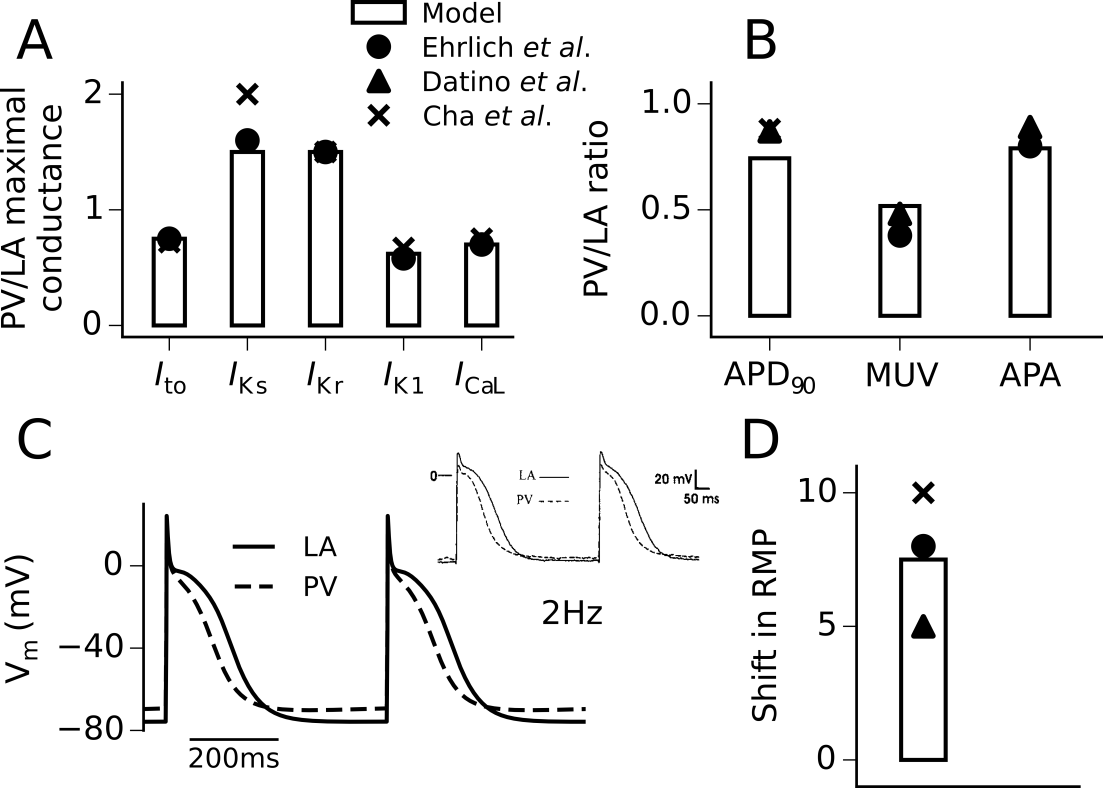


Fig S2. Validation of the pulmonary vein model. (A) A comparison of model changes in maximal ionic conductances of I_to_, I_Ks_, I_Kr_, I_K1_, and I_CaL_ between PV and LA models against experimental measurements. (B) Comparison of AP characteristics, namely APD_90_, MUV, and APA, between PV and LA models against experimental data. (C) Action potentials from the LA and PV models at a pacing frequency of 2 Hz compared with experimentally-recorded APs from canine atrial myocytes [29] shown inset. (D) Comparison of difference in RMP between LA and PV models against experimental measurements. All experimental data are taken from [27–29].

Restitution of the APD_90_ was measured using an S1-S2 protocol. In order to reach a true steady state, 100 conditioning S1 stimuli were delivered at a pacing frequency of 1 Hz (stimulus strength 20 pA/pF and duration 2.0 ms). An S2 stimulus was applied at varying intervals following the final S1 stimulus, and an APD restitution curve generated by plotting the APD_90_ against the corresponding diastolic interval (DI), which is computed as follows: DI = S2 – APD_90_. Maximum slope of restitution was determined as the maximal derivate of APD_90_ against DI.

**1D simulations**

The propagation of APs in isotropic, homogeneous 1D models (consisting of 100 nodes and no-flux boundary conditions at the edges) was governed by the 1D cable equation:

$\frac{\partial V}{\partial t}=-\frac{I_{\text{ion}}+I_{\text{stim}}}{C_{\text{m}}}+D\left( \frac{\partial^{2}V}{\partial x^{2}} \right)$ (S3),

where *D* is a scalar coefficient describing the diffusion of voltage through the medium, and all other parameters retain their previous definition. Steady state initial conditions from the single cell model between a basic cycle length (BCL) of 100 ms and 1200 ms at intervals of 10 ms were saved and read into the 1D model as required. These were used to compute steady-state restitution curves for CV, ERP, WL re-entry, and tissue excitation threshold. When measuring the ERP, a threshold of −20 mV was used to define an AP.

1D models of the CT/PM and LA/PV junctions were constructed in order to accurately measure ΔAPD at these regions in tissue. The APD in tissue was measured using a membrane potential threshold of −60 mV (adjusted in the PV model) which corresponded well to the APD_80_-APD_90_ (see Fig S3). This was done as the APD_90_ depends on APA and slope of repolarisation and so does not always accurately reflect regional differences in refractoriness in tissue such as at the LA/PV junction where there is an APA difference, or in the GB model which shows a very long repolarisation tail. The CT/PM 1D model consisted of 50 nodes representing the CT region and 50 nodes representing the PM, and was stimulated at the first 10 nodes of the CT region, eliciting an AP which propagated towards the PM. The 1D LA/PV junction model was constructed in the same way, with APs initiated in the LA region.


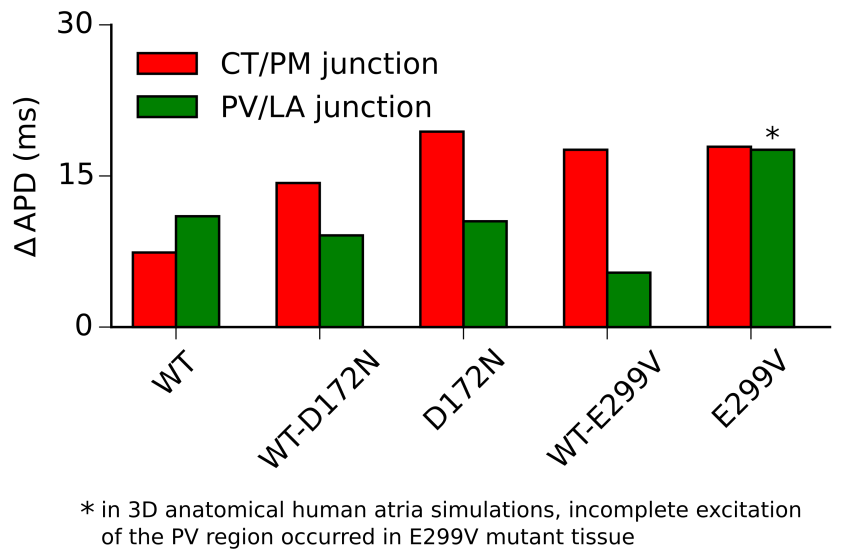


Fig S3. Differences in regional cell model tissue APD. ΔAPD at the CT/PM and PV/LA junctions as determined in 1D tissue models.

The CT/PM 1D model was employed in order to measure the temporal vulnerability window (VW) to uni-directional block [1,8]. Following propagation of a wave from the CT to PM region in 1D, application of a premature stimulus can result in one of three scenarios: (i) bi-directional conduction block if the stimulus is applied too early and occurs during the refractory tail of both regions, (ii) uni-directional conduction block if one region is still refractory but the other has recovered sufficiently to allow propagation of an AP, and (iii) bi-directional conduction if both regions of the tissue have recovered. At a time delay following the final S1 stimulus, an S2 stimulus was applied in the middle of the 1D strand (at the CT/PM junction) with the same duration and double amplitude as the S1 stimulus. The width of the temporal VW was determined by computing the difference in maximal and minimal values of S2 which induced uni-directional conduction block, as shown in Fig S4. The threshold for an AP was again defined at −20 mV.


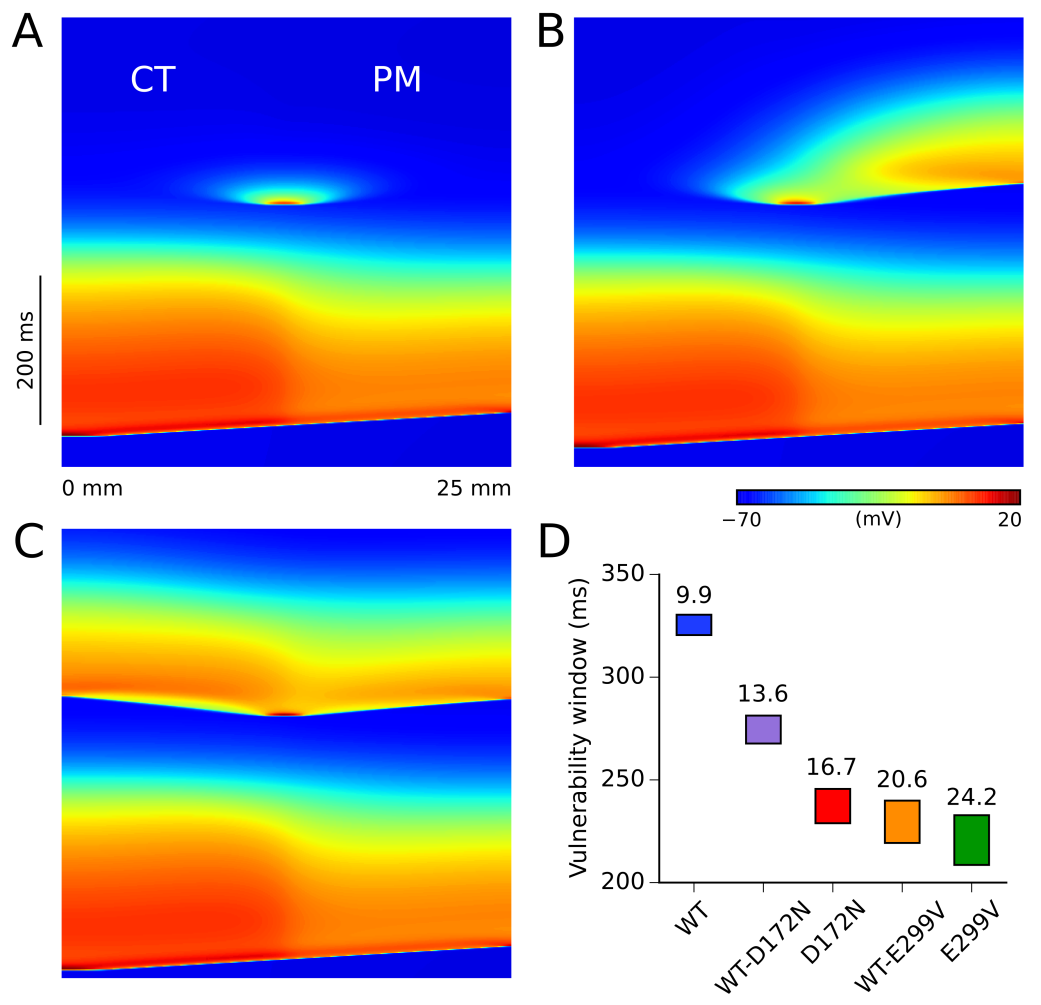


Fig S4. Vulnerability window to uni-directional conduction block. Space-time plots of AP propagation in 1D models of the CT/PM junction used to compute temporal vulnerability to re-entry and corresponding vulnerability window (VW) widths. Three scenarios are shown which correspond to different S2 timings: bi-directional conduction block (A), uni-directional conduction (B), and bi-directional conduction (C). A summary of VW measurements in WT and SQT3 mutation conditions (D).

**2D simulations**

In 2D simulations, the reaction-diffusion equation given in Equation S3 is extended in the *y* direction to give:

$\frac{\partial V}{\partial t}=-\frac{I_{\text{ion}}+I_{\text{stim}}}{C_{\text{m}}}+D\left( \frac{\partial^{2}V}{\partial x^{2}}+\frac{\partial^{2}V}{\partial y^{2}} \right)$ (S4).

Initial conditions in the 2D model, which was 100 × 100 mm^2^, were again taken from steady-state values obtained in a single cell environment. Following initiation of spiral waves in the 2D sheet using an S1-S2 cross-field stimulation protocol, spiral wave trajectories were traced [34], as shown in Fig S5. A summary of the average lifespan and area of meander over time in all mutation conditions is given in Fig S6. The dominant frequencies (DF) as computed in representative 2D simulations (*t* = ERP+20) are summarised in Table S3.


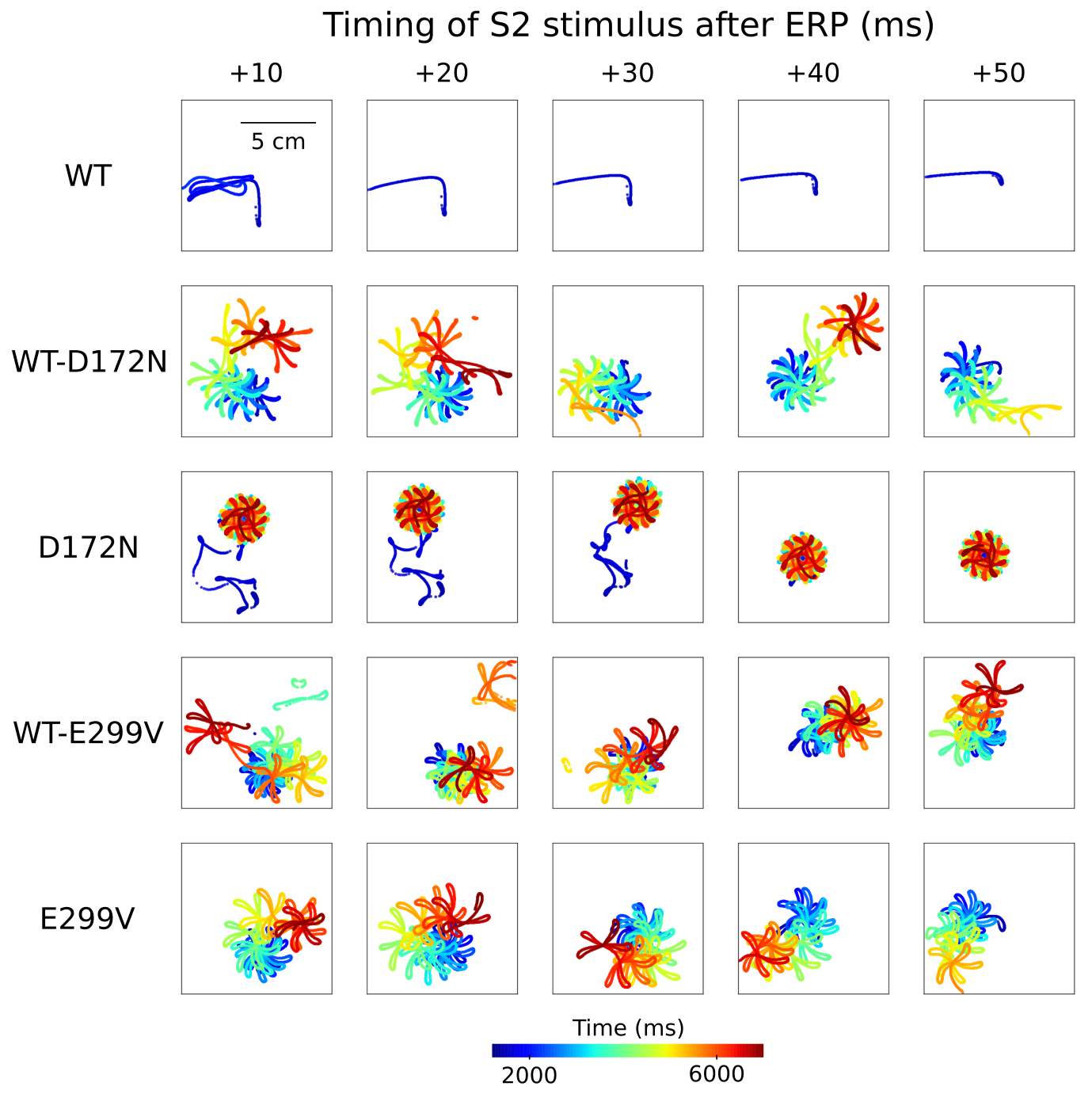


Fig S5. Re-entry simulations in idealised 2D sheet. A summary of rotor trajectories in 2D re-entry simulations for different S2 timings after the effective refractory period (ERP).


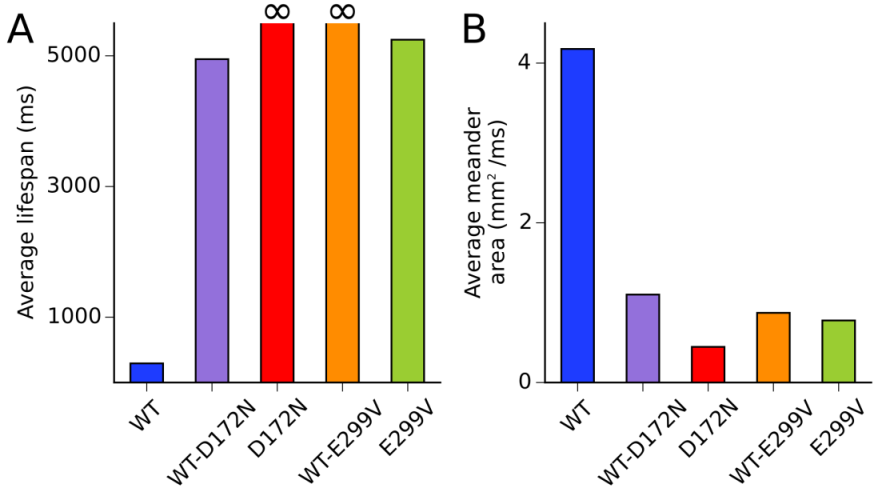


Fig S6. Spiral wave characteristics in 2D re-entry simulations. (A) Bar charts showing the average lifespan of re-entrant excitations in 5 re-entry simulations corresponding to 5 different S2 timings; and (B) the average area of meander over time.

Table S3. Dominant frequency in 2D re-entry simulations.

|  | **WT** | **WT-D172N** | **D172N** | **WT-E299V** | **E299V** |
| --- | --- | --- | --- | --- | --- |
| **DF (Hz)** | N/A | 5.69 | 7.20 | 5.60 | 5.20 |

A summary of dominant frequencies (DF) in SQT3 mutation conditions in a representative 2D spiral wave re-entry simulation.

**3D anatomical human atria geometry simulations**

In anisotropic excitable media such as the 3D human anatomical atria geometry employed in this study [11], the monodomain equation is given by:

$\frac{\partial V}{\partial t}=\nabla\cdot\left( \boldsymbol{D}\nabla V \right)-\left( \frac{I_{\text{ion}}+I_{\text{stim}}}{C_{\text{m}}} \right)$ (S5),

where ∇ is the gradient operator, ***D*** is the diffusion coefficient tensor, and all other parameters retain their previous definitions. In the absence of sheet structure, there are two principal components of ***D***, which describe propagation of APs – along fibres and transverse to the fibre direction, given by $D_{\perp}$ and $D_{\parallel}$, respectively. If ***A*** is a matrix of unit vectors describing the fibre direction, the diffusion coefficient tensor is given by

$\boldsymbol{D}=D_{\perp}\boldsymbol{I}+\left( D_{\parallel}-D_{\perp} \right)\boldsymbol{A}\boldsymbol{A}^{\boldsymbol{T}}$ (S6),

where ***I*** represents the identity matrix, and ***A^T^*** is the transpose of ***A*** [35].

In order to measure activation time (AT) and APD dispersion, the 3D model was paced at 1 Hz from the SAN region. AT was defined as the time at which the membrane potential exceeded −20 mV, and measurement of the APD used a threshold potential as described earlier in the text. Pharmacological modulation of SQT3 mutant tissue was simulated by decreasing or increasing maximal ionic conductances in the AP model. A summary of ΔAPD in the 3D human atria geometry under simulated drug effect conditions is shown in Fig S7. Bar charts showing relative changes to APD and ΔAPD are shown in Fig S8.


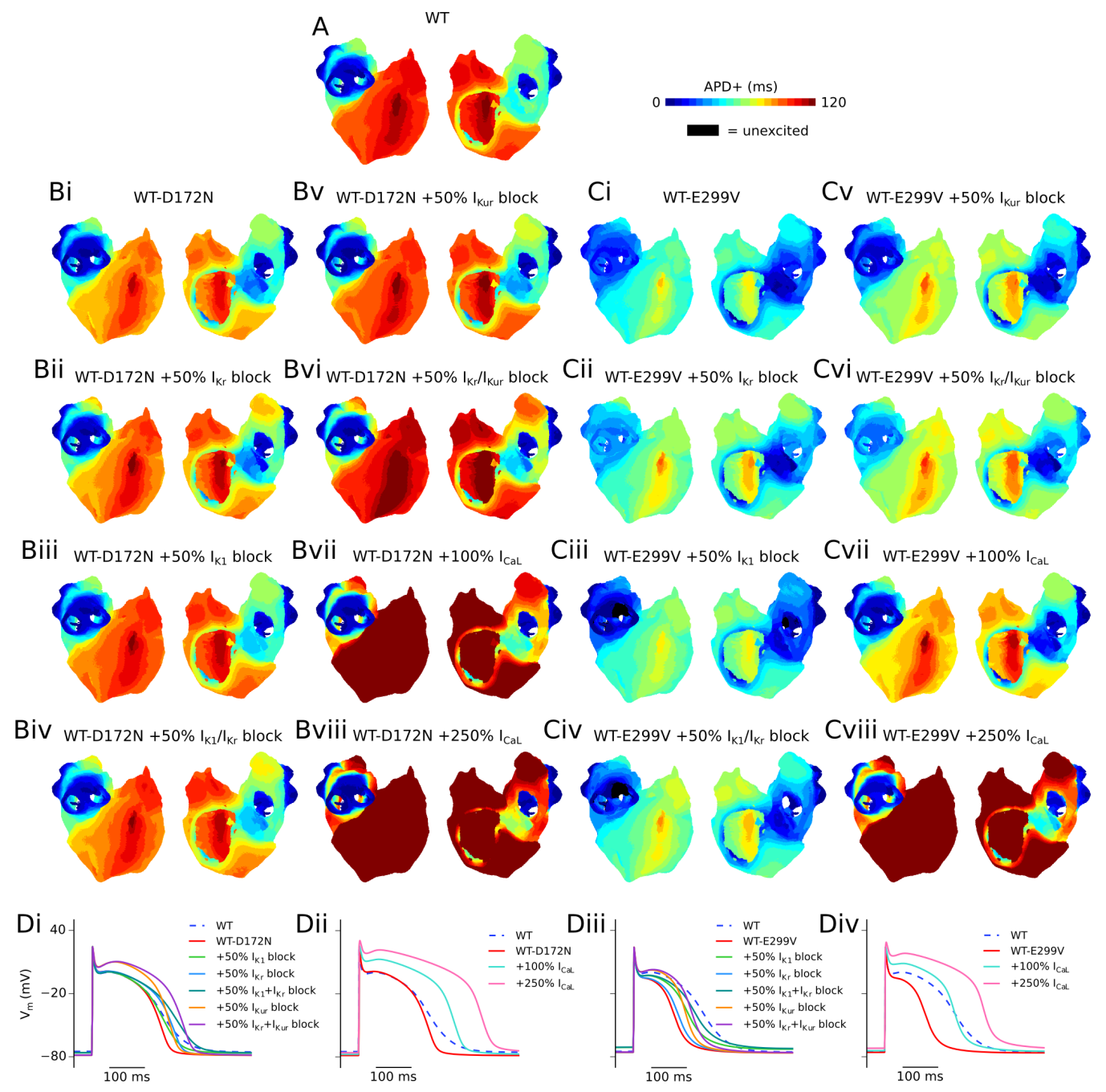


Fig S7. APD dispersion in 3D geometry under simulated pharmacological modulation conditions. (A) WT ΔAPD is shown for reference. ΔAPD in WT-D172N (B) and WT-E299V (C) tissue is shown under the following conditions: (i) control, (ii) 50% I_Kr_ block, (iii) 50% I_K1_ block, (iv) 50% I_K1_+I_Kr_ block, (v) 50% I_Kur_ block, (vi) 50% I_Kr_+I_Kur_ block, (vii) 100% increase in I_CaL_, and (viii) 250% increase in I_CaL_. The colour bar shows APD relative to the shortest APD measured in each condition, designated APD+. The scale of the colour bar is fixed at the value of ΔAPD in the WT condition (120 ms). Regions where APs failed to exceed −20 mV are shown in black. Single cell AP traces at 1 Hz pacing are shown in WT-D172N (Di, Dii) and WT-E299V (Diii, Div) conditions.


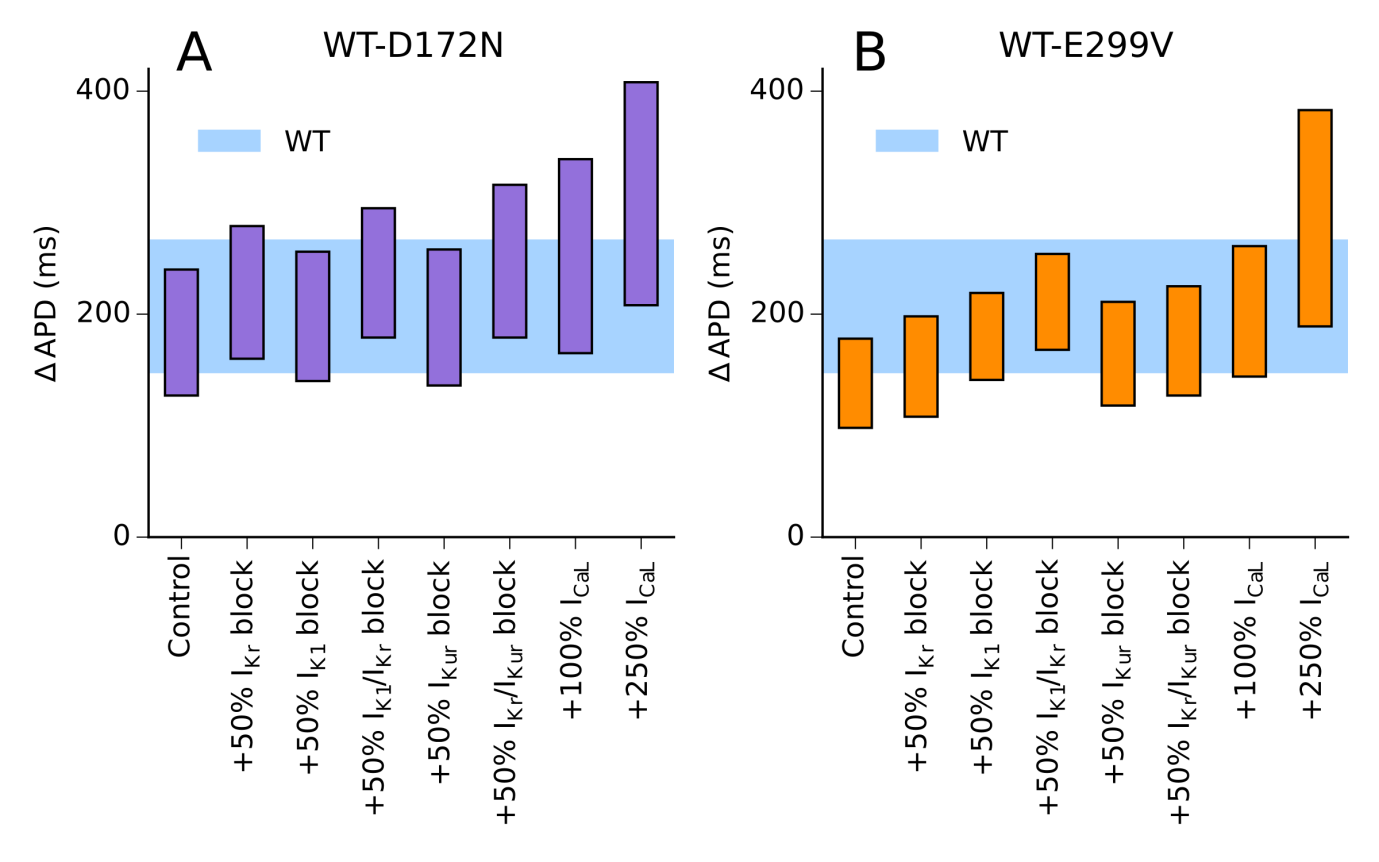


Fig S8. Summary of ΔAPD and relative APD prolongation in pharmacological simulation conditions. Floating bar charts showing ΔAPD in WT-D172N (A) and WT-E299V (B) mutant tissue under various simulated drug effect conditions. The WT ΔAPD is shown in light blue for reference.

Re-entrant excitation waves were initiated in the 3D human atria geometry using the phase distribution method [36], as illustrated in Fig S9. This method has the advantage of not requiring pre-computed values of the ERP which are necessary when using an S1-S2 protocol; thus, if one is interested only in characterising re-entrant wave dynamics and not mechanisms of re-entry initiation, the phase distribution method offers a more efficient simulation method.


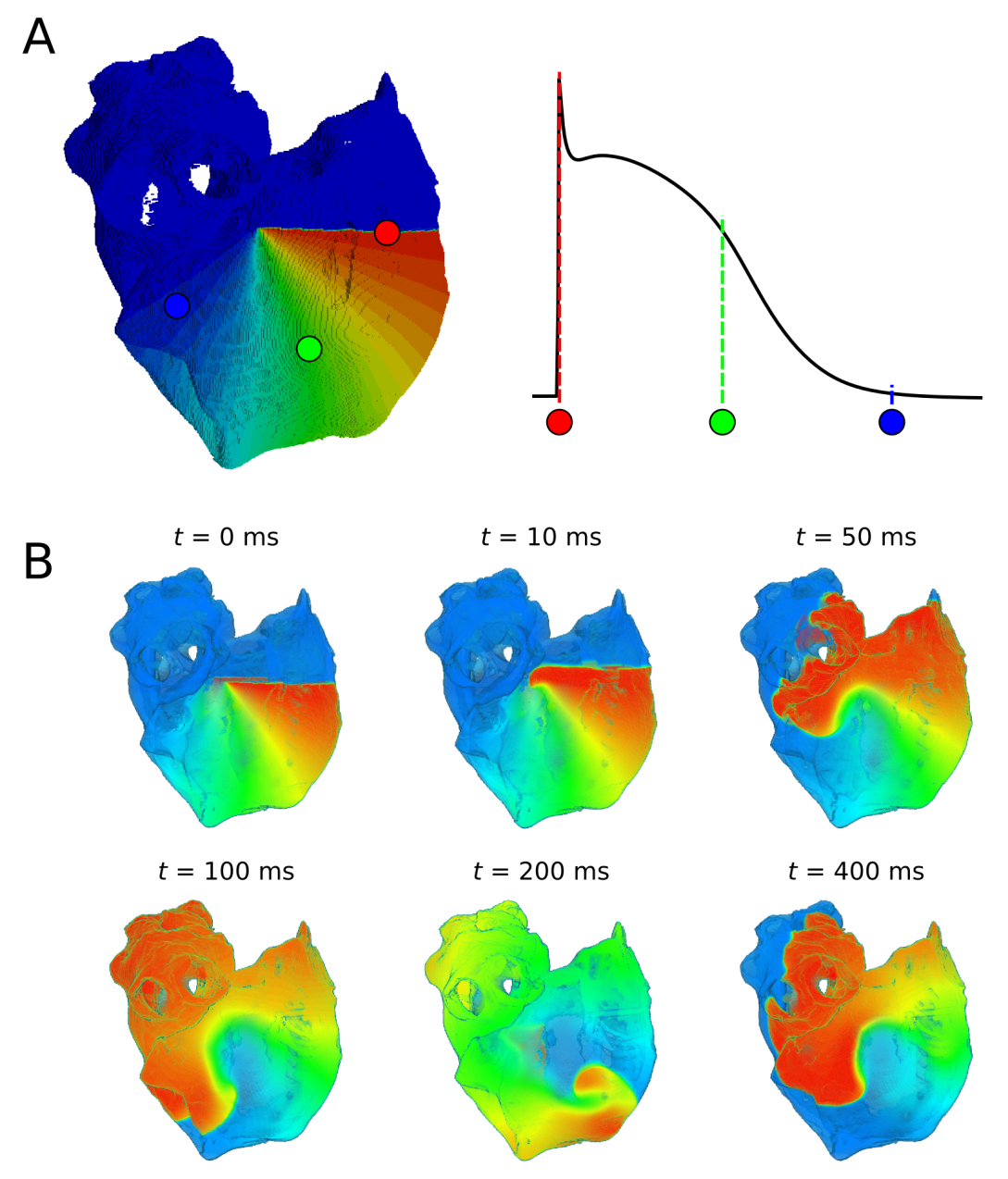


Fig S9. Illustration of the phase distribution method and evolution of scroll waves. (A) Mapping of the membrane potential from an action potential onto the realistic 3D human atria geometry. (B) Evolution of scroll waves in the RA at different time points using phase distribution method initial conditions.

**Comparison of cellular electrophysiological consequences of SQT3 mutations with an alternative human atrial cell model**

Comparative simulations were carried out using the human atrial cell model of Grandi *et al*. [37] (GB model). The baseline GB model was modified in two ways; (i) the fast sodium current (I_Na_) formulation was replaced with that of the CRN human atrial cell model [9] in order to facilitate AP propagation in tissue, and (ii) the calcium handling was simplified as in Chang *et al*. [38] in order to make the model more computationally efficient for tissue simulations, whilst preserving the morphology of the Ca^2+^ transient. Action potentials were visually indistinguishable from those obtained using the published cell model [37] at a pacing rate of 1 Hz.

The WT formulation of I_K1_ was incorporated into the GB model, giving AP properties such as APD_90_, APA, MUV, and RMP which fell within the experimental range for human atrial myocytes. The effects of SQT3 mutations on the AP and I_K1_ profile in the GB model at a pacing frequency of 1 Hz are shown in Fig S10A. Computed APD restitution curves are shown in Fig S10B, along with the maximum slope of restitution. A summary of effects of SQT3 mutations in the GB model at 1 Hz is given in Table S4.


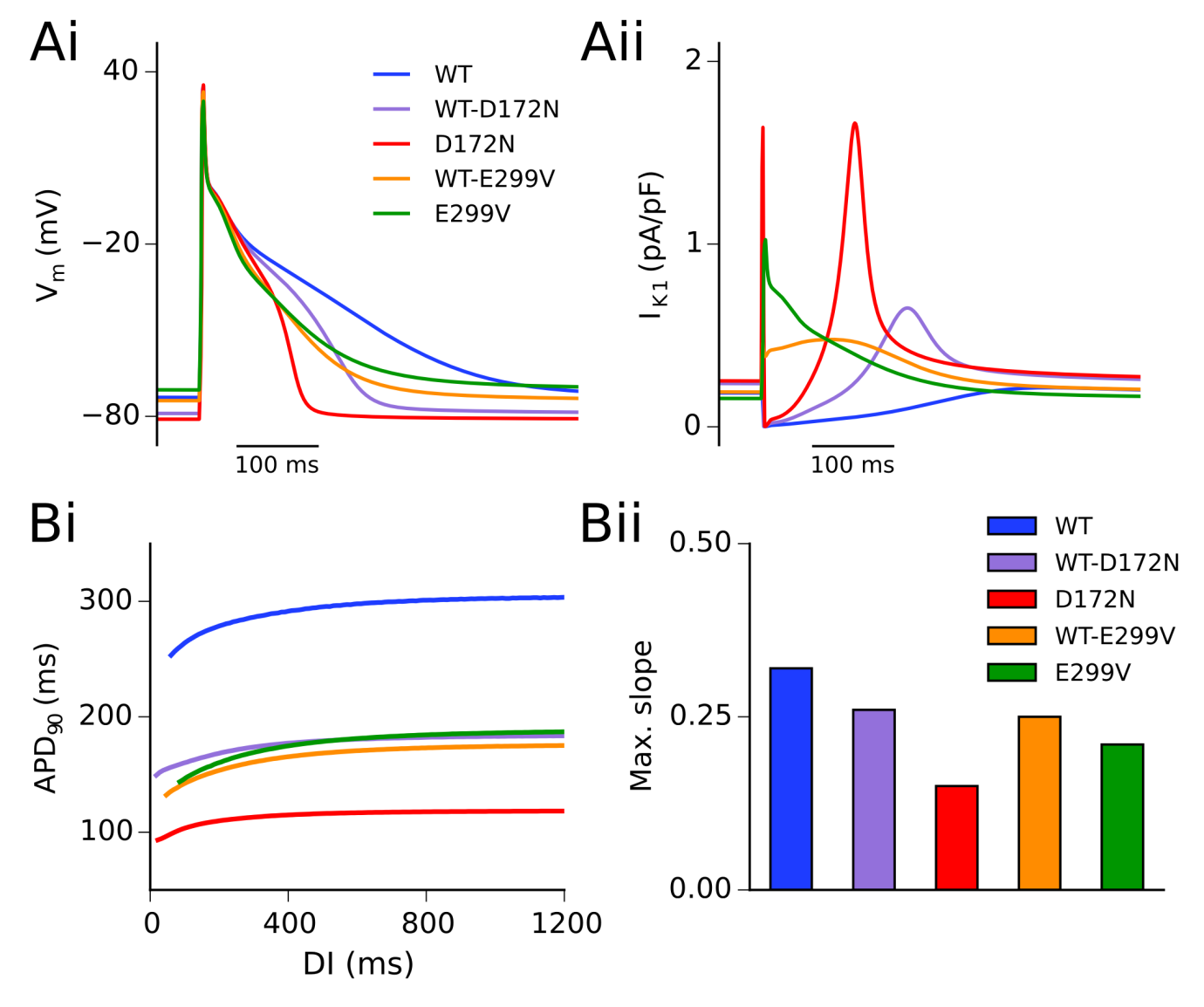


**Fig S10. AP and I_K1_ profile in WT and mutation conditions using the GB model.** Action potential waveforms in WT, WT-D172N, D172N, WT-E299V, and E299V conditions at a pacing frequency of 1 Hz (Ai), with corresponding current trace for I_K1_ (Aii). Restitution of the APD_90_ (Bi), and maximal slope of restitution (Bii).

Table S4. AP properties in WT and SQT3 mutant conditions in the GB model.

|  | **APA (mV)** | **RMP (mV)** | **APD_50_ (ms)** | **APD_90_ (ms)** | **MUV (V/s)** |
| --- | --- | --- | --- | --- | --- |
| **WT** | 106.6 | -73.4 | 59.5 | 299.6 | 168.0 |
| **WT-D172N** | 114.3 | -79.0 | 59.8 | 182.4 | 197.9 |
| **D172N** | 116.5 | -81.0 | 57.3 | 117.9 | 206.3 |
| **WT-E299V** | 107.6 | -74.5 | 46.5 | 173.3 | 174.4 |
| **E299V** | 100.5 | -70.8 | 42.6 | 184.6 | 134.9 |

A summary of AP properties such as action potential amplitude (APA), resting membrane potential (RMP), action potential duration at 50% and 90% repolarisation (APD_50_ and APD_90_, respectively), and maximum upstroke velocity (MUV) in the GB model in WT and SQT3 mutation conditions at a pacing frequency of 1 Hz.

The GB model was extended to incorporate a family of regional cell models, with the baseline AP assumed to be that of a RA cell and changes in maximal ionic conductances representing electrical heterogeneity relative to this implemented as in the CZ model. It was necessary to make the following minor changes in implementation of regional cell models compared to the CZ model. (1) The CT and BB regions included a 35% increase in G_to_ as in [11,39] in order to prevent formation of early after-depolarisations. (2) The PV model used the following slightly modified conductance scaling and kinetic factors (which were close to experimental data) compared to the LA model: G_Kr_ ×2.0, G_Ks_ ×2.0, G_CaL_ ×0.6, G_K1_ ×0.8, G_to_ ×0.85, V_1/2_ I_K1_ −5 mV in order to give close agreement with experimentally-measured differences in APD_90_, MUV, APA, and RMP [27–29]. Regional cell model action potentials in the GB model are shown in Fig S11A. Global ΔAPD in the 3D human atria, as measured using the previously-described protocol, is summarised in Figs S11B and S11C.


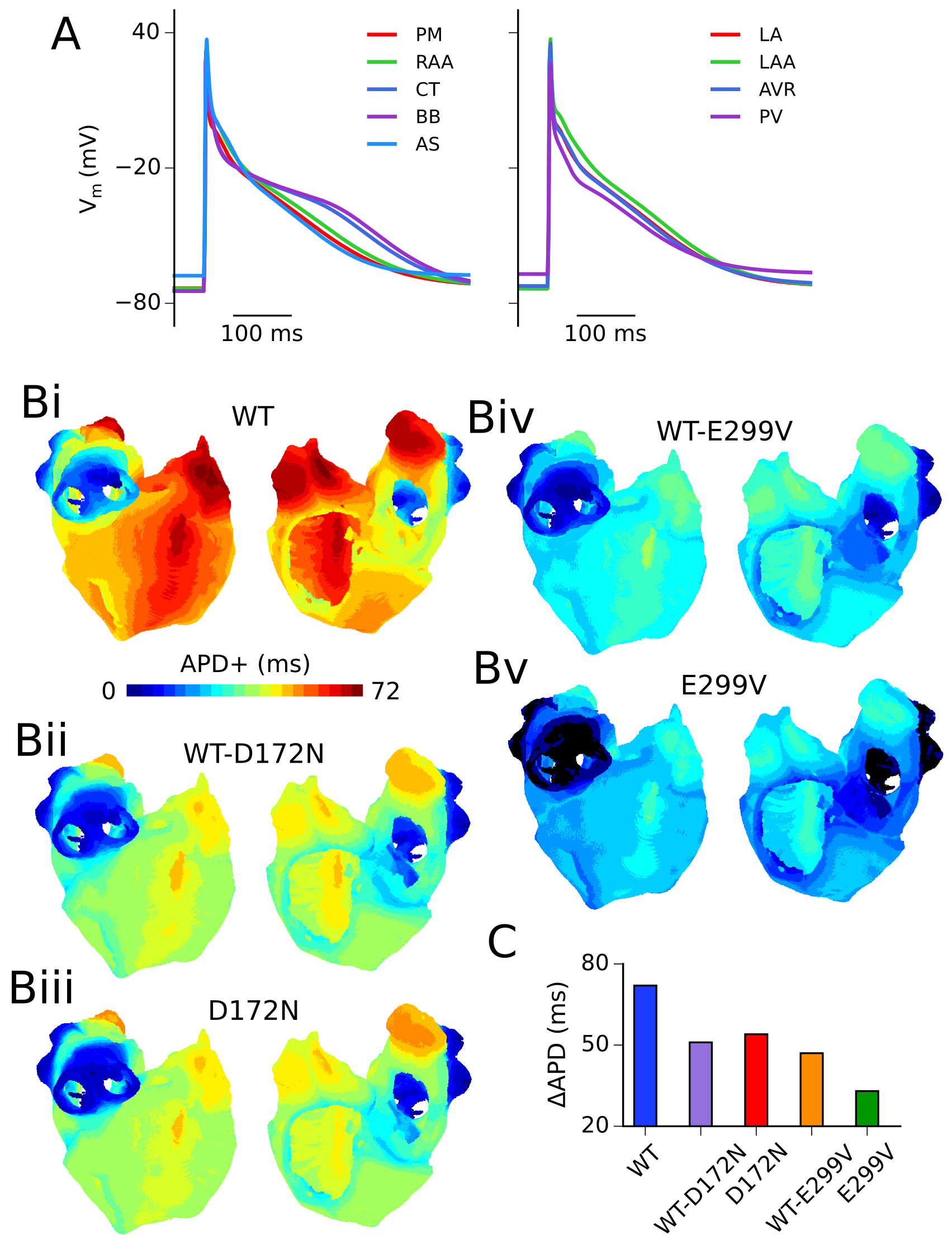


**Fig S11. Regional cell models and spatial dispersion of APD in GB model.** Regional cell models in the GB model (A), including PM, RAA, CT, BB, AS, LA, LAA, AVR, PV. APD distribution maps in WT (Bi), WT-D172N (Bii), D172N (Biii), WT-E299V (Biv), and E299V (Bv) mutation conditions, with corresponding ΔAPD (C). The colour bar shows APD relative to the shortest APD measured in each condition, designated APD+. The scale of the colour bar is fixed at the value of ΔAPD in the WT condition (72 ms). The colour black shows regions where membrane potentials failed to exceed a threshold value (−20 mV).

**Comparison of I_K1_ formulation with previous studies**

I_K1_ current density has been reported to be smaller in human atria than ventricles [10]. Comparison of WT I_K1_ used in the present study and the WT formulation in our previous study on the D172N Kir2.1 mutations in human ventricles [8] showed that differences in absolute magnitude closely matched those observed between human atrial and ventricular mycoytes [10], as shown in Fig S12A. WT I_K1_ in the present study also showed a high degree of similarity in I-V relationship with the baseline I_K1_ formulations used in other leading human atrial cell models; namely the models of Grandi *et al*. [37], Courtemanche *et al*. [9], and Nygren *et al*. [40]. (see Fig S12B).


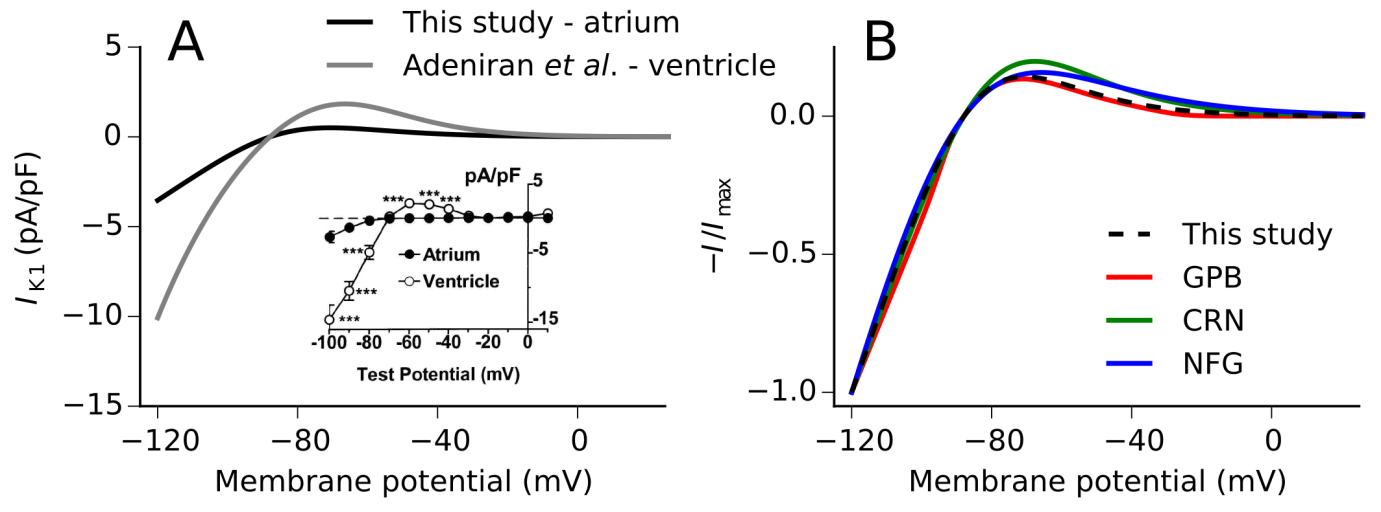


Fig S12. Comparison of I_K1_ kinetics with previous studies. (A) Simulated I-V relationships for the WT I_K1_ formulation used in the human atrial cell model in the present study compared with the WT I_K1_ formulation used in our previous study in human ventricular cells [8] – the inset shows native I_K1_ recordings in human atrial and ventricular myocytes taken from [10]. (B) Simulated I-V relationships for I_K1_ used in this study compared with I_K1_ from the Grandi *et al*. (GPB) model [37], Courtemanche *et al*. (CRN) model [9], and the Nygren *et al*. (NFG) model [40].

1. Kharche S, Garratt CJ, Boyett MR, Inada S, Holden AV, Hancox JC, et al. Atrial proarrhythmia due to increased inward rectifier current (IK1) arising from KCNJ2 mutation – A simulation study. Prog Biophys Mol Biol. 2008;98: 186–197. doi:10.1016/j.pbiomolbio.2008.10.010

2. Sakmann B, Trube G. Conductance properties of single inwardly rectifying potassium channels in ventricular cells from guinea-pig heart. J Physiol. 1984;347: 641–657. doi:10.1113/jphysiol.1984.sp015088

3. Bailly P, Mouchonière M, Bénitah J-P, Camilleri L, Vassort G, Lorente P. Extracellular K+ Dependence of Inward Rectification Kinetics in Human Left Ventricular Cardiomyocytes. Circulation. 1998;98: 2753–2759. doi:10.1161/01.CIR.98.24.2753

4. Dhamoon AS, Jalife J. The inward rectifier current (IK1) controls cardiac excitability and is involved in arrhythmogenesis. Heart Rhythm. 2005;2: 316–324. doi:10.1016/j.hrthm.2004.11.012

5. El Harchi A, McPate MJ, Zhang Y hong, Zhang H, Hancox JC. Action potential clamp and chloroquine sensitivity of mutant Kir2.1 channels responsible for variant 3 short QT syndrome. J Mol Cell Cardiol. 2009;47: 743–747. doi:10.1016/j.yjmcc.2009.02.027

6. Deo M, Ruan Y, Pandit SV, Shah K, Berenfeld O, Blaufox A, et al. KCNJ2 mutation in short QT syndrome 3 results in atrial fibrillation and ventricular proarrhythmia. Proc Natl Acad Sci. 2013;110: 4291–4296. doi:10.1073/pnas.1218154110

7. Nelder JA, Mead R. A Simplex Method for Function Minimization. Comput J. 1965;7: 308–313. doi:10.1093/comjnl/7.4.308

8. Adeniran I, Harchi AE, Hancox JC, Zhang H. Proarrhythmia in KCNJ2-linked short QT syndrome: insights from modelling. Cardiovasc Res. 2012;94: 66–76. doi:10.1093/cvr/cvs082

9. Courtemanche M, Ramirez RJ, Nattel S. Ionic mechanisms underlying human atrial action potential properties: insights from a mathematical model. Am J Physiol - Heart Circ Physiol. 1998;275: H301–H321.

10. Wang Z, Yue L, White M, Pelletier G, Nattel S. Differential Distribution of Inward Rectifier Potassium Channel Transcripts in Human Atrium Versus Ventricle. Circulation. 1998;98: 2422–2428. doi:10.1161/01.CIR.98.22.2422

11. Colman MA, Aslanidi OV, Kharche S, Boyett MR, Garratt C, Hancox JC, et al. Pro-arrhythmogenic effects of atrial fibrillation-induced electrical remodelling: insights from the three-dimensional virtual human atria. J Physiol. 2013;591: 4249–4272. doi:10.1113/jphysiol.2013.254987

12. Colman MA, Ni H, Liang B, Schmitt N, Zhang H. In silico assessment of genetic variation in KCNA5 reveals multiple mechanisms of atrial arrhythmogenesis. 2017; PCOMPBIOL-D-17-00219.

13. Gong D, Zhang Y, Cai B, Meng Q, Jiang S, Li X, et al. Characterization and comparison of Na+, K+ and Ca2+ currents between myocytes from human atrial right appendage and atrial septum. Cell Physiol Biochem Int J Exp Cell Physiol Biochem Pharmacol. 2008;21: 385–394. doi:10.1159/000129631

14. Poulet C, Wettwer E, Grunnet M, Jespersen T, Fabritz L, Matschke K, et al. Late Sodium Current in Human Atrial Cardiomyocytes from Patients in Sinus Rhythm and Atrial Fibrillation. PLoS ONE. 2015;10. doi:10.1371/journal.pone.0131432

15. Hordof AJ, Edie R, Malm JR, Hoffman BF, Rosen MR. Electrophysiologic properties and response to pharmacologic agents of fibers from diseased human atria. Circulation. 1976;54: 774–779. doi:10.1161/01.CIR.54.5.774

16. Gelband H, Bush HL, Rosen MR, Myerburg RJ, Hoffman BF. Electrophysiologic Properties of Isolated Preparations of Human Atrial Myocardium. Circ Res. 1972;30: 293–300. doi:10.1161/01.RES.30.3.293

17. Pau D, Workman AJ, Kane KA, Rankin AC. Electrophysiological and arrhythmogenic effects of 5-hydroxytryptamine on human atrial cells are reduced in atrial fibrillation. J Mol Cell Cardiol. 2007;42: 54–62. doi:10.1016/j.yjmcc.2006.08.007

18. Calum J Redpath ACR. Anti-adrenergic effects of endothelin on human atrial action potentials are potentially anti-arrhythmic. J Mol Cell Cardiol. 2006;40: 717–24. doi:10.1016/j.yjmcc.2006.01.012

19. Katoh H, Shinozaki T, Baba S, Satoh S, Kagaya Y, Watanabe J, et al. Monophasic Action Potential Duration at the Crista Terminalis in Patients With Sinus Node Disease. Circ J. 2005;69: 1361–1367. doi:10.1253/circj.69.1361

20. Bosch RF, Zeng X, Grammer JB, Popovic K, Mewis C, Kühlkamp V. Ionic mechanisms of electrical remodeling in human atrial fibrillation. Cardiovasc Res. 1999;44: 121–131. doi:10.1016/S0008-6363(99)00178-9

21. Dobrev D, Ravens U. Remodeling of cardiomyocyte ion channels in human atrial fibrillation. Basic Res Cardiol. 2003;98: 137–148. doi:10.1007/s00395-003-0409-8

22. Kim B-S, Kim Y-H, Hwang G-S, Pak H-N, Lee SC, Shim WJ, et al. Action potential duration restitution kinetics in human atrial fibrillation. J Am Coll Cardiol. 2002;39: 1329–1336. doi:10.1016/S0735-1097(02)01760-6

23. Caballero R, de la Fuente MG, Gómez R, Barana A, Amorós I, Dolz-Gaitón P, et al. In Humans, Chronic Atrial Fibrillation Decreases the Transient Outward Current and Ultrarapid Component of the Delayed Rectifier Current Differentially on Each Atria and Increases the Slow Component of the Delayed Rectifier Current in Both. J Am Coll Cardiol. 2010;55: 2346–2354. doi:10.1016/j.jacc.2010.02.028

24. Feng J, Yue L, Wang Z, Nattel S. Ionic Mechanisms of Regional Action Potential Heterogeneity in the Canine Right Atrium. Circ Res. 1998;83: 541–551. doi:10.1161/01.RES.83.5.541

25. Burashnikov A, Mannava S, Antzelevitch C. Transmembrane action potential heterogeneity in the canine isolated arterially perfused right atrium: effect of IKr and IKur/Ito block. Am J Physiol - Heart Circ Physiol. 2004;286: H2393–H2400. doi:10.1152/ajpheart.01242.2003

26. Li D, Zhang L, Kneller J, Nattel S. Potential Ionic Mechanism for Repolarization Differences Between Canine Right and Left Atrium. Circ Res. 2001;88: 1168–1175. doi:10.1161/hh1101.091266

27. Ehrlich JR, Cha T-J, Zhang L, Chartier D, Melnyk P, Hohnloser SH, et al. Cellular electrophysiology of canine pulmonary vein cardiomyocytes: action potential and ionic current properties. J Physiol. 2003;551: 801–813. doi:10.1113/jphysiol.2003.046417

28. Datino T, Macle L, Qi X-Y, Maguy A, Comtois P, Chartier D, et al. Mechanisms by Which Adenosine Restores Conduction in Dormant Canine Pulmonary Veins. Circulation. 2010;121: 963–972. doi:10.1161/CIRCULATIONAHA.109.893107

29. Cha T-J, Ehrlich JR, Zhang L, Chartier D, Leung TK, Nattel S. Atrial Tachycardia Remodeling of Pulmonary Vein Cardiomyocytes. Circulation. 2005;111: 728–735. doi:10.1161/01.CIR.0000155240.05251.D0

30. Wang Z, Fermini B, Nattel S. Delayed rectifier outward current and repolarization in human atrial myocytes. Circ Res. 1993;73: 276–285. doi:10.1161/01.RES.73.2.276

31. Wettwer E, Hála O, Christ T, Heubach JF, Dobrev D, Knaut M, et al. Role of IKur in Controlling Action Potential Shape and Contractility in the Human Atrium. Circulation. 2004;110: 2299–2306. doi:10.1161/01.CIR.0000145155.60288.71

32. Koumi S, Backer CL, Arentzen CE. Characterization of Inwardly Rectifying K+ Channel in Human Cardiac Myocytes. Circulation. 1995;92: 164–174. doi:10.1161/01.CIR.92.2.164

33. Workman AJ, Kane KA, Rankin AC. The contribution of ionic currents to changes in refractoriness of human atrial myocytes associated with chronic atrial fibrillation. Cardiovasc Res. 2001;52: 226–235. doi:10.1016/S0008-6363(01)00380-7

34. Bray M-A, Wikswo JP. Use of topological charge to determine filament location and dynamics in a numerical model of scroll wave activity. IEEE Trans Biomed Eng. 2002;49: 1086–1093. doi:10.1109/TBME.2002.803516

35. Clayton RH, Panfilov AV. A guide to modelling cardiac electrical activity in anatomically detailed ventricles. Prog Biophys Mol Biol. 2008;96: 19–43. doi:10.1016/j.pbiomolbio.2007.07.004

36. Biktashev VN, Holden AV. Reentrant waves and their elimination in a model of mammalian ventricular tissue. Chaos Interdiscip J Nonlinear Sci. 1998;8: 48–56. doi:10.1063/1.166307

37. Grandi E, Pandit SV, Voigt N, Workman AJ, Dobrev D, Jalife J, et al. Human Atrial Action Potential and Ca2+ Model: Sinus Rhythm and Chronic Atrial Fibrillation. Circ Res. 2011;109: 1055–1066. doi:10.1161/CIRCRESAHA.111.253955

38. Chang KC, Bayer JD, Trayanova NA. Disrupted Calcium Release as a Mechanism for Atrial Alternans Associated with Human Atrial Fibrillation. PLOS Comput Biol. 2014;10: e1004011. doi:10.1371/journal.pcbi.1004011

39. Seemann G, Höper C, Sachse FB, Dössel O, Holden AV, Zhang H. Heterogeneous three-dimensional anatomical and electrophysiological model of human atria. Philos Trans R Soc Lond Math Phys Eng Sci. 2006;364: 1465–1481. doi:10.1098/rsta.2006.1781

40. Nygren A, Fiset C, Firek L, Clark JW, Lindblad DS, Clark RB, et al. Mathematical Model of an Adult Human Atrial Cell The Role of K+ Currents in Repolarization. Circ Res. 1998;82: 63–81. doi:10.1161/01.RES.82.1.63
