# Supplementary material for: Atrial arrhythmogenicity of KCNJ2 mutations in short QT syndrome: Insights from virtual human atria
Source: PLoS Comput Biol. 2017 Jun 13;13(6):e1005593. doi: 10.1371/journal.pcbi.1005593 (PMC5487071; doi:10.1371/journal.pcbi.1005593)
Supplement: S1 Fig — (Ai) A comparison of the baseline right atrium (RA) model AP used in this study with experimental recordings of action potentials taken from human RA myocytes (Bosch et al., 1999; Wang et al., 1993; Wettwer et al., 2004; Koumi et al., 1995; Workman et al., 2001). (Aii) Validation of baseline model against experimentally-measured metrics; action potential amplitude (APA), maximum upstroke velocity (MUV), action potential duration at 50% and 90% (APD50 and APD90, respectively), and resting membrane potential (RMP), using data from Gong et al., 2008; Poulet et al., 2015; Hordof et al., 1976; Gelband et al., 1972; Pau et al., 2007; Redpath et al., 2006; Katoh et al., 2005; Bosch et al., 1999; Dobrev & Ravens, 2003; Kim et al., 2002). (Bi) Regional cell model action potentials from the crista terminalis (CT), right atrial appendage (RAA), atrio-ventricular ring (AVR), atrial septum (AS), Bachmann’s bundle (BB), left atrium (LA), pectinate muscles (PM), and RA. (Bii) Comparison of APD90 ratios (APD95 for Feng et al., 1998) in regional cell models using experimental data from Gong et al., 2008; Katoh et al., 2005; Feng et al., 1998; Burashnikov et al., 2004; Li et al., 2001. (DOCX) [file pcbi.1005593.s002.docx]

**Fig S1**

**Atrial arrhythmogenicity of KCNJ2-linked short QT syndrome mutations: insights from virtual human atria**

Dominic G. Whittaker, Haibo Ni, Aziza El Harchi, Jules C. Hancox, Henggui Zhang


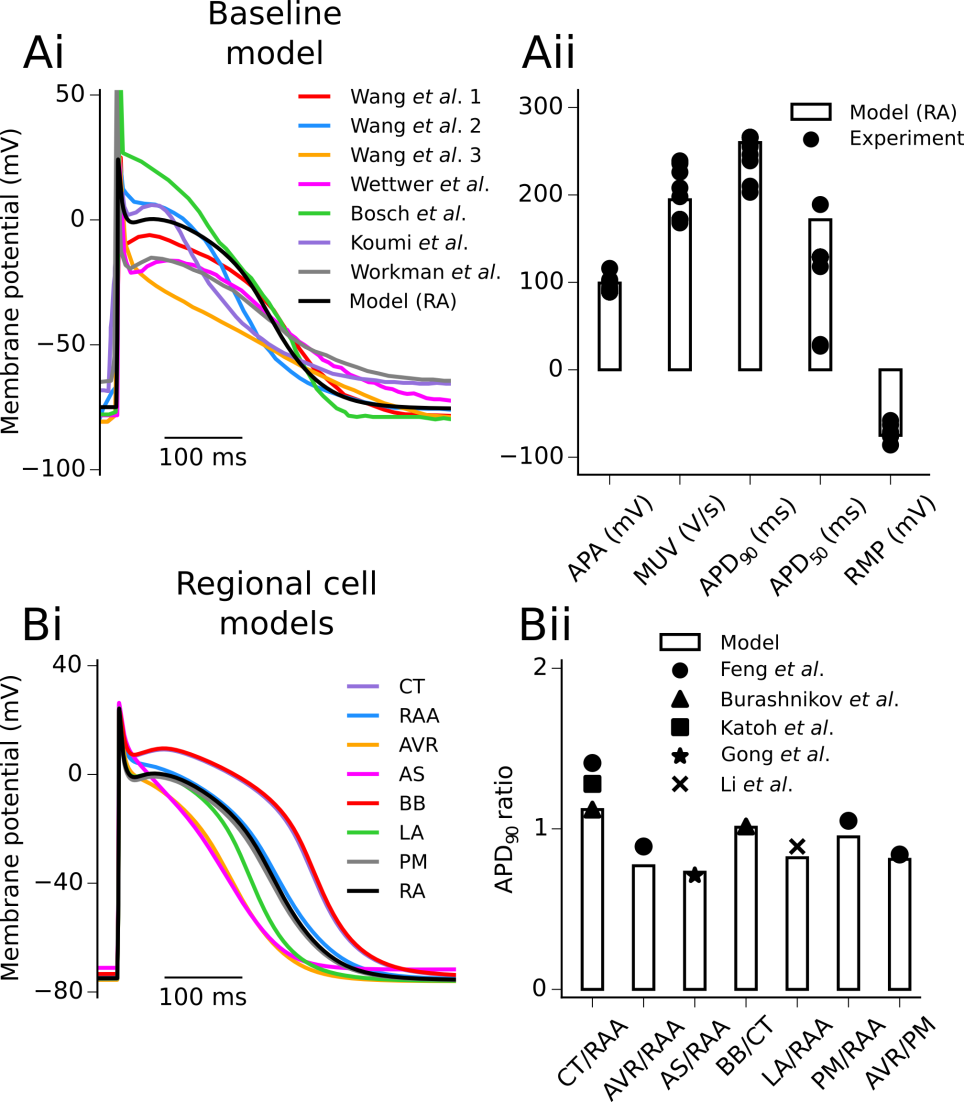


Fig S1. Comparison of baseline and regional cell model APs with experimental data. (Ai) A comparison of the baseline right atrium (RA) model AP used in this study with experimental recordings of action potentials taken from human RA myocytes [1–5]. (Aii) Validation of baseline model against experimentally-measured metrics; action potential amplitude (APA), maximum upstroke velocity (MUV), action potential duration at 50% and 90% (APD_50_ and APD_90_, respectively), and resting membrane potential (RMP), using data from [3,6–14]. (Bi) Regional cell model action potentials from the crista terminalis (CT), right atrial appendage (RAA), atrio-ventricular ring (AVR), atrial septum (AS), Bachmann’s bundle (BB), left atrium (LA), pectinate muscles (PM), and RA. (Bii) Comparison of APD_90_ ratios (APD_95_ for Feng *et al*. [15]) in regional cell models using experimental data from [6,12,15–17].

1. Wang Z, Fermini B, Nattel S. Delayed rectifier outward current and repolarization in human atrial myocytes. Circ Res. 1993;73: 276–285. doi:10.1161/01.RES.73.2.276

2. Wettwer E, Hála O, Christ T, Heubach JF, Dobrev D, Knaut M, et al. Role of IKur in Controlling Action Potential Shape and Contractility in the Human Atrium. Circulation. 2004;110: 2299–2306. doi:10.1161/01.CIR.0000145155.60288.71

3. Bosch RF, Zeng X, Grammer JB, Popovic K, Mewis C, Kühlkamp V. Ionic mechanisms of electrical remodeling in human atrial fibrillation. Cardiovasc Res. 1999;44: 121–131. doi:10.1016/S0008-6363(99)00178-9

4. Koumi S, Backer CL, Arentzen CE. Characterization of Inwardly Rectifying K+ Channel in Human Cardiac Myocytes. Circulation. 1995;92: 164–174. doi:10.1161/01.CIR.92.2.164

5. Workman AJ, Kane KA, Rankin AC. The contribution of ionic currents to changes in refractoriness of human atrial myocytes associated with chronic atrial fibrillation. Cardiovasc Res. 2001;52: 226–235. doi:10.1016/S0008-6363(01)00380-7

6. Gong D, Zhang Y, Cai B, Meng Q, Jiang S, Li X, et al. Characterization and comparison of Na+, K+ and Ca2+ currents between myocytes from human atrial right appendage and atrial septum. Cell Physiol Biochem Int J Exp Cell Physiol Biochem Pharmacol. 2008;21: 385–394. doi:10.1159/000129631

7. Poulet C, Wettwer E, Grunnet M, Jespersen T, Fabritz L, Matschke K, et al. Late Sodium Current in Human Atrial Cardiomyocytes from Patients in Sinus Rhythm and Atrial Fibrillation. PLoS ONE. 2015;10. doi:10.1371/journal.pone.0131432

8. Hordof AJ, Edie R, Malm JR, Hoffman BF, Rosen MR. Electrophysiologic properties and response to pharmacologic agents of fibers from diseased human atria. Circulation. 1976;54: 774–779. doi:10.1161/01.CIR.54.5.774

9. Gelband H, Bush HL, Rosen MR, Myerburg RJ, Hoffman BF. Electrophysiologic Properties of Isolated Preparations of Human Atrial Myocardium. Circ Res. 1972;30: 293–300. doi:10.1161/01.RES.30.3.293

10. Pau D, Workman AJ, Kane KA, Rankin AC. Electrophysiological and arrhythmogenic effects of 5-hydroxytryptamine on human atrial cells are reduced in atrial fibrillation. J Mol Cell Cardiol. 2007;42: 54–62. doi:10.1016/j.yjmcc.2006.08.007

11. Calum J Redpath ACR. Anti-adrenergic effects of endothelin on human atrial action potentials are potentially anti-arrhythmic. J Mol Cell Cardiol. 2006;40: 717–24. doi:10.1016/j.yjmcc.2006.01.012

12. Katoh H, Shinozaki T, Baba S, Satoh S, Kagaya Y, Watanabe J, et al. Monophasic Action Potential Duration at the Crista Terminalis in Patients With Sinus Node Disease. Circ J. 2005;69: 1361–1367. doi:10.1253/circj.69.1361

13. Dobrev D, Ravens U. Remodeling of cardiomyocyte ion channels in human atrial fibrillation. Basic Res Cardiol. 2003;98: 137–148. doi:10.1007/s00395-003-0409-8

14. Kim B-S, Kim Y-H, Hwang G-S, Pak H-N, Lee SC, Shim WJ, et al. Action potential duration restitution kinetics in human atrial fibrillation. J Am Coll Cardiol. 2002;39: 1329–1336. doi:10.1016/S0735-1097(02)01760-6

15. Feng J, Yue L, Wang Z, Nattel S. Ionic Mechanisms of Regional Action Potential Heterogeneity in the Canine Right Atrium. Circ Res. 1998;83: 541–551. doi:10.1161/01.RES.83.5.541

16. Burashnikov A, Mannava S, Antzelevitch C. Transmembrane action potential heterogeneity in the canine isolated arterially perfused right atrium: effect of IKr and IKur/Ito block. Am J Physiol - Heart Circ Physiol. 2004;286: H2393–H2400. doi:10.1152/ajpheart.01242.2003

17. Li D, Zhang L, Kneller J, Nattel S. Potential Ionic Mechanism for Repolarization Differences Between Canine Right and Left Atrium. Circ Res. 2001;88: 1168–1175. doi:10.1161/hh1101.091266
