# Supplementary material for: Atrial arrhythmogenicity of KCNJ2 mutations in short QT syndrome: Insights from virtual human atria
Source: PLoS Comput Biol. 2017 Jun 13;13(6):e1005593. doi: 10.1371/journal.pcbi.1005593 (PMC5487071; doi:10.1371/journal.pcbi.1005593)
Supplement: S2 Fig — (A) A comparison of model changes in maximal ionic conductances of Ito, IKs, IKr, IK1, and ICaL between PV and LA models against experimental measurements. (B) Comparison of AP characteristics, namely APD90, MUV, and APA, between PV and LA models against experimental data. (C) Action potentials from the LA and PV models at a pacing frequency of 2 Hz compared with experimentally-recorded APs from canine atrial myocytes (Cha et al., 2005) shown inset. (D) Comparison of difference in RMP between LA and PV models against experimental measurements. All experimental data are taken from Ehrlich et al., 2003; Datino et al., 2010; Cha et al., 2005). (DOCX) [file pcbi.1005593.s003.docx]

**Fig S2**

**Atrial arrhythmogenicity of KCNJ2-linked short QT syndrome mutations: insights from virtual human atria**

Dominic G. Whittaker, Haibo Ni, Aziza El Harchi, Jules C. Hancox, Henggui Zhang


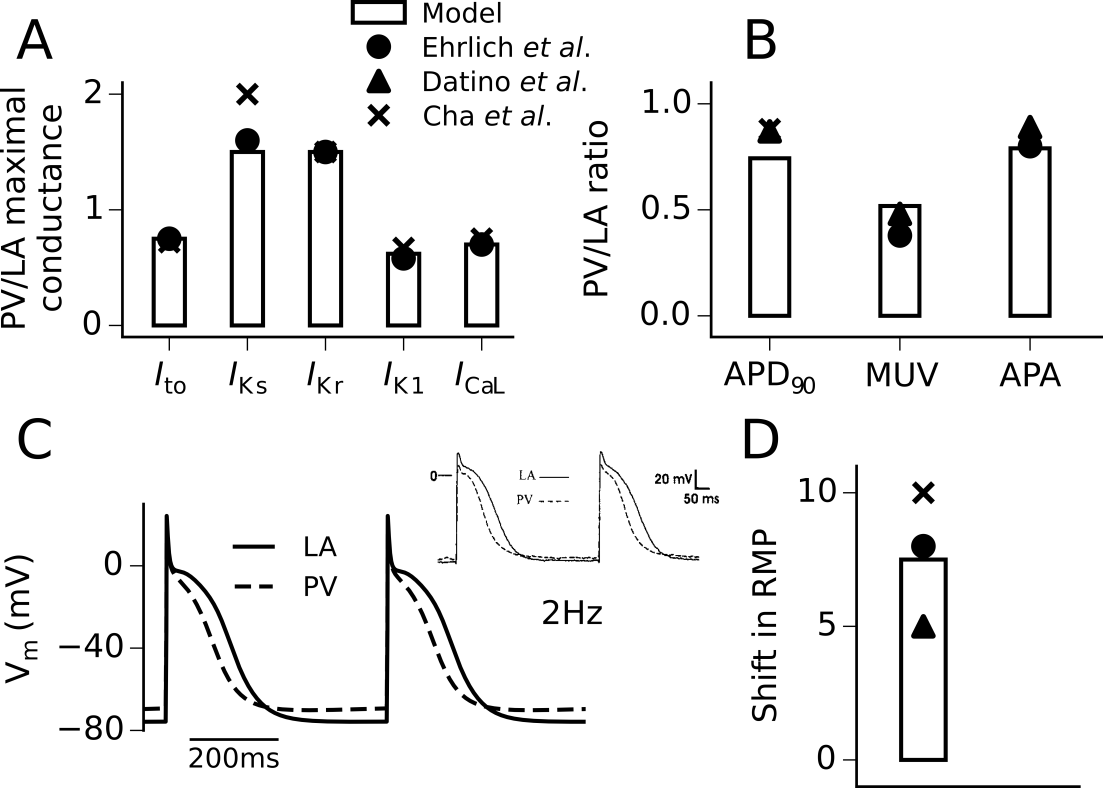


Fig S2. Validation of the pulmonary vein model. (A) A comparison of model changes in maximal ionic conductances of I_to_, I_Ks_, I_Kr_, I_K1_, and I_CaL_ between PV and LA models against experimental measurements. (B) Comparison of AP characteristics, namely APD_90_, MUV, and APA, between PV and LA models against experimental data. (C) Action potentials from the LA and PV models at a pacing frequency of 2 Hz compared with experimentally-recorded APs from canine atrial myocytes [1] shown inset. (D) Comparison of difference in RMP between LA and PV models against experimental measurements. All experimental data are taken from [1–3].

1. Cha T-J, Ehrlich JR, Zhang L, Chartier D, Leung TK, Nattel S. Atrial Tachycardia Remodeling of Pulmonary Vein Cardiomyocytes. Circulation. 2005;111: 728–735. doi:10.1161/01.CIR.0000155240.05251.D0

2. Ehrlich JR, Cha T-J, Zhang L, Chartier D, Melnyk P, Hohnloser SH, et al. Cellular electrophysiology of canine pulmonary vein cardiomyocytes: action potential and ionic current properties. J Physiol. 2003;551: 801–813. doi:10.1113/jphysiol.2003.046417

3. Datino T, Macle L, Qi X-Y, Maguy A, Comtois P, Chartier D, et al. Mechanisms by Which Adenosine Restores Conduction in Dormant Canine Pulmonary Veins. Circulation. 2010;121: 963–972. doi:10.1161/CIRCULATIONAHA.109.893107
