# Supplementary material for: Atrial arrhythmogenicity of KCNJ2 mutations in short QT syndrome: Insights from virtual human atria
Source: PLoS Comput Biol. 2017 Jun 13;13(6):e1005593. doi: 10.1371/journal.pcbi.1005593 (PMC5487071; doi:10.1371/journal.pcbi.1005593)
Supplement: S3 Fig — ΔAPD at the CT/PM and PV/LA junctions as determined in 1D tissue models. (DOCX) [file pcbi.1005593.s004.docx]

**Fig S3**

**Atrial arrhythmogenicity of KCNJ2-linked short QT syndrome mutations: insights from virtual human atria**

Dominic G. Whittaker, Haibo Ni, Aziza El Harchi, Jules C. Hancox, Henggui Zhang


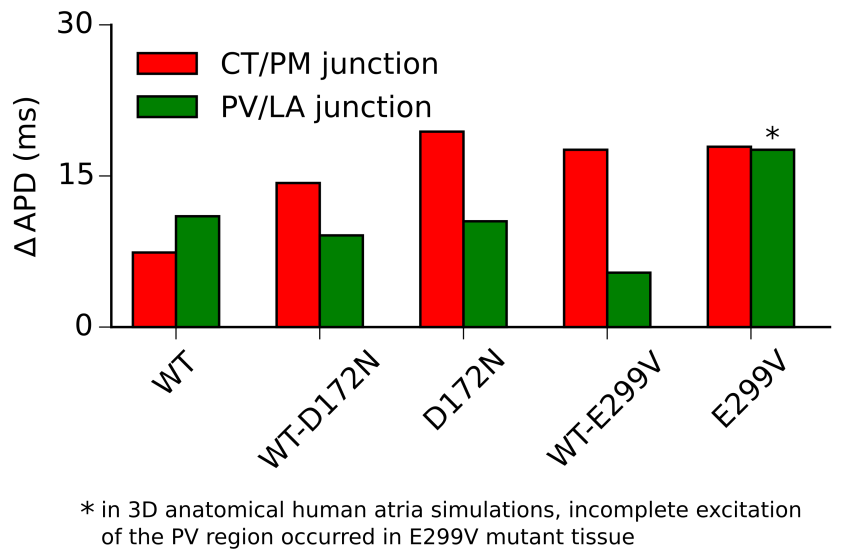


Fig S3. Differences in regional cell model tissue APD. ΔAPD at the CT/PM and PV/LA junctions as determined in 1D tissue models.
