# Supplementary material for: Atrial arrhythmogenicity of KCNJ2 mutations in short QT syndrome: Insights from virtual human atria
Source: PLoS Comput Biol. 2017 Jun 13;13(6):e1005593. doi: 10.1371/journal.pcbi.1005593 (PMC5487071; doi:10.1371/journal.pcbi.1005593)
Supplement: S4 Fig — Space-time plots of AP propagation in 1D models of the CT/PM junction used to compute temporal vulnerability to re-entry and corresponding vulnerability window (VW) widths. Three scenarios are shown which correspond to different S2 timings: bi-directional conduction block (A), uni-directional conduction (B), and bi-directional conduction (C). A summary of VW measurements in WT and SQT3 mutation conditions (D). (DOCX) [file pcbi.1005593.s005.docx]

**Fig S4**

**Atrial arrhythmogenicity of KCNJ2-linked short QT syndrome mutations: insights from virtual human atria**

Dominic G. Whittaker, Haibo Ni, Aziza El Harchi, Jules C. Hancox, Henggui Zhang


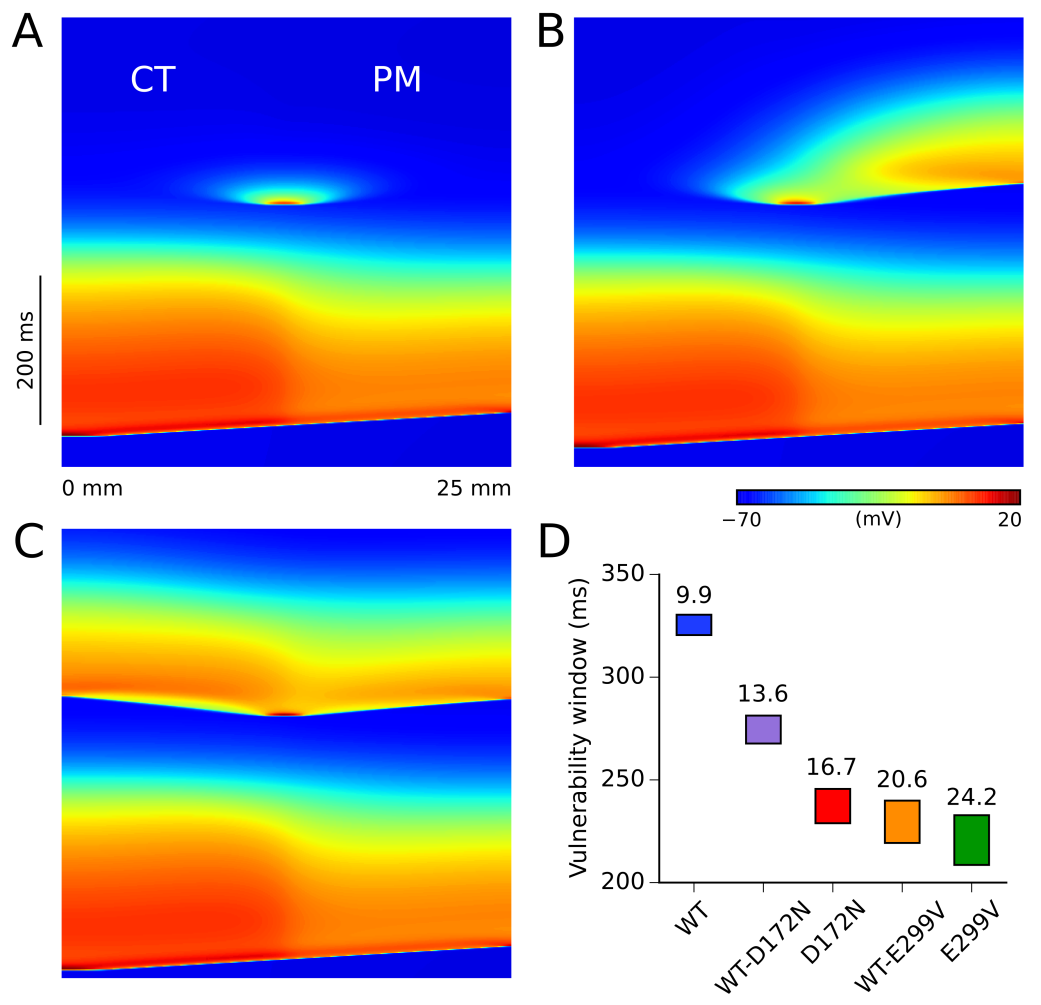


Fig S4. Vulnerability window to uni-directional conduction block. Space-time plots of AP propagation in 1D models of the CT/PM junction used to compute temporal vulnerability to re-entry and corresponding vulnerability window (VW) widths. Three scenarios are shown which correspond to different S2 timings: bi-directional conduction block (A), uni-directional conduction (B), and bi-directional conduction (C). A summary of VW measurements in WT and SQT3 mutation conditions (D).
