# Supplementary material for: Atrial arrhythmogenicity of KCNJ2 mutations in short QT syndrome: Insights from virtual human atria
Source: PLoS Comput Biol. 2017 Jun 13;13(6):e1005593. doi: 10.1371/journal.pcbi.1005593 (PMC5487071; doi:10.1371/journal.pcbi.1005593)
Supplement: S5 Fig — A summary of rotor trajectories in 2D re-entry simulations for different S2 timings after the effective refractory period (ERP). (DOCX) [file pcbi.1005593.s006.docx]

**Fig S5**

**Atrial arrhythmogenicity of KCNJ2-linked short QT syndrome mutations: insights from virtual human atria**

Dominic G. Whittaker, Haibo Ni, Aziza El Harchi, Jules C. Hancox, Henggui Zhang


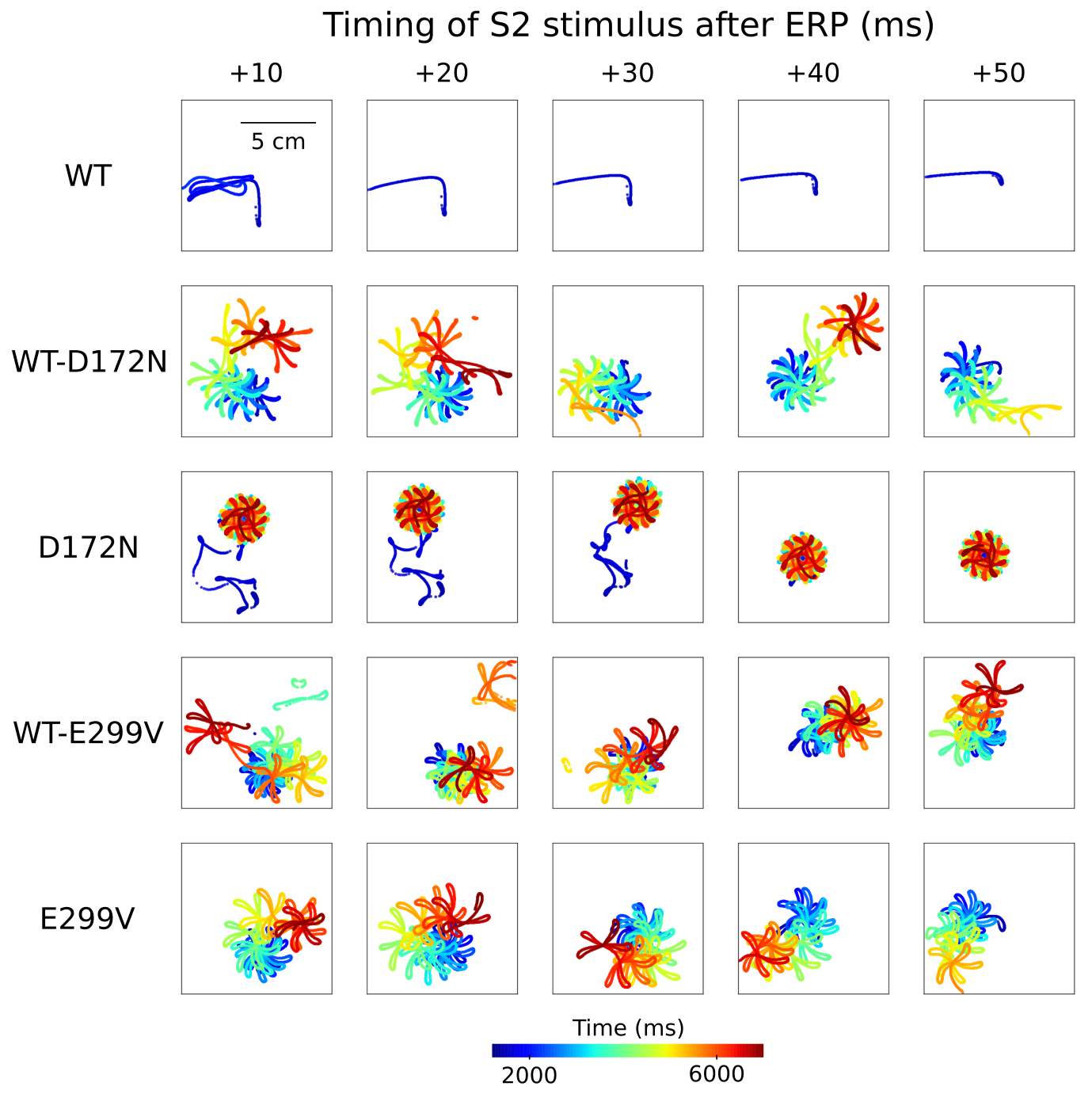


Fig S5. Re-entry simulations in idealised 2D sheet. A summary of rotor trajectories in 2D re-entry simulations for different S2 timings after the effective refractory period (ERP).
