# Supplementary material for: Atrial arrhythmogenicity of KCNJ2 mutations in short QT syndrome: Insights from virtual human atria
Source: PLoS Comput Biol. 2017 Jun 13;13(6):e1005593. doi: 10.1371/journal.pcbi.1005593 (PMC5487071; doi:10.1371/journal.pcbi.1005593)
Supplement: S6 Fig — (A) Bar charts showing the average lifespan of re-entrant excitations in 5 re-entry simulations corresponding to 5 different S2 timings; and (B) the average area of meander over time. (DOCX) [file pcbi.1005593.s007.docx]

**Fig S6**

**Atrial arrhythmogenicity of KCNJ2-linked short QT syndrome mutations: insights from virtual human atria**

Dominic G. Whittaker, Haibo Ni, Aziza El Harchi, Jules C. Hancox, Henggui Zhang


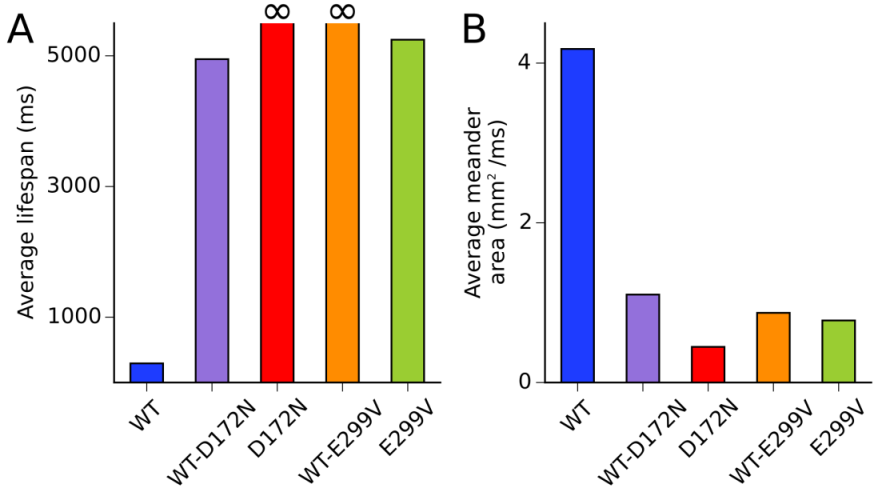


Fig S6. Spiral wave characteristics in 2D re-entry simulations. (A) Bar charts showing the average lifespan of re-entrant excitations in 5 re-entry simulations corresponding to 5 different S2 timings; and (B) the average area of meander over time.
