# Supplementary material for: Atrial arrhythmogenicity of KCNJ2 mutations in short QT syndrome: Insights from virtual human atria
Source: PLoS Comput Biol. 2017 Jun 13;13(6):e1005593. doi: 10.1371/journal.pcbi.1005593 (PMC5487071; doi:10.1371/journal.pcbi.1005593)
Supplement: S7 Fig — (A) WT ΔAPD is shown for reference. ΔAPD in WT-D172N (B) and WT-E299V (C) tissue is shown under the following conditions: (i) control, (ii) 50% IKr block, (iii) 50% IK1 block, (iv) 50% IK1+IKr block, (v) 50% IKur block, (vi) 50% IKr+IKur block, (vii) 100% increase in ICaL, and (viii) 250% increase in ICaL. The colour bar shows APD relative to the shortest APD measured in each condition, designated APD+. The scale of the colour bar is fixed at the value of ΔAPD in the WT condition (120 ms). Regions where APs failed to exceed −20 mV are shown in black. Single cell AP traces at 1 Hz pacing are shown in WT-D172N (Di, Dii) and WT-E299V (Diii, Div) conditions. (DOCX) [file pcbi.1005593.s008.docx]

**Fig S7**

**Atrial arrhythmogenicity of KCNJ2-linked short QT syndrome mutations: insights from virtual human atria**

Dominic G. Whittaker, Haibo Ni, Aziza El Harchi, Jules C. Hancox, Henggui Zhang


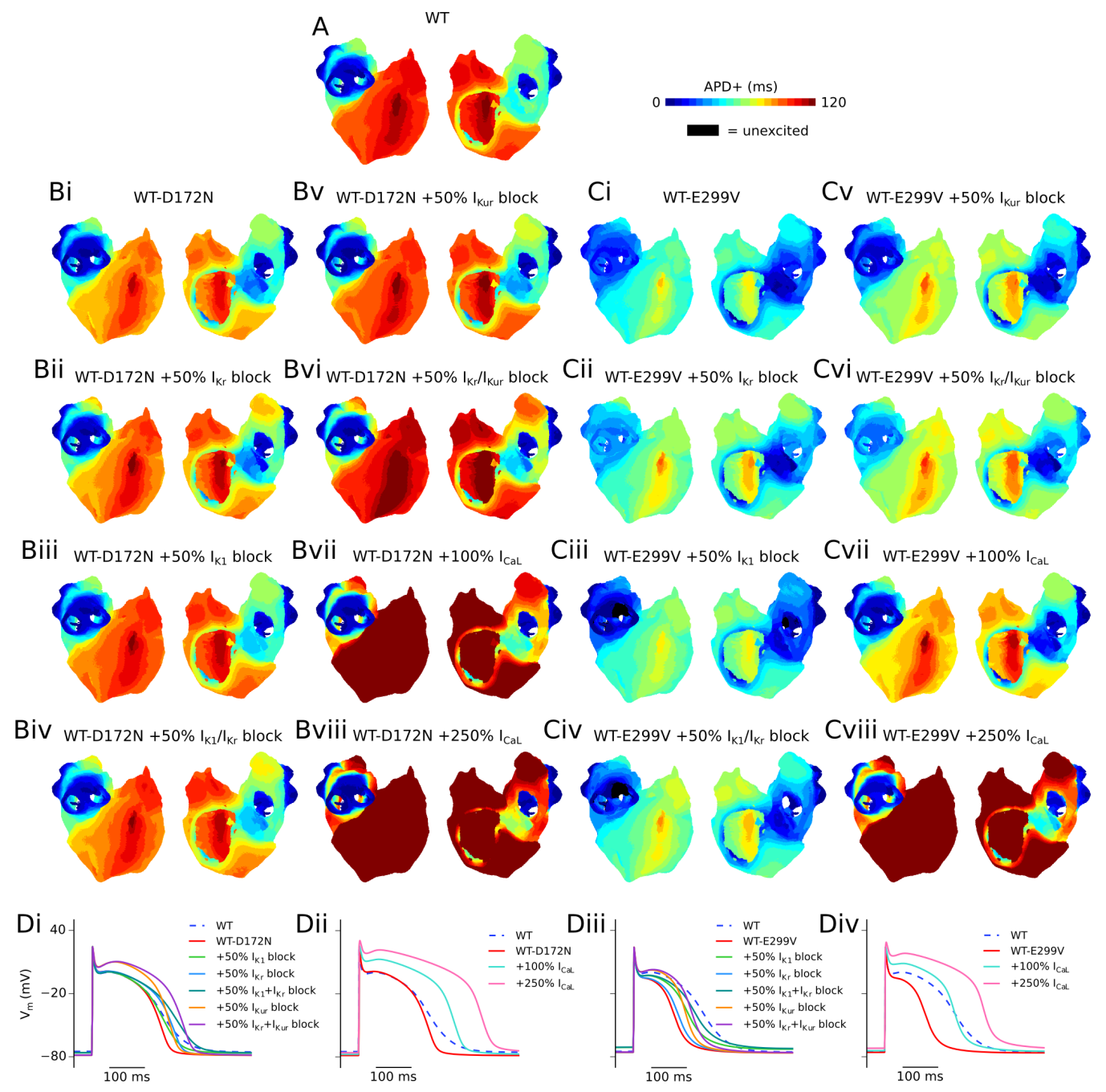


Fig S7. APD dispersion in 3D geometry under simulated pharmacological modulation conditions. (A) WT ΔAPD is shown for reference. ΔAPD in WT-D172N (B) and WT-E299V (C) tissue is shown under the following conditions: (i) control, (ii) 50% I_Kr_ block, (iii) 50% I_K1_ block, (iv) 50% I_K1_+I_Kr_ block, (v) 50% I_Kur_ block, (vi) 50% I_Kr_+I_Kur_ block, (vii) 100% increase in I_CaL_, and (viii) 250% increase in I_CaL_. The colour bar shows APD relative to the shortest APD measured in each condition, designated APD+. The scale of the colour bar is fixed at the value of ΔAPD in the WT condition (120 ms). Regions where APs failed to exceed −20 mV are shown in black. Single cell AP traces at 1 Hz pacing are shown in WT-D172N (Di, Dii) and WT-E299V (Diii, Div) conditions.
