# Supplementary material for: Atrial arrhythmogenicity of KCNJ2 mutations in short QT syndrome: Insights from virtual human atria
Source: PLoS Comput Biol. 2017 Jun 13;13(6):e1005593. doi: 10.1371/journal.pcbi.1005593 (PMC5487071; doi:10.1371/journal.pcbi.1005593)
Supplement: S8 Fig — Floating bar charts showing ΔAPD in WT-D172N (A) and WT-E299V (B) mutant tissue under various simulated drug effect conditions. The WT ΔAPD is shown in light blue for reference. (DOCX) [file pcbi.1005593.s009.docx]

**Fig S8**

**Atrial arrhythmogenicity of KCNJ2-linked short QT syndrome mutations: insights from virtual human atria**

Dominic G. Whittaker, Haibo Ni, Aziza El Harchi, Jules C. Hancox, Henggui Zhang


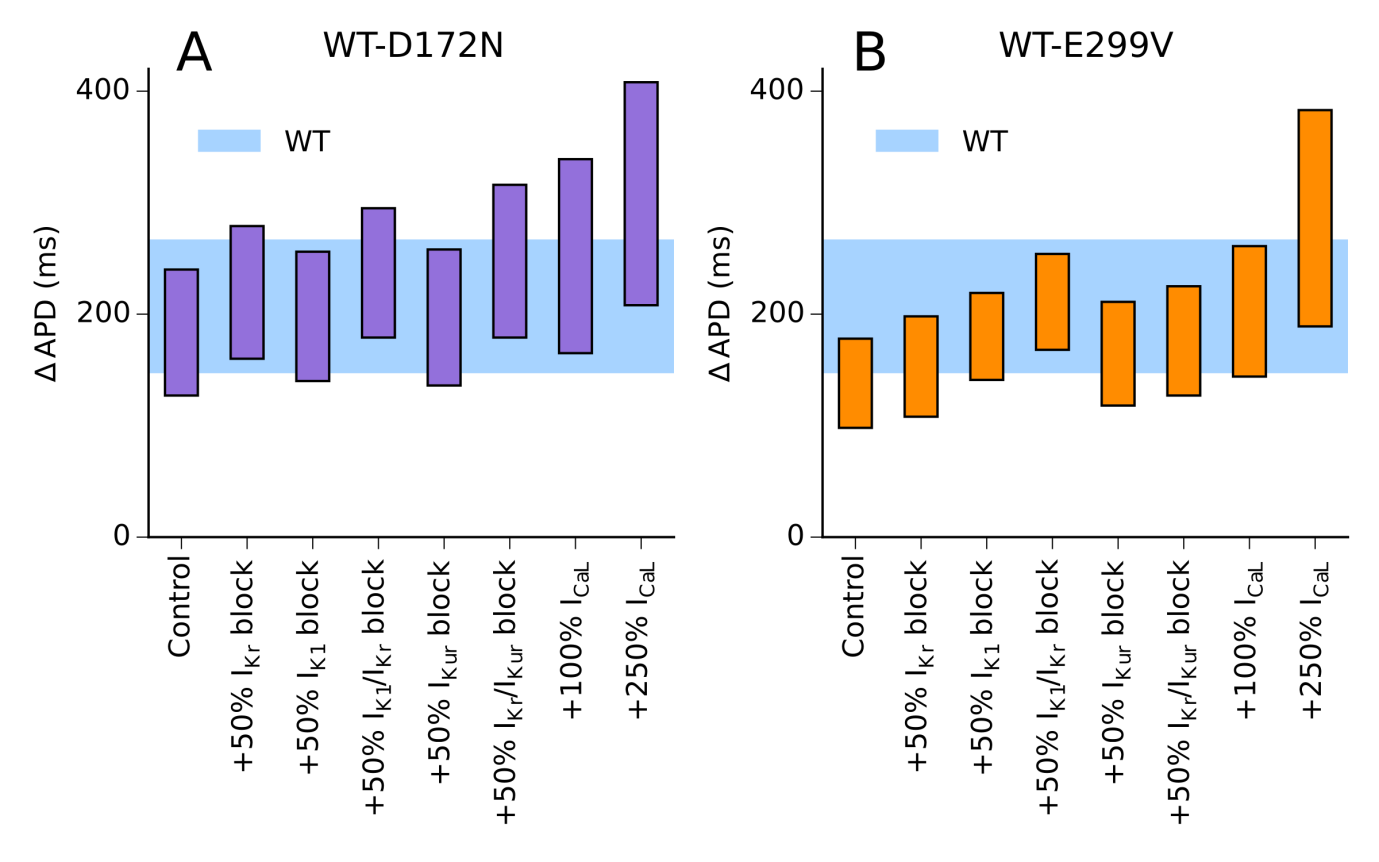


Fig S8. Summary of ΔAPD and relative APD prolongation in pharmacological simulation conditions. Floating bar charts showing ΔAPD in WT-D172N (A) and WT-E299V (B) mutant tissue under various simulated drug effect conditions. The WT ΔAPD is shown in light blue for reference.
