# Supplementary material for: Atrial arrhythmogenicity of KCNJ2 mutations in short QT syndrome: Insights from virtual human atria
Source: PLoS Comput Biol. 2017 Jun 13;13(6):e1005593. doi: 10.1371/journal.pcbi.1005593 (PMC5487071; doi:10.1371/journal.pcbi.1005593)
Supplement: S9 Fig — (A) Mapping of the membrane potential from an action potential onto the realistic 3D human atria geometry. (B) Evolution of scroll waves in the RA at different time points using phase distribution method initial conditions. (DOCX) [file pcbi.1005593.s010.docx]

**Fig S9**

**Atrial arrhythmogenicity of KCNJ2-linked short QT syndrome mutations: insights from virtual human atria**

Dominic G. Whittaker, Haibo Ni, Aziza El Harchi, Jules C. Hancox, Henggui Zhang


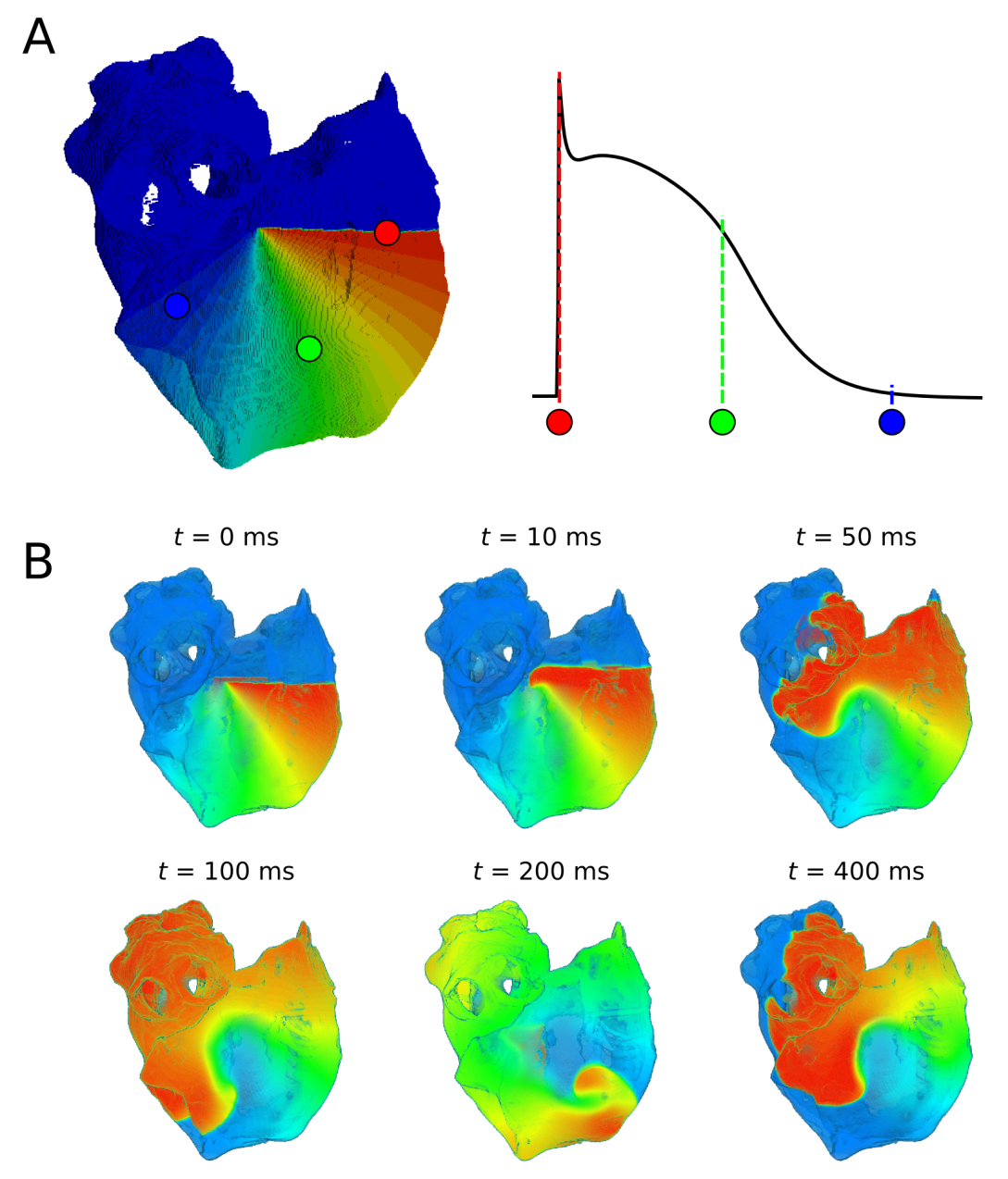


Fig S9. Illustration of the phase distribution method and evolution of scroll waves. (A) Mapping of the membrane potential from an action potential onto the realistic 3D human atria geometry. (B) Evolution of scroll waves in the RA at different time points using phase distribution method initial conditions.
