# Supplementary material for: Atrial arrhythmogenicity of KCNJ2 mutations in short QT syndrome: Insights from virtual human atria
Source: PLoS Comput Biol. 2017 Jun 13;13(6):e1005593. doi: 10.1371/journal.pcbi.1005593 (PMC5487071; doi:10.1371/journal.pcbi.1005593)
Supplement: S10 Fig — Action potential waveforms in WT, WT-D172N, D172N, WT-E299V, and E299V conditions at a pacing frequency of 1 Hz (Ai), with corresponding current trace for IK1 (Aii). Restitution of the APD90 (Bi), and maximal slope of restitution (Bii). (DOCX) [file pcbi.1005593.s011.docx]

**Fig S10**

**Atrial arrhythmogenicity of KCNJ2-linked short QT syndrome mutations: insights from virtual human atria**

Dominic G. Whittaker, Haibo Ni, Aziza El Harchi, Jules C. Hancox, Henggui Zhang


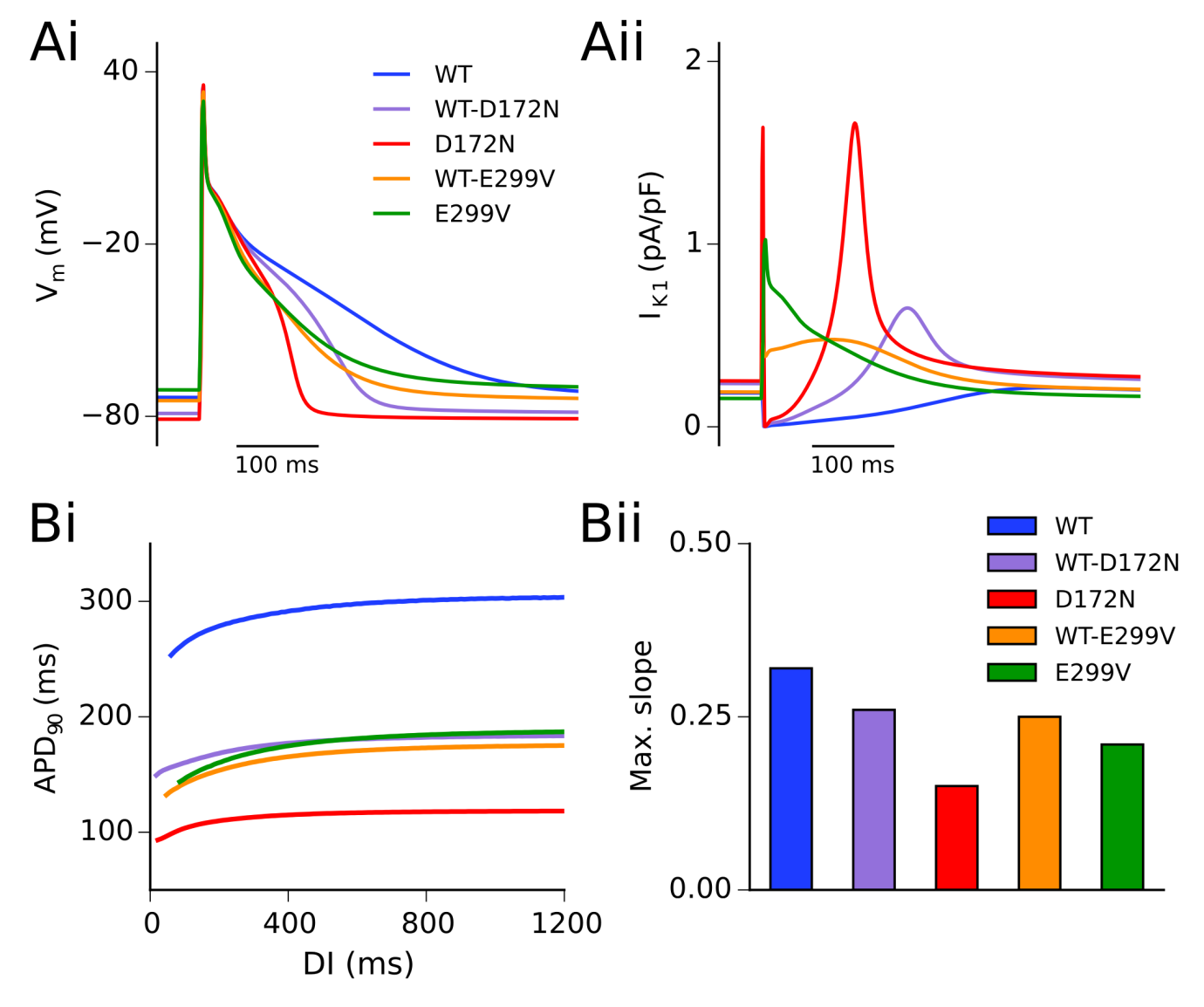


**Fig S10. AP and I_K1_ profile in WT and mutation conditions using the GB model.** Action potential waveforms in WT, WT-D172N, D172N, WT-E299V, and E299V conditions at a pacing frequency of 1 Hz (Ai), with corresponding current trace for I_K1_ (Aii). Restitution of the APD_90_ (Bi), and maximal slope of restitution (Bii).
