# Supplementary material for: Atrial arrhythmogenicity of KCNJ2 mutations in short QT syndrome: Insights from virtual human atria
Source: PLoS Comput Biol. 2017 Jun 13;13(6):e1005593. doi: 10.1371/journal.pcbi.1005593 (PMC5487071; doi:10.1371/journal.pcbi.1005593)
Supplement: S11 Fig — Regional cell models in the GB model (A), including PM, RAA, CT, BB, AS, LA, LAA, AVR, PV. APD distribution maps in WT (Bi), WT-D172N (Bii), D172N (Biii), WT-E299V (Biv), and E299V (Bv) mutation conditions, with corresponding ΔAPD (C). The colour bar shows APD relative to the shortest APD measured in each condition, designated APD+. The scale of the colour bar is fixed at the value of ΔAPD in the WT condition (72 ms). The colour black shows regions where membrane potentials failed to exceed a threshold value (−20 mV). (DOCX) [file pcbi.1005593.s012.docx]

**Fig S11**

**Atrial arrhythmogenicity of KCNJ2-linked short QT syndrome mutations: insights from virtual human atria**

Dominic G. Whittaker, Haibo Ni, Aziza El Harchi, Jules C. Hancox, Henggui Zhang


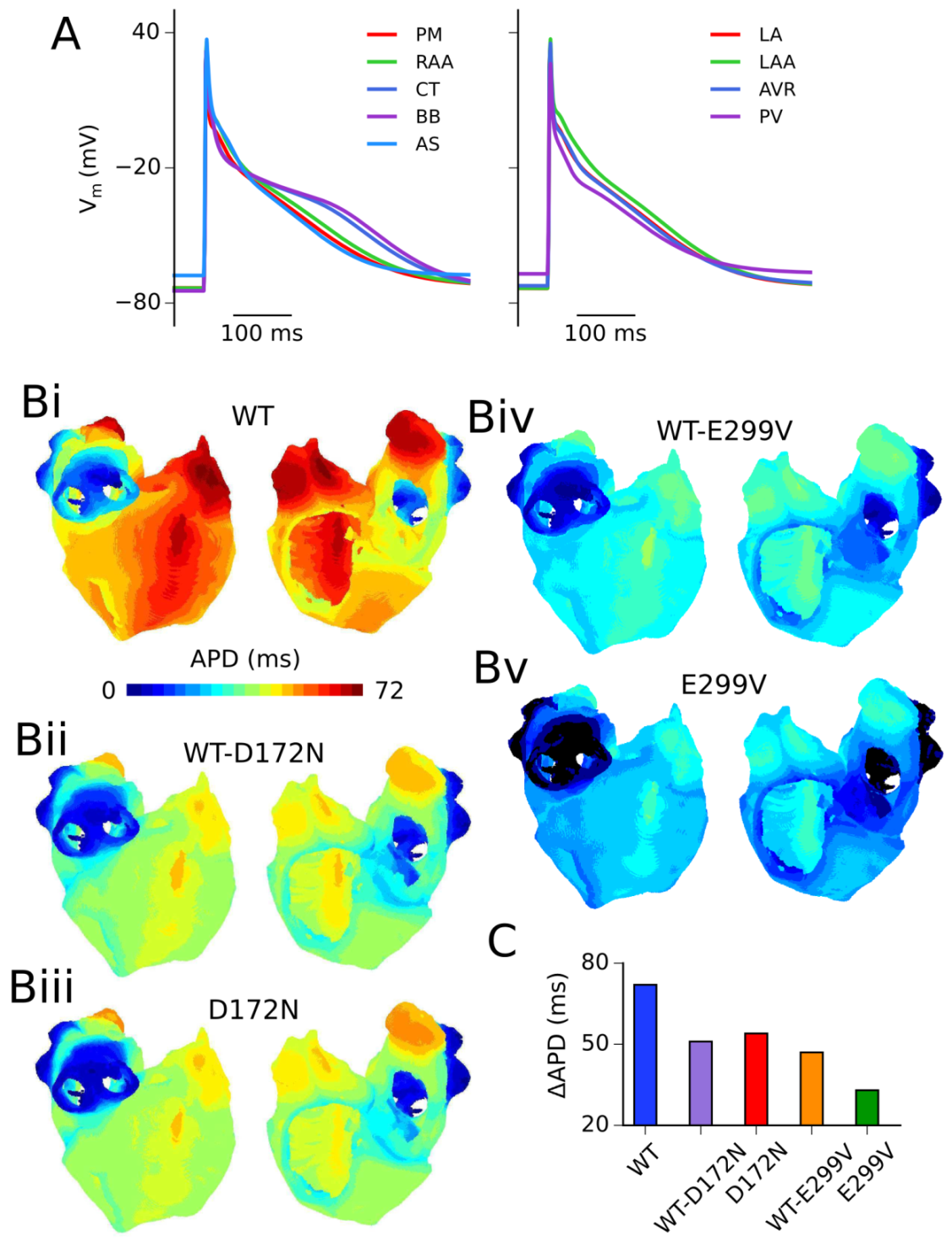


**Fig S11. Regional cell models and spatial dispersion of APD in GB model.** Regional cell models in the GB model (A), including PM, RAA, CT, BB, AS, LA, LAA, AVR, PV. APD distribution maps in WT (Bi), WT-D172N (Bii), D172N (Biii), WT-E299V (Biv), and E299V (Bv) mutation conditions, with corresponding ΔAPD (C). The colour bar shows APD relative to the shortest APD measured in each condition, designated APD+. The scale of the colour bar is fixed at the value of ΔAPD in the WT condition (72 ms). The colour black shows regions where membrane potentials failed to exceed a threshold value (−20 mV).
