# Supplementary material for: Atrial arrhythmogenicity of KCNJ2 mutations in short QT syndrome: Insights from virtual human atria
Source: PLoS Comput Biol. 2017 Jun 13;13(6):e1005593. doi: 10.1371/journal.pcbi.1005593 (PMC5487071; doi:10.1371/journal.pcbi.1005593)
Supplement: S12 Fig — (A) Simulated I-V relationships for the WT IK1 formulation used in the human atrial cell model in the present study compared with the WT IK1 formulation used in our previous study in human ventricular cells (Adeniran et al., 2012)–the inset shows native IK1 recordings in human atrial and ventricular myocytes taken from Wang et al., 1998. (B) Simulated I-V relationships for IK1 used in this study compared with IK1 from the GPB model (Grandi et al., 2011), CRN model (Courtemanche et al., 1998), and the NFG model (Nygren et al., 1998). (DOCX) [file pcbi.1005593.s013.docx]

**Fig S12**

**Atrial arrhythmogenicity of KCNJ2-linked short QT syndrome mutations: insights from virtual human atria**

Dominic G. Whittaker, Haibo Ni, Aziza El Harchi, Jules C. Hancox, Henggui Zhang


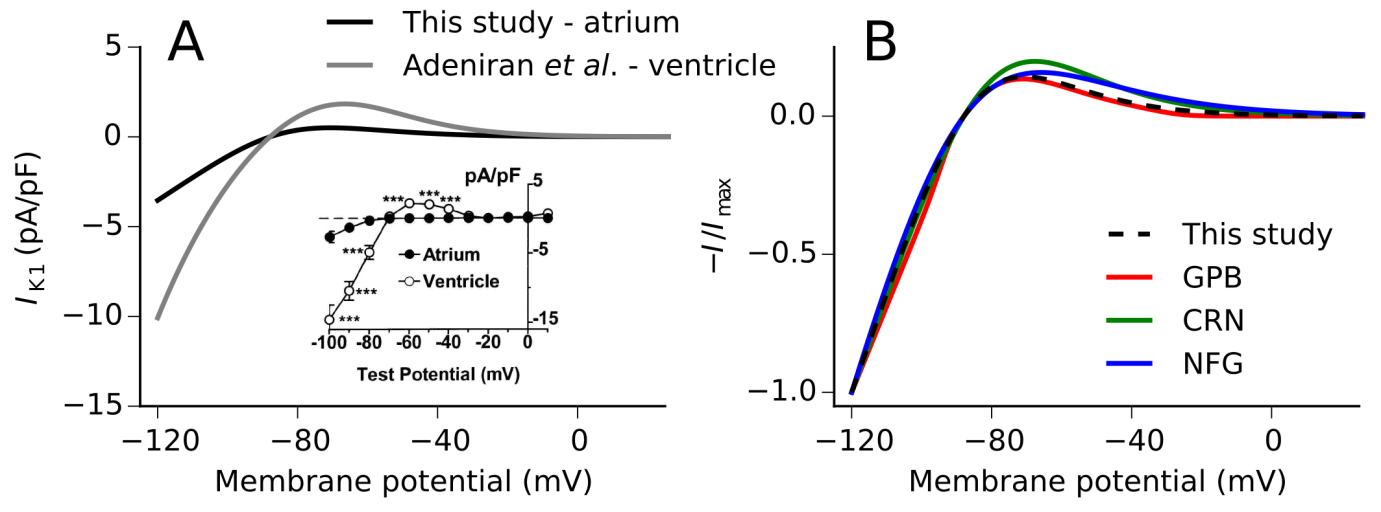


Fig S12. Comparison of I_K1_ kinetics with previous studies. (A) Simulated I-V relationships for the WT I_K1_ formulation used in the human atrial cell model in the present study compared with the WT I_K1_ formulation used in our previous study in human ventricular cells [1] – the inset shows native I_K1_ recordings in human atrial and ventricular myocytes taken from [2]. (B) Simulated I-V relationships for I_K1_ used in this study compared with I_K1_ from the Grandi *et al*. (GPB) model [3], Courtemanche *et al*. (CRN) model [4], and the Nygren *et al*. (NFG) model [5].

1. Adeniran I, Harchi AE, Hancox JC, Zhang H. Proarrhythmia in KCNJ2-linked short QT syndrome: insights from modelling. Cardiovasc Res. 2012;94: 66–76. doi:10.1093/cvr/cvs082

2. Wang Z, Yue L, White M, Pelletier G, Nattel S. Differential Distribution of Inward Rectifier Potassium Channel Transcripts in Human Atrium Versus Ventricle. Circulation. 1998;98: 2422–2428. doi:10.1161/01.CIR.98.22.2422

3. Grandi E, Pandit SV, Voigt N, Workman AJ, Dobrev D, Jalife J, et al. Human Atrial Action Potential and Ca2+ Model: Sinus Rhythm and Chronic Atrial Fibrillation. Circ Res. 2011;109: 1055–1066. doi:10.1161/CIRCRESAHA.111.253955

4. Courtemanche M, Ramirez RJ, Nattel S. Ionic mechanisms underlying human atrial action potential properties: insights from a mathematical model. Am J Physiol - Heart Circ Physiol. 1998;275: H301–H321.

5. Nygren A, Fiset C, Firek L, Clark JW, Lindblad DS, Clark RB, et al. Mathematical Model of an Adult Human Atrial Cell The Role of K+ Currents in Repolarization. Circ Res. 1998;82: 63–81. doi:10.1161/01.RES.82.1.63
