# Supplementary material for: Atrial arrhythmogenicity of KCNJ2 mutations in short QT syndrome: Insights from virtual human atria
Source: PLoS Comput Biol. 2017 Jun 13;13(6):e1005593. doi: 10.1371/journal.pcbi.1005593 (PMC5487071; doi:10.1371/journal.pcbi.1005593)
Supplement: S1 Table — Parameters of IK1 for WT, WT-D172N, D172N, WT-E299V, and E299V mutation conditions, obtained by fitting Equation S1 to experimental data (El Harchi et al., 2009; Deo et al., 2013). For comparison, formulations of the WT model used in Kharche et al., 2008; and the CRN model IK1 (Courtemanche et al., 1998) are shown. (DOCX) [file pcbi.1005593.s014.docx]

**Table S1**

**Atrial arrhythmogenicity of KCNJ2-linked short QT syndrome mutations: insights from virtual human atria**

Dominic G. Whittaker, Haibo Ni, Aziza El Harchi, Jules C. Hancox, Henggui Zhang

Table S1. I_K1_ formulation parameters.

|  | ***g*_K1,max_ (nS/pF)** | ***a*** | ***b* (mV^-1^)** | ***c* (mV)** |
| --- | --- | --- | --- | --- |
| **WT** | 0.118 | 0 | 0.077 | -85.00 |
| **WT-D172N** | 0.154 | 0 | 0.0847 | -69.26 |
| **D172N** | 0.277 | 0 | 0.09317 | -62.00 |
| **WT-E299V** | 0.107 | 0.0385 | 0.0323 | -94.27 |
| **E299V** | 0.107 | 0.1456 | 0.0369 | -139.54 |
| **Kharche *et al*. WT** [1] | 3.650 | 0.0482 | 0.086 | -77.80 |
| **CRN model** [2] | 0.090 | 0 | 0.070 | -80.00 |

Parameters of I_K1_ for WT, WT-D172N, D172N, WT-E299V, and E299V mutation conditions obtained by fitting Equation S1 to experimental data [3,4]. For comparison, formulations of the WT model used in [1], and the CRN model I_K1_ are shown [2].

1. Kharche S, Garratt CJ, Boyett MR, Inada S, Holden AV, Hancox JC, et al. Atrial proarrhythmia due to increased inward rectifier current (IK1) arising from KCNJ2 mutation – A simulation study. Prog Biophys Mol Biol. 2008;98: 186–197. doi:10.1016/j.pbiomolbio.2008.10.010

2. Courtemanche M, Ramirez RJ, Nattel S. Ionic mechanisms underlying human atrial action potential properties: insights from a mathematical model. Am J Physiol - Heart Circ Physiol. 1998;275: H301–H321.

3. El Harchi A, McPate MJ, Zhang Y hong, Zhang H, Hancox JC. Action potential clamp and chloroquine sensitivity of mutant Kir2.1 channels responsible for variant 3 short QT syndrome. J Mol Cell Cardiol. 2009;47: 743–747. doi:10.1016/j.yjmcc.2009.02.027

4. Deo M, Ruan Y, Pandit SV, Shah K, Berenfeld O, Blaufox A, et al. KCNJ2 mutation in short QT syndrome 3 results in atrial fibrillation and ventricular proarrhythmia. Proc Natl Acad Sci. 2013;110: 4291–4296. doi:10.1073/pnas.1218154110
