# Supplementary material for: Atrial arrhythmogenicity of KCNJ2 mutations in short QT syndrome: Insights from virtual human atria
Source: PLoS Comput Biol. 2017 Jun 13;13(6):e1005593. doi: 10.1371/journal.pcbi.1005593 (PMC5487071; doi:10.1371/journal.pcbi.1005593)
Supplement: S2 Table — A summary of conductance scaling factors, GX, for maximal conductance of ionic current IX relative to the baseline (RA) cell model and corresponding experimental data sources. Abbreviations are as follows: CT = crista terminalis, BB = Bachmann’s bundle, PM = pectinate muscles, AVR = atrio-ventricular ring, RAA = right atrial appendage, AS = atrial septum, LA = left atrium, LAA = left atrial appendage, PV = pulmonary veins. (DOCX) [file pcbi.1005593.s015.docx]

**Table S2**

**Atrial arrhythmogenicity of KCNJ2-linked short QT syndrome mutations: insights from virtual human atria**

Dominic G. Whittaker, Haibo Ni, Aziza El Harchi, Jules C. Hancox, Henggui Zhang

Table S2. Ionic differences in regional cell models.

|  | **G_CaL_** | **G_to_** | **G_Kur_** | **G_Na_** | **G_Kr_** | **G_Ks_** | **G_K1_** | **Source** |
| --- | --- | --- | --- | --- | --- | --- | --- | --- |
| **CT** | 1.68 | 1.0 | 1.0 | 1.0 | 1.0 | 1.0 | 1.0 | [1] |
| **BB** | 1.72 | 1.0 | 1.0 | 1.0 | 1.0 | 1.0 | 1.0 | [1,2] |
| **PM** | 0.94 | 1.0 | 1.0 | 1.0 | 1.0 | 1.0 | 1.0 | [1] |
| **AVR** | 0.67 | 0.6 | 1.0 | 1.0 | 1.63 | 1.0 | 1.0 | [1] |
| **RAA** | 1.0 | 0.68 | 1.0 | 1.0 | 1.0 | 1.0 | 1.0 | [1,3] |
| **AS** | 0.4 | 0.212 | 0.667 | 1.3 | 1.0 | 1.0 | V_1/2_ −6 | [3] |
| **LA** | 1.0 | 1.0 | 1.0 | 1.0 | 1.6 | 1.0 | 1.0 | [1,4,5] |
| **LAA** | 1.0 | 0.68 | 0.8 | 1.0 | 1.6 | 1.0 | 1.0 | [1,4,6] |
| **PV** | 0.7 | 0.75 | 1.0 | 1.0 | 2.4 | 1.5 | 0.62  V_1/2_ −7 | [5,7,8] |

A summary of conductance scaling factors, G_X_, for maximal conductance of ionic current I_X_ relative to the baseline (RA) cell model and corresponding experimental data sources. Abbreviations are as follows: CT = crista terminalis, BB = Bachmann’s bundle, PM = pectinate muscles, AVR = atrio-ventricular ring, RAA = right atrial appendage, AS = atrial septum, LA = left atrium, LAA = left atrial appendage, PV = pulmonary veins.

1. Feng J, Yue L, Wang Z, Nattel S. Ionic Mechanisms of Regional Action Potential Heterogeneity in the Canine Right Atrium. Circ Res. 1998;83: 541–551. doi:10.1161/01.RES.83.5.541

2. Burashnikov A, Mannava S, Antzelevitch C. Transmembrane action potential heterogeneity in the canine isolated arterially perfused right atrium: effect of IKr and IKur/Ito block. Am J Physiol - Heart Circ Physiol. 2004;286: H2393–H2400. doi:10.1152/ajpheart.01242.2003

3. Gong D, Zhang Y, Cai B, Meng Q, Jiang S, Li X, et al. Characterization and comparison of Na+, K+ and Ca2+ currents between myocytes from human atrial right appendage and atrial septum. Cell Physiol Biochem Int J Exp Cell Physiol Biochem Pharmacol. 2008;21: 385–394. doi:10.1159/000129631

4. Li D, Zhang L, Kneller J, Nattel S. Potential Ionic Mechanism for Repolarization Differences Between Canine Right and Left Atrium. Circ Res. 2001;88: 1168–1175. doi:10.1161/hh1101.091266

5. Ehrlich JR, Cha T-J, Zhang L, Chartier D, Melnyk P, Hohnloser SH, et al. Cellular electrophysiology of canine pulmonary vein cardiomyocytes: action potential and ionic current properties. J Physiol. 2003;551: 801–813. doi:10.1113/jphysiol.2003.046417

6. Caballero R, de la Fuente MG, Gómez R, Barana A, Amorós I, Dolz-Gaitón P, et al. In Humans, Chronic Atrial Fibrillation Decreases the Transient Outward Current and Ultrarapid Component of the Delayed Rectifier Current Differentially on Each Atria and Increases the Slow Component of the Delayed Rectifier Current in Both. J Am Coll Cardiol. 2010;55: 2346–2354. doi:10.1016/j.jacc.2010.02.028

7. Cha T-J, Ehrlich JR, Zhang L, Chartier D, Leung TK, Nattel S. Atrial Tachycardia Remodeling of Pulmonary Vein Cardiomyocytes. Circulation. 2005;111: 728–735. doi:10.1161/01.CIR.0000155240.05251.D0

8. Datino T, Macle L, Qi X-Y, Maguy A, Comtois P, Chartier D, et al. Mechanisms by Which Adenosine Restores Conduction in Dormant Canine Pulmonary Veins. Circulation. 2010;121: 963–972. doi:10.1161/CIRCULATIONAHA.109.893107
