# Supplementary material for: Atrial arrhythmogenicity of KCNJ2 mutations in short QT syndrome: Insights from virtual human atria
Source: PLoS Comput Biol. 2017 Jun 13;13(6):e1005593. doi: 10.1371/journal.pcbi.1005593 (PMC5487071; doi:10.1371/journal.pcbi.1005593)
Supplement: S3 Table — A summary of dominant frequencies (DF) in SQT3 mutation conditions in a representative 2D spiral wave re-entry simulation. (DOCX) [file pcbi.1005593.s016.docx]

**Table S3**

**Atrial arrhythmogenicity of KCNJ2-linked short QT syndrome mutations: insights from virtual human atria**

Dominic G. Whittaker, Haibo Ni, Aziza El Harchi, Jules C. Hancox, Henggui Zhang

Table S3. Dominant frequency in 2D re-entry simulations.

|  | **WT** | **WT-D172N** | **D172N** | **WT-E299V** | **E299V** |
| --- | --- | --- | --- | --- | --- |
| **DF (Hz)** | N/A | 5.69 | 7.20 | 5.60 | 5.20 |

A summary of dominant frequencies (DF) in SQT3 mutation conditions in a representative 2D spiral wave re-entry simulation.
