# Supplementary material for: Atrial arrhythmogenicity of KCNJ2 mutations in short QT syndrome: Insights from virtual human atria
Source: PLoS Comput Biol. 2017 Jun 13;13(6):e1005593. doi: 10.1371/journal.pcbi.1005593 (PMC5487071; doi:10.1371/journal.pcbi.1005593)
Supplement: S4 Table — A summary of AP properties such as action potential amplitude (APA), resting membrane potential (RMP), action potential duration at 50% and 90% repolarisation (APD50 and APD90, respectively), and maximum upstroke velocity (MUV) in the GB model in WT and SQT3 mutation conditions at a pacing frequency of 1 Hz. (DOCX) [file pcbi.1005593.s017.docx]

**Table S4**

**Atrial arrhythmogenicity of KCNJ2-linked short QT syndrome mutations: insights from virtual human atria**

Dominic G. Whittaker, Haibo Ni, Aziza El Harchi, Jules C. Hancox, Henggui Zhang

Table S4. AP properties in WT and SQT3 mutant conditions in the GB model.

|  | **APA (mV)** | **RMP (mV)** | **APD_50_ (ms)** | **APD_90_ (ms)** | **MUV (V/s)** |
| --- | --- | --- | --- | --- | --- |
| **WT** | 106.6 | -73.4 | 59.5 | 299.6 | 168.0 |
| **WT-D172N** | 114.3 | -79.0 | 59.8 | 182.4 | 197.9 |
| **D172N** | 116.5 | -81.0 | 57.3 | 117.9 | 206.3 |
| **WT-E299V** | 107.6 | -74.5 | 46.5 | 173.3 | 174.4 |
| **E299V** | 100.5 | -70.8 | 42.6 | 184.6 | 134.9 |

A summary of AP properties such as action potential amplitude (APA), resting membrane potential (RMP), action potential duration at 50% and 90% repolarisation (APD_50_ and APD_90_, respectively), and maximum upstroke velocity (MUV) in the GB model in WT and SQT3 mutation conditions at a pacing frequency of 1 Hz.
