# Supplementary material for: Correction: Atrial arrhythmogenicity of KCNJ2 mutations in short QT syndrome: Insights from virtual human atria
Source: PLoS Comput Biol. 2019 Jun 13;15(6):e1007145. doi: 10.1371/journal.pcbi.1007145 (PMC6563946; doi:10.1371/journal.pcbi.1007145)
Supplement: S1 File — (PDF) [file pcbi.1007145.s001.pdf]

PLOS COMPUTATIONAL BIOLOGY

RESEARCH ARTICLE

Atrial arrhythmogenicity of *KCNJ2* mutations in short QT syndrome: Insights from virtual human atria

Dominic G. Whittaker[1], Haibo Ni[1], Aziza El Harchi[2], Jules C. Hancox[1,2]*, Henggui Zhang[1,3,4]*

1 Biological Physics Group, School of Physics & Astronomy, The University of Manchester, Manchester, United Kingdom, **2** Department of Physiology, Pharmacology and Neuroscience, and Cardiovascular Research Laboratories, School of Medical Sciences, University of Bristol, Bristol, United Kingdom, **3** School of Computer Science and Technology, Harbin Institute of Technology, Harbin, China, **4** Space Institute of Southern China, Shenzhen, China

* henggui.zhang@manchester.ac.uk (HZ); jules.hancox@bristol.ac.uk (JCH)

Check for updates

OPEN ACCESS

Citation: Whittaker DG, Ni H, El Harchi A, Hancox JC, Zhang H (2017) Atrial arrhythmogenicity of *KCNJ2* mutations in short QT syndrome: Insights from virtual human atria. PLoS Comput Biol 13(6): e1005593. https://doi.org/10.1371/journal.pcbi.1005593

Editor: Alexander V Panfilov, Universiteit Gent, BELGIUM

Received: March 21, 2017

Accepted: May 25, 2017

Published: June 13, 2017

Copyright: © 2017 Whittaker et al. This is an open access article distributed under the terms of the Creative Commons Attribution License, which permits unrestricted use, distribution, and reproduction in any medium, provided the original author and source are credited.

Data Availability Statement: All relevant data are within the paper and its Supporting Information files.

Funding: The work is was supported by the British Heart Foundation (FS/14/5/30533—HZ; PG/06/147—JCH), Heart Research UK (RG2640—JCH), Engineering and Physical Science Research Council (EP/J00958X/1; EP/I029826/1—HZ), Marie Curie – IRSES CORDIS3D (317766—HZ), Natural Science Foundation of China (61179009—HZ), Shenzhen Science and Technology Innovation

Abstract

Gain-of-function mutations in *KCNJ2*-encoded Kir2.1 channels underlie variant 3 (SQT3) of the short QT syndrome, which is associated with atrial fibrillation (AF). Using biophysically-detailed human atria computer models, this study investigated the mechanistic link between SQT3 mutations and atrial arrhythmogenesis, and potential ion channel targets for treatment of SQT3. A contemporary model of the human atrial action potential (AP) was modified to recapitulate functional changes in I_{K1} due to heterozygous and homozygous forms of the D172N and E299V Kir2.1 mutations. Wild-type (WT) and mutant formulations were incorporated into multi-scale homogeneous and heterogeneous tissue models. Effects of mutations on AP duration (APD), conduction velocity (CV), effective refractory period (ERP), tissue excitation threshold and their rate-dependence, as well as the wavelength of re-entry (WL) were quantified. The D172N and E299V Kir2.1 mutations produced distinct effects on I_{K1} and APD shortening. Both mutations decreased WL for re-entry through a reduction in ERP and CV. Stability of re-entrant excitation waves in 2D and 3D tissue models was mediated by changes to tissue excitability and dispersion of APD in mutation conditions. Combined block of I_{K1} and I_{Kr} was effective in terminating re-entry associated with heterozygous D172N conditions, whereas I_{Kr} block alone may be a safer alternative for the E299V mutation. Combined inhibition of I_{Kr} and I_{Kur} produced a synergistic anti-arrhythmic effect in both forms of SQT3. In conclusion, this study provides mechanistic insights into atrial proarrhythmia with SQT3 Kir2.1 mutations and highlights possible pharmacological strategies for management of SQT3-linked AF.

Author summary

Atrial fibrillation (AF) is the most common cardiac arrhythmia, and is characterised by complex and irregular electrical activation of the upper chambers of the heart. One rare,

Committee (JCYJ20151029173639477; JSGG20160229125049615—HZ). The funders had no role in study design, data collection and analysis, decision to publish, or preparation of the manuscript.

Competing interests: The authors have declared that no competing interests exist.

genetic condition associated with increased risk of AF is the short QT syndrome (SQTS), which is caused by mutations in genes involved in normal electrical function of the heart. Underlying mechanisms by which SQTS-related gene mutations facilitate development of arrhythmias in the human atria are not well understood. In this study, sophisticated computer models representing 'virtual' human atria, incorporating detailed electrophysiological data at the 'ion channel' protein level into both idealised and realistic multi-scale tissue geometries, were used to dissect mechanisms by which two mutations in the *KCNJ2* gene responsible for SQTS variant 3 (SQT3) promote initiation and sustenance of arrhythmias. It was found that the D172N and E299V mutations to *KCNJ2* accelerated the repolarisation process at the cellular level through distinct mechanisms. This, along with the way the mutations affected heterogeneity in electrical behaviour at the organ level, mediated stability of arrhythmias and response to simulated ion channel block. This study improves understanding of mechanisms underlying increased AF risk associated with D172N and E299V *KCNJ2* mutations, and outlines potential therapeutic strategies.

Introduction

The cardiac inward rectifier potassium current (I_{K1}) is responsible for stabilising the resting membrane potential (RMP) and contributes to terminal repolarisation of both atrial and ventricular action potentials (APs) [1]. The K^+ channels that mediate I_{K1} are comprised of Kir2.x family of subunits, of which the *KCNJ2*-encoded Kir2.1 is strongly expressed in both the atria and ventricles [2]. Loss-of-function of Kir2.1 channels has been implicated in the Andersen-Tawil syndrome (long QT syndrome type 7) and causes ventricular arrhythmias and a range of extracardiac abnormalities [3]. Gain-of-function *KCNJ2* mutations are also potentially life-threatening, underlying variant 3 of the short QT syndrome (SQT3) [4–6], as well as familial atrial fibrillation (AF) [7].

The short QT syndrome (SQTS) is a genetic disorder that has been associated with increased risk of ventricular *and* atrial arrhythmias, and of sudden cardiac death [8,9]. The SQTS phenotype is characterised by an abbreviated QT interval, tall and peaked T waves, and loss of rate-adaptation of the QT interval [9]. To date, four missense mutations have been identified in *KCNJ2*-linked short QT syndrome (SQT3): D172N [4], M301K [5], E299V [6], and K346T [10], with reports of AF in some patients [5,6]. The first reported SQT3 mutation, D172N Kir2.1 [4], was shown to increase significantly outward I_{K1} at potentials between −75 mV and −45 mV. The proband and her father exhibited significantly shortened QT_c intervals (315 ms and 320 ms, respectively) and presented with a history of presyncopal events and palpitations [4]. The subsequently discovered E299V Kir2.1 mutation was shown to differ from D172N in that it strongly affects inward rectification, resulting in large outward I_{K1} currents at potentials above −55mV [6]. The proband presented with an extremely short QT interval (200 ms) which showed no rate-adaptation, and recurring episodes of paroxysmal AF [6].

A link between increased I_{K1} and *acquired* AF is well-established, with upregulation of I_{K1} consistently observed in chronic AF-induced electrical remodelling of the human atrial action potential (AP) [11–14]. Previously it has been shown that increased I_{K1} shortened the action potential duration (APD) and stabilised rotors in computational models of human atrial electrophysiology [15–18]. Increased I_{K1} arising from the E299V Kir2.1 mutation has also been shown to be pro-arrhythmic at cellular and 2D tissue levels [6]. However, the atria are characterised by marked electrical heterogeneity [19–22], which has been shown previously to generate an arrhythmia substrate *in silico* [23,24]. Mechanisms underlying initiation and

maintenance of atrial arrhythmias at cellular and organ levels arising from interactions between genetic mutations and intrinsic electrical heterogeneities in human atria have yet to be investigated using anatomically-detailed, 3D phenotypically-accurate models of SQT3 mutant I_{K1}.

Furthermore, possible similarities and/or differences in the atrial pro-arrhythmic mechanisms of Kir2.1 mutations due to their distinct effects on I_{K1} in the SQTS have not yet been characterised. The D172N and E299V mutations have distinct effects on currents carried by Kir2.1 channels; the D172 residue is involved in the steep, highly voltage-dependent portion of rectification [4,25,26], whereas E299 is involved in the shallow part of rectification which shows less voltage dependence [6,27]. The effects of the E299V mutation on Kir2.1 currents are more pervasive than are those of Kir2.1 D172N, as the E299V mutation significantly alters the membrane potential range over which I_{K1} is active, fundamentally changing its role in repolarisation of the AP. The comparative pro-arrhythmic effects of these two distinct mutations have yet to be elucidated.

This study aimed to evaluate and compare the influence of the SQT3 D172N and E299V Kir2.1 mutations on atrial repolarisation and susceptibility to re-entrant excitation, using heterogeneous, multi-scale models of human atrial electrophysiology. It also aimed to investigate the effects of simulated ion channel inhibition on SQT3-mediated AF, as this may provide theoretical insights into possible pharmacological approaches for the management of AF in SQT3.

Results

Model validation

I_{K1} formulations developed in wild-type (WT) and SQT3 mutant conditions accurately reproduced experimental I-V relationship data under voltage clamp conditions; namely (i) significantly increased outward currents at potentials in the range of −75 to −45 mV in D172N mutation conditions, and (ii) impaired inward rectification and increased outward I_{K1} at potentials above −55 mV associated with the E299V mutation, as shown in Fig 1. Furthermore, simulated AP clamps using the same digitised human atrial AP waveform as employed in previous *in vitro* experiments showed strong agreement with those obtained experimentally in WT and D172N conditions [26]. The Colman *et al.* (CZ) human atrial cell model updated with our WT I_{K1} formulation gave AP characteristics which were in excellent agreement with experimental data from human atrial myocytes (Supplemental S1 and S2 Figs). Incorporation of the modified family of CZ regional cell models into our 3D human atria geometry, as shown in Fig 2, gave a total activation time (AT) and conduction velocity (CV) in good agreement with experimental measurements (total AT 122 ms versus 116 ± 18 ms [28]; CV 0.71 ms^{-1} in the RA versus 0.7–0.9 ms^{-1} in RA free wall [29]).

Effects of SQT3 mutant I_{K1} on single cell atrial APs

Alterations to I_{K1} due to SQT3 mutations accelerated the repolarisation phase of action potentials in all conditions investigated, as shown in Fig 3A. The D172N mutation resulted in shortening of APD_{90} and hyperpolarisation of the resting membrane potential (RMP) (Fig 3Ai), with the homozygous condition exerting a more profound effect than the heterozygous condition. This was associated with an increased outward I_{K1} without altered rectification (see Fig 1A), providing a stronger repolarising current during terminal repolarisation (Fig 3Aii). In contrast, as the E299V mutation markedly altered (WT-E299V) or abolished (E299V) inward rectification of I_{K1} over the physiological range of membrane potentials (see Fig 1B), it resulted in a significant contribution of outward I_{K1} during the entire AP, suppressing the AP plateau

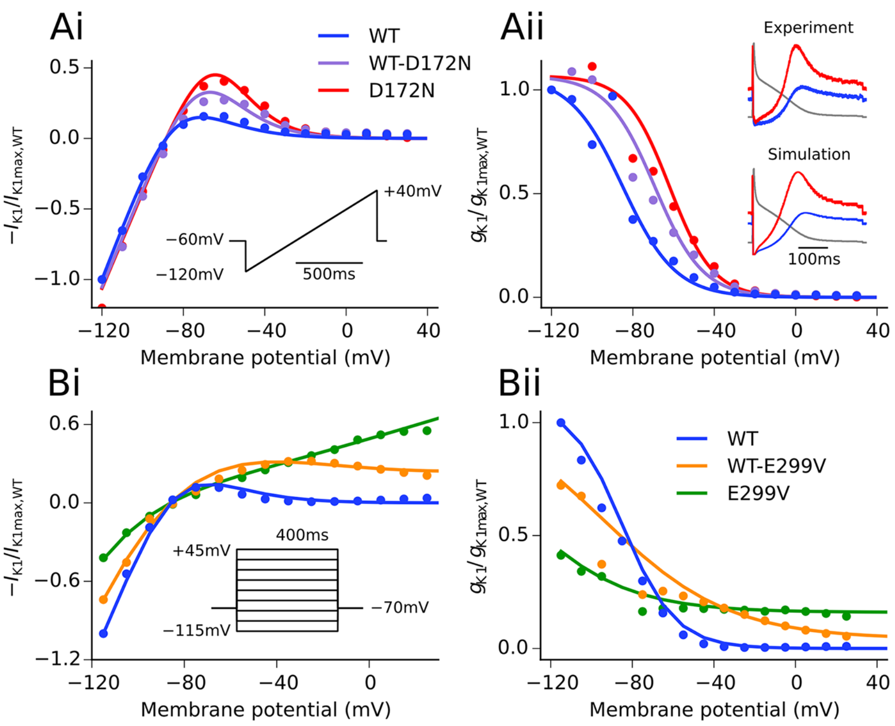

Fig 1. Experimental and simulated I-V and g_{K1}-V relationships. Comparison of model I-V relationship (Ai) and computed g_{K1}-V relationship (Aii) in WT, WT-D172N, and D172N conditions (solid lines) with the experimental data of El Harchi *et al.* [26] (points). Simulated AP clamps using a digitised AP waveform from the Nygren *et al.* human atrial cell model [30] as described in [26] are shown inset. Comparison of model I-V relationship (Bi) and computed g_{K1}-V relationship (Bii) in WT, WT-E299V, and E299V conditions (solid lines) with the experimental data of Deo *et al.* [6] (points). The voltage clamp protocol used in each case is shown inset in (i).

https://doi.org/10.1371/journal.pcbi.1005593.g001

and uniformly shortening the APD (Fig 3Ai and 3Aii). A summary of the effects of the studied mutations on AP characteristics at a pacing frequency of 1 Hz is shown in Table 1.

Fig 3 also shows secondary effects of mutant I_{K1} on I_{CaL} (Fig 3Aiii) and I_{Na} (Fig 3Aiv). The D172N mutation conditions affected the AP during terminal repolarisation; at this phase of the AP little inward current is passed through the L-type calcium channel, thus the profile of I_{CaL} was not significantly affected. The E299V mutation conditions, which reduced or abolished inward rectification of I_{K1} over physiological voltages, caused a decreased plateau potential of the AP, reducing I_{CaL} during this phase. The effect of mutations on the RMP altered the profile of I_{Na} in two ways; (i) hyperpolarised RMP as seen in the D172N conditions increased sodium current availability thus increasing peak current density, and (ii) time for activation of I_{Na} following stimulation increased as a function of the voltage difference between the RMP and threshold potential for AP initiation (or decreased in the case of the homozygous E299V mutation which was associated with more depolarised RMP). Whereas the WT-E299V mutation did not significantly affect the RMP, the homozygous E299V mutation condition led to a small depolarisation of the RMP, due to decreased outward currents in the region of ~70–80 mV. Elevation of the RMP, as seen for the E299V mutation, resulted in partial inactivation of sodium channels, decreasing I_{Na} amplitude (Fig 3Aiv), the action potential amplitude (APA) and maximum upstroke velocity (MUV) (see Table 1).

All mutation conditions investigated shortened the APD_{90} across a wide range of diastolic intervals (DIs) (Fig 3Bi). The maximal slope of APD restitution (Fig 3Bii) was decreased in all

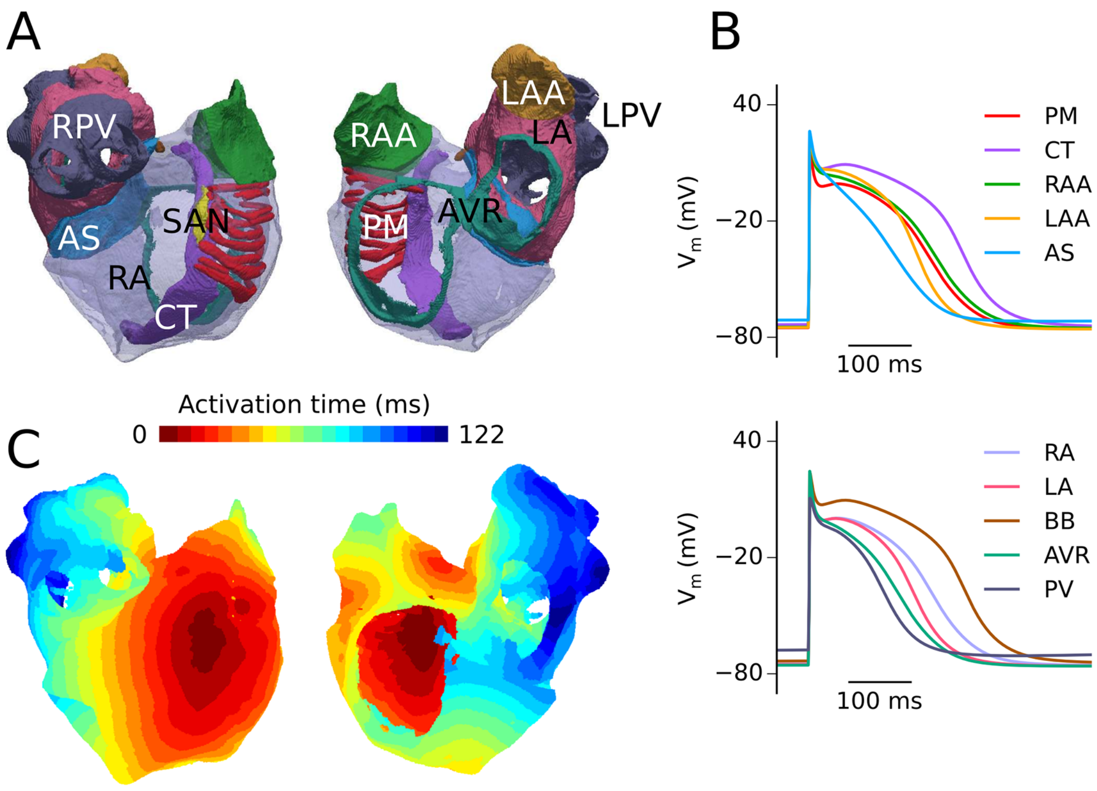

Fig 2. 3D anatomical atria model and regional APs. The anatomical atria geometry shown from two views–looking at the RA posterior wall and into the cavities (A), with regions of atrial septum (AS), atrio-ventricular ring (AVR), crista terminalis (CT), left atrium (LA), left atrial appendage (LAA), left pulmonary veins (LPV), pectinate muscles (PM), right atrium (RA), right atrial appendage (RAA), right pulmonary veins (RPV), and sinoatrial node (SAN) shown for reference. Action potentials for regional cell models including the Bachmann's bundle (BB) at 1 Hz (B), with colours corresponding to tissue segmentation in (A). Activation sequence in the heterogeneous 3D anatomical atria model, following initiation of an AP in the SAN region (C).

https://doi.org/10.1371/journal.pcbi.1005593.g002

SQT3 mutation conditions compared to the WT, with the homozygous E299V mutation in particular showing almost no rate adaptation. Whereas the APD measured in the homozygous D172N condition was longer than that in both heterozygous and homozygous E299V mutations at normal pacing rates, at fast pacing rates the homozygous D172N restitution curve crossed over the E299V curves.

Effects of SQT3 mutant I_{K1} on tissue APD distribution

Each regional cell model incorporated into the 3D virtual human atria has a different AP profile due to intrinsic different electrophysiological properties of the atria [19,22,31]. The spatial distribution of the action potential duration, ΔAPD, in coupled tissue is influenced by electrotonic coupling, with regional APD depending on region size and the APD of neighbouring regions. ΔAPD across the 3D human atria geometry at a pacing frequency of 1 Hz for all mutations is shown in Fig 4. The D172N mutation conditions showed only a modest decrease in global ΔAPD, whilst increasing APD heterogeneity at the CT/PM junction and preserving a high degree of heterogeneity at the PV/LA junction (summarised in S3 Fig). The E299V mutation conditions, however, caused a more marked decrease in ΔAPD, with the homozygous E299V mutant showing the most homogenous APD distribution (overall ΔAPD of 69 ms versus 120 ms in the WT case). Whereas ΔAPD at the CT/PM junction was increased for the

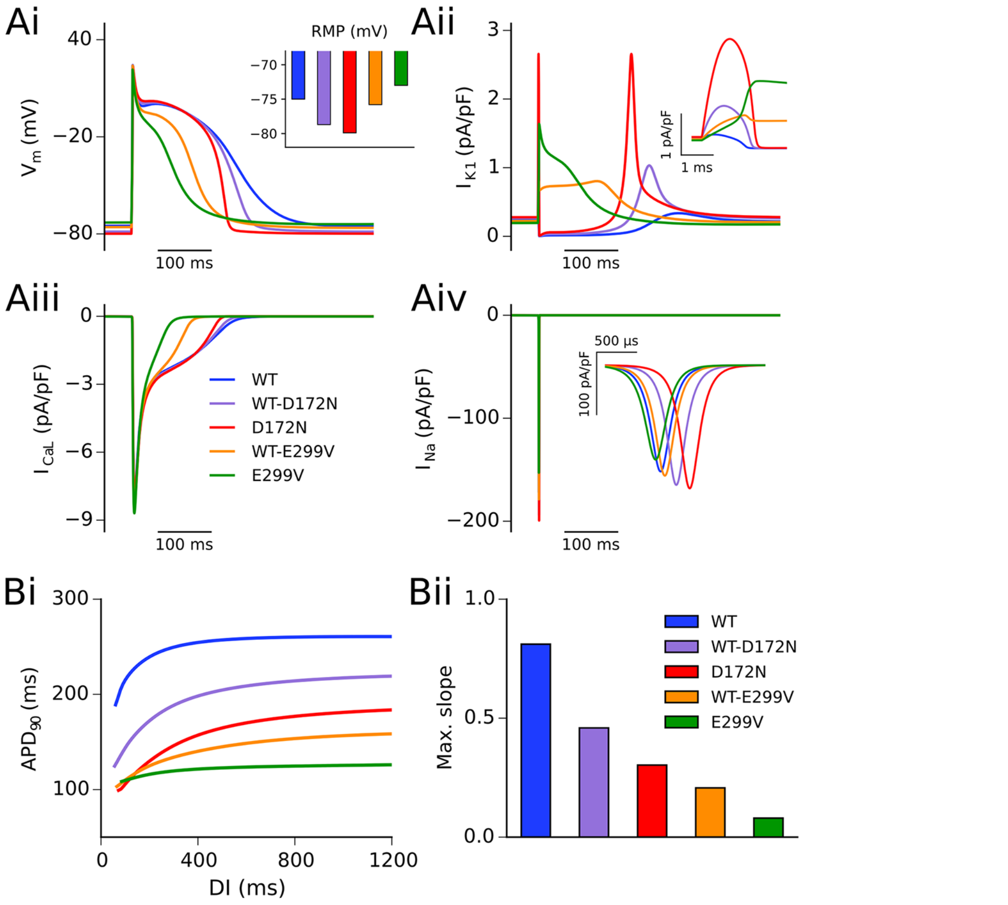

Fig 3. Simulated AP profiles and current traces in WT and mutation conditions. Action potential profiles in WT (blue), WT-D172N (mauve), D172N (red), WT-E299V (orange), and E299V (green) conditions at a pacing frequency of 1 Hz (Ai), with inset showing RMP. Corresponding current traces are shown for I_{K1} (Aii), I_{CaL} (Aiii), and I_{Na} (Aiv). Insets for panels (Aii) and (Aiv) show current profiles during the AP upstroke for I_{K1} and I_{Na}, respectively. (Bi) Restitution of the APD_{90} as determined using an S1-S2 protocol, and (Bii) maximal slope of restitution.

https://doi.org/10.1371/journal.pcbi.1005593.g003

E299V mutation conditions, there was almost no difference in APD at the PV/LA junction for the WT-E299V condition, and APs failed to propagate uniformly across the PV region in the E299V condition (conduction blocks shown in black). This was due to the combination of reduced CV and increased electrotonic suppression arising from differences in RMP from the

Table 1. AP properties in WT and SQT3 mutant conditions in the baseline single cell model.

	APA (mV)	RMP (mV)	APD_{50} (ms)	APD_{90} (ms)	MUV (V/s)
**WT**	99.2	-75.0	171.6	259.9	194.5
**WT-D172N**	103.0	-78.6	166.6	213.9	214.4
**D172N**	103.5	-79.9	154.7	177.5	200.5
**WT-E299V**	99.8	-75.8	99.4	154.4	199.4
**E299V**	94.5	-73.0	60.4	125.0	173.1

A summary of atrial AP characteristics: action potential amplitude (APA), resting membrane potential (RMP), action potential duration at 50% and 90% repolarisation (APD_{50} and APD_{90}, respectively), and maximum upstroke velocity (MUV) in the baseline single cell model at a pacing frequency of 1 Hz.

https://doi.org/10.1371/journal.pcbi.1005593.t001

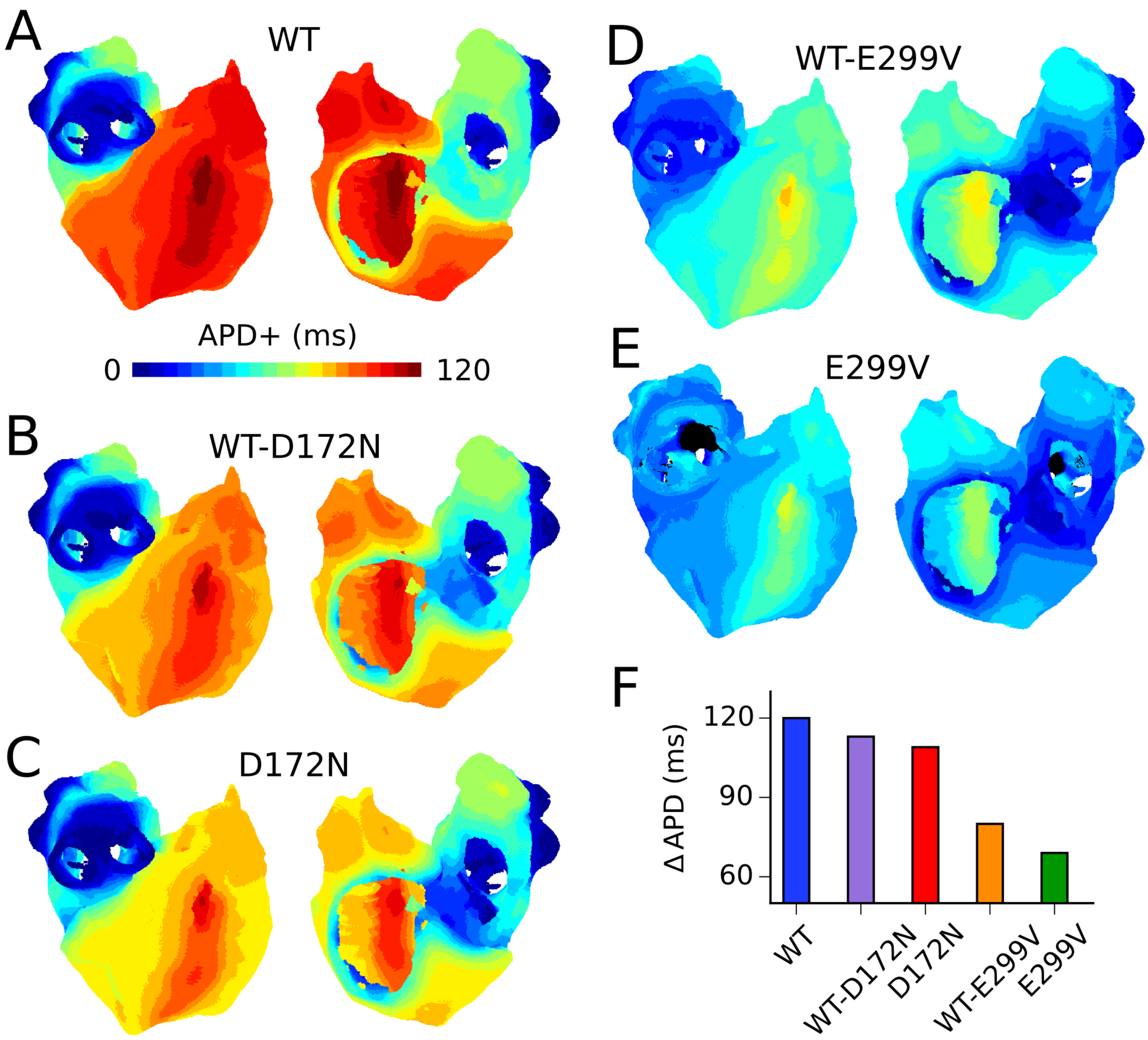

Fig 4. Spatial dispersion of APD in 3D anatomical atria model. APD distribution maps in WT (A), WT-D172N (B), D172N (C), WT-E299V (D), and E299V (E) mutation conditions, with corresponding global ΔAPD (F). The colour bar shows APD relative to the shortest APD measured in each condition, designated APD+. The scale of the colour bar is fixed at the value of ΔAPD in the WT condition (120 ms) to facilitate comparison between the mutation conditions. The colour black shows regions where membrane potentials failed to exceed a threshold value (−20 mV).

https://doi.org/10.1371/journal.pcbi.1005593.g004

surrounding LA region. Augmented regional differences at the CT/PM junction led to an increase in the temporal vulnerability to uni-directional conduction block in all mutation conditions (see S4 Fig).

Effects of SQT3 mutant I_{K1} on tissue restitution properties

Restitution curves for the conduction velocity (CV), effective refractory period (ERP), wavelength (WL) of re-entry, and tissue excitability measured in the 1D strand are shown in Fig 5. Alterations to CV restitution (Fig 5A) were mediated primarily by effects of the mutations on

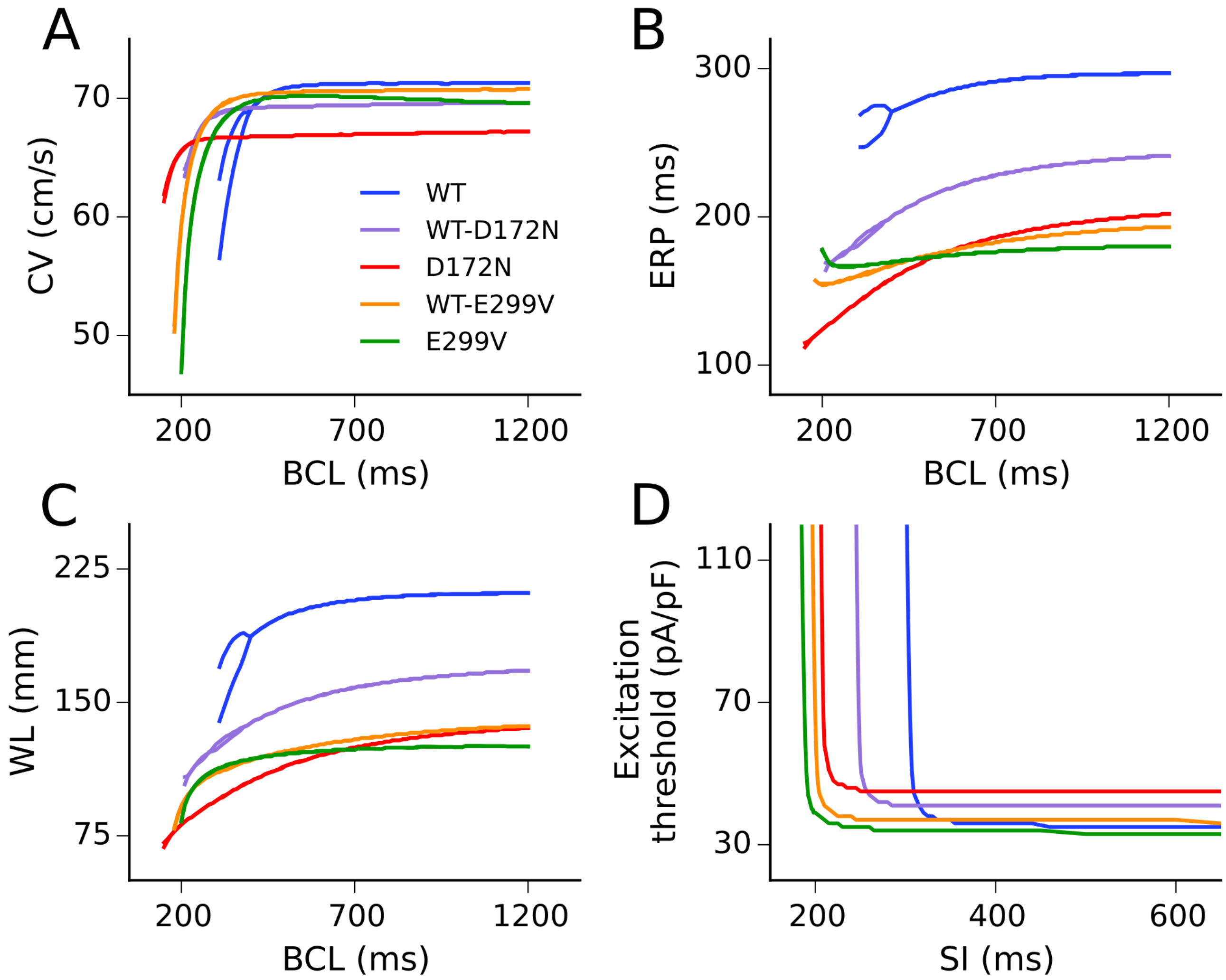

Fig 5. Tissue restitution properties in WT and mutation conditions. Restitution of the CV (A), ERP (B), and WL of re-entry (C) against a range of basic cycle lengths (BCL), and excitation threshold (D) against a range of S1-S2 stimulus intervals (SI), as measured in the 1D tissue model.

https://doi.org/10.1371/journal.pcbi.1005593.g005

the RMP. The D172N mutation conditions hyperpolarised the RMP; this resulted in increased sodium channel availability, outweighing any antagonistic effect of increased outward I_{K1} during membrane depolarisation and increasing single cell MUV (see Table 1) which might be expected to lead to an increase in CV. However, the increased voltage gradient between active and resting cells in tissue resulted in higher electrotonic suppression at the wavefront, causing an overall decrease in conduction velocity. The CV in the WT-E299V condition was decreased by similar mechanisms. For the homozygous E299V mutation condition, the voltage gradient between active and resting cells was reduced, yet the combination of depolarised RMP (which decreases sodium channel availability) and increased outward currents during membrane depolarisation led to a more significant reduction in CV than for the heterozygous mutant form.

The ERP was shortened in all mutation conditions compared to the WT, with the homozygous E299V condition showing the least rate adaptation (Fig 5B). ERP restitution in the E299V and WT-E299V mutation conditions showed a deflection to higher values at fast rates, with minima at basic cycle lengths (BCL) of ~270 ms and ~200 ms, respectively. As with the single

cell APD restitution, the D172N ERP restitution curve crossed those of the E299V mutation conditions at fast rates. The WL of re-entry was shortened for all SQT3 mutants across the range of DIs investigated (Fig 5C). AP alternans arising from fast rate pacing were reduced in all mutation conditions, consistent with a decrease in the steepness of restitution.

The rate-dependent effect of the SQT3 mutations on tissue excitability, measured as excitation threshold, is shown in Fig 5D. At low rates the D172N mutation conditions decreased tissue excitability, the WT-E299V mutation did not significantly affect excitability, and the homozygous E299V mutation increased excitability. At high rates, all mutations investigated increased excitability compared to the WT. For example, at a stimulus interval (SI) of 300 ms the excitation threshold for the WT tissue was 169 pA/pF compared with 41 pA/pF for WT-D172N, 45 pA/pF for D172N, 37 pA/pF for WT-E299V, and 34 pA/pF for E299V mutant tissue. Differences in excitation threshold between mutation conditions can be explained by how the mutations affected the RMP, i.e. mutations which hyperpolarised the RMP increased the voltage gradient between the RMP and the threshold for AP firing, thus larger stimulus currents were required to elicit APs which propagate in tissue. The SI at which the excitation threshold began to tend towards an infinite value is determined by the degree of APD shortening in each of the mutation conditions.

Characterisation of spiral wave dynamics in 2D due to SQT3 mutant I_{K1}

Further simulations were performed using an idealised area of 2D tissue to investigate the functional impacts of the SQT3 mutations on the dynamic behaviours of re-entrant excitation waves, including the lifespan, stability and meandering pattern of the re-entry (see S5 and S6 Figs). In the WT condition, re-entrant waves had an average lifespan of <300 ms, as the tip of the re-entry consistently meandered out of the tissue boundaries. As for the WT-D172N mutation condition, re-entry sustained (lasted for the 5.0 s duration of the simulation) in 3/5 simulations, with spiral waves drifting unpredictably and following relatively linear trajectories (see S5 Fig) [32]. In the homozygous D172N mutation condition, sustained re-entry occurred in 5/5 simulations. When the S2 stimulus was applied early after the ERP, the emergent spiral wave interacted with the refractory tail causing transient wave break and meander, before settling into a stationary, tightly-clustered hyper-meandering trajectory [32].

In the WT-E299V condition, re-entry sustained in 5/5 simulations. However, this mutation condition was the most prone to wave break, due to drift-induced differences in refractoriness across the 2D tissue patch and a high frequency of rotation. The homozygous E299V mutation produced sustained re-entry in 3/5 simulations. This mutation produced spiral waves which rotated with a slower dominant frequency (DF) than the heterozygous form of the mutation (see S3 Table). Spiral wave trajectories in the E299V condition drifted significantly and showed distinctive 'outward petals', also known as a hypocycloidal meander pattern [32].

The temporal evolution of spiral wave core trajectories in 2D tissue is shown in S5 Fig for all mutation conditions and the average lifespan of spiral waves and area of meander in time are summarised in S6 Fig. Whereas both the D172N and WT-E299V mutation conditions sustained in all simulations, spiral waves in the WT-E299V condition meandered over a much larger area on average (representative simulations shown in S1–S5 Videos). The average lifespan of spiral waves in the homozygous E299V mutation condition was shorter than in the heterozygous condition due to increased tissue excitability, which led to a more unstable form of re-entry.

Arrhythmia simulations in the 3D virtual human atria

The results of 3D simulations, where re-entrant scroll waves were initiated using the phase distribution method, are summarised in Fig 6. Re-entrant excitation waves sustained for ~3.3 s in

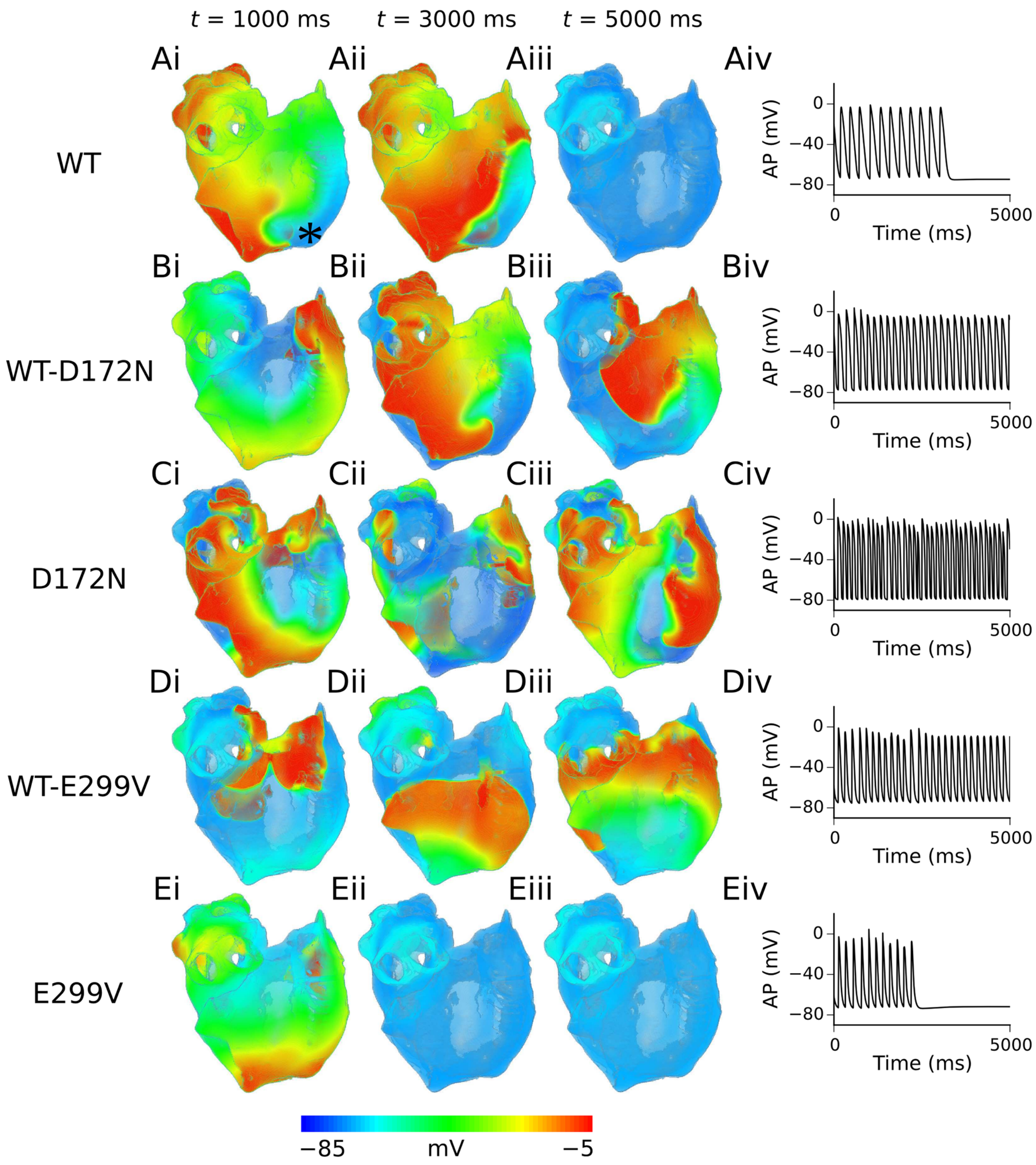

Fig 6. Scroll waves and corresponding APs in 3D virtual human atria. Evolution of scroll waves following initiation of re-entry, from time $t = 1000$ ms (i) to $t = 3000$ ms (ii) to $t = 5000$ ms (iii) in WT (A), WT-D172N (B), D172N (C), WT-E299V (D), and E299V (E) conditions, with corresponding APs extracted from the RA (recording location is marked with an asterisk in panel Ai) (iv). The viewpoint is looking at the RA posterior wall.

https://doi.org/10.1371/journal.pcbi.1005593.g006

the WT condition, before self-terminating (S6 Video). In the heterozygous and homozygous D172N mutation conditions, re-entrant scroll waves were sustained for the 5.0 s duration of the simulation, with co-existence of multiple wavelets in the homozygous D172N mutation condition (S7 and S8 Videos). In the WT-E299V mutation condition re-entry was also sustained for the duration of the simulation, though scroll waves meandered significantly for ~2.0

Table 2. A summary of dominant frequency and lifespan in 3D simulations.

	WT	WT-D172N	D172N	WT-E299V	E299V
**DF (Hz)**	N/A	5.2	7.5	5.3	N/A
**Lifespan re-entry (s)**	3.3	5.0 (sustained)	5.0 (sustained)	5.0 (sustained)	2.4

Dominant frequency (DF) was calculated for all continuous time series of APs.

https://doi.org/10.1371/journal.pcbi.1005593.t002

s before settling into a stable, anatomical re-entry around the opening of the inferior vena cava (S9 Video). In the homozygous E299V condition, the initiated scroll wave meandered over a large area, before ultimately colliding with its own refractory tail and self-terminating at ~2.4 s (S10 Video). The DF and lifespan of re-entry, where applicable, are summarised in Table 2. It should be noted that while SQT3 mutation conditions favoured development of sustained re-entry, the resulting patterns were relatively organized and did not cause chaotic fibrillatory behaviour as seen in atrial fibrillation.

‘Pharmacological’ investigations in the 3D virtual human atria

The effects of all drug interactions simulated on APD prolongation and dispersion (ΔAPD) at 1 Hz are summarised in S7 Fig. All reductions to potassium channel conductances investigated in the WT-D172N condition prolonged the APD to some extent, whilst also restoring ΔAPD to roughly the same level seen in the WT condition (see S8 Fig). Agonism of the L-type calcium channel prolonged APD, however, it also resulted in a significant increase in ΔAPD. In the WT-E299V condition, inhibition of potassium channels prolonged the APD but only caused a modest increase in ΔAPD. Individual or combined channel blocks involving I_{K1} resulted in incomplete excitation of the PV region. A 100% increase in I_{CaL} was effective in prolonging APD and restoring ΔAPD; however, excessive agonism of the L-type calcium channel (such as 250% in our simulations) was shown to hugely increase ΔAPD beyond the WT level, similar to the situation observed in the WT-D172N mutation condition.

Snapshots of simulated pharmacological modulation of re-entry in WT-D172N and WT-E299V conditions are shown in Fig 7, with corresponding localised atrial excitations extracted from the RA. 50% individual reductions of I_{K1} and I_{Kr} alone were insufficient to terminate re-entry in the WT-D172N condition; although both caused a decrease in the DF (I_{K1} block produced a more significant decrease in DF–Table 3). However, combined 50% block of I_{K1} and I_{Kr} resulted in self-termination of the re-entrant wave after ~3.1 s, as shown in Fig 7. A 50% reduction in the atrial-specific I_{Kur} exhibited only a very mild anti-arrhythmic effect, reducing the DF from 5.2 Hz to 5.0 Hz but failing to terminate re-entry. When this was paired with a 50% reduction in I_{Kr}, however, re-entrant excitation waves self-terminated after ~0.5 s (S11 Video).

A 100% increase in I_{CaL} in the WT-D172N condition significantly reduced the DF of re-entry, from 5.2 Hz to 4.2 Hz, yet was insufficient to terminate re-entrant excitation waves. Increasing I_{CaL} by 250% resulted in transient re-entrant excitations which self-terminated after ~0.6 s.

The combination of 50% I_{K1}+I_{Kr} reduction was successful in preventing sustained re-entry in the WT-E299V condition, as well as 50% I_{K1} reduction alone. However, measurements of the APD dispersion at a normal pacing rate showed that reducing I_{K1} in the WT-E299V condition precluded complete excitation of the PV region (S7 Fig). 50% reduction of I_{Kr}, on the other hand, was also sufficient to terminate re-entry (Fig 7) whilst maintaining excitability across the entire human atria.

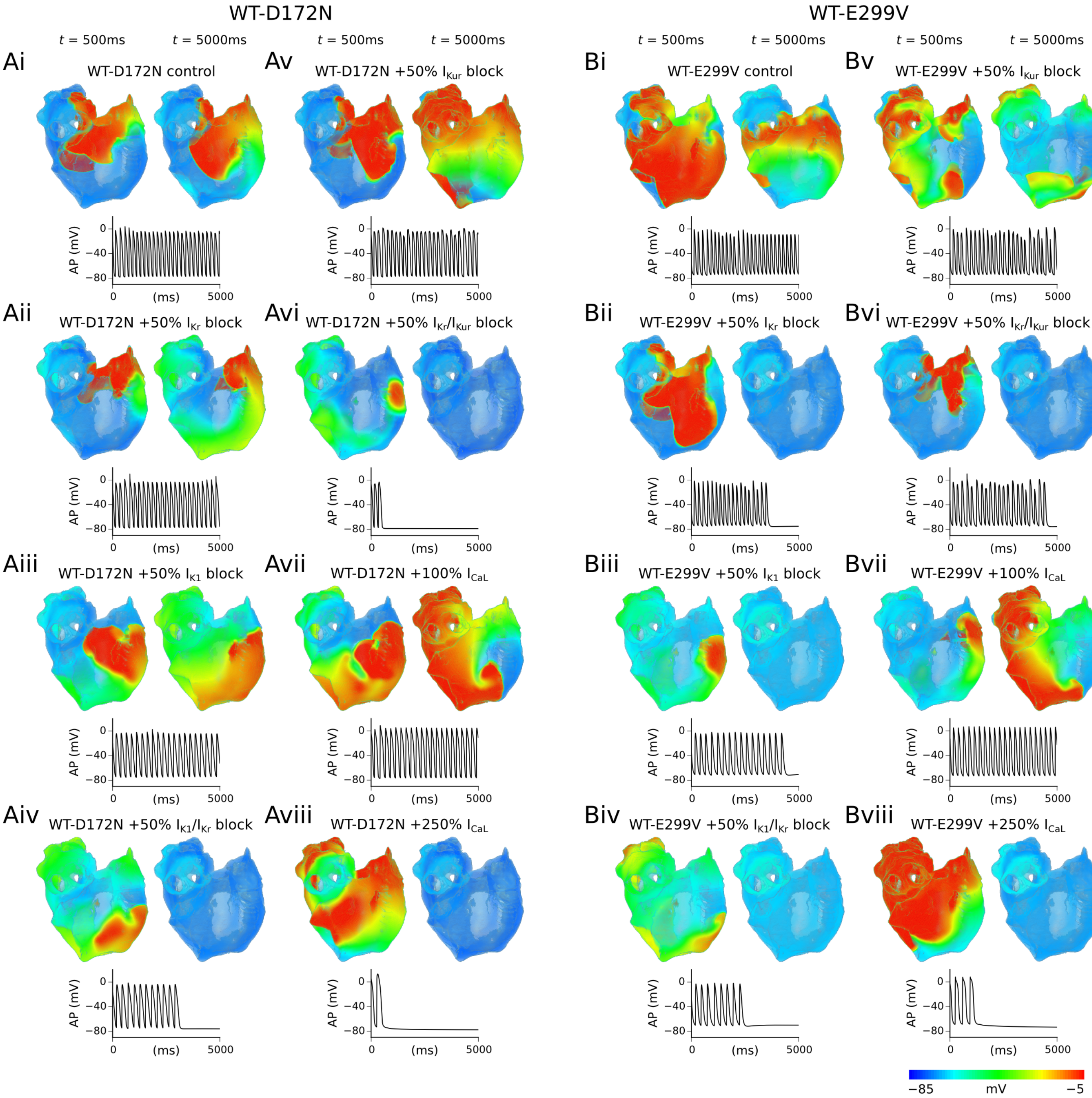

Fig 7. Simulated drug effects in 3D virtual human atria. Snapshots of simulated drug effects on re-entrant scroll waves in WT-D172N (A) and WT-E299V (B) conditions. For both mutation conditions panels correspond to the following cases: control (i), 50% I_{Kr} block (ii), 50% I_{K1} block (iii), combined 50% I_{K1}+I_{Kr} block (iv), 50% I_{Kur} block (v), combined 50% I_{Kr}+I_{Kur} block (vi), 100% increase in I_{CaL} (vii), and 250% increase in I_{CaL} (viii). For each case, two columns are shown which correspond to $t = 500$ ms and $t = 5000$ ms after initiation of re-entry, and corresponding APs extracted from the RA are shown below.

https://doi.org/10.1371/journal.pcbi.1005593.g007

As in the WT-D172N condition, 50% reduction of I_{Kur} alone in the WT-E299V mutation condition demonstrated a mild anti-arrhythmic effect, causing a small decrease in DF from 5.3 Hz to 4.8 Hz which, when combined with 50% block of I_{Kr}, was sufficient to terminate re-

Table 3. A summary of simulated drug effects in the WT-D172N and WT-E299V conditions.

	WT-D172N		WT-E299V	
	**DF (Hz)**	**Lifespan re-entry (s)**	**DF (Hz)**	**Lifespan re-entry (s)**
**Control**	5.2	5.0 (sustained)	5.3	5.0 (sustained)
**+50% I_{K1} block**	4.0	5.0 (sustained)	N/A	4.4
**+50% I_{Kr} block**	4.8	5.0 (sustained)	N/A	3.6
**+50% I_{K1}/I_{Kr} block**	N/A	3.1	N/A	2.5
**+50% I_{Kur} block**	5.0	5.0 (sustained)	4.8	5.0 (sustained)
**+50% I_{Kr}/I_{Kur} block**	N/A	0.5	N/A	4.5
**+100% I_{CaL}**	4.2	5.0 (sustained)	4.2	5.0 (sustained)
**+250% I_{CaL}**	N/A	0.6	N/A	1.5

Averaged dominant frequency (DF) and lifespan of re-entry were computed from time series of APs from several locations on the 3D human atria geometry recorded during re-entry simulations.

https://doi.org/10.1371/journal.pcbi.1005593.t003

entry (S12 Video). Simulated calcium channel agonism in the WT-E299V condition also yielded similar results to the WT-D172N mutation; increasing I_{CaL} by 100% significantly reduced the DF of re-entry but was insufficient to cause termination of re-entry, whereas a 250% increase in I_{CaL} rapidly terminated re-entry. Summaries of all pharmacological investigations in the human atria in WT-D172N and WT-E299V conditions are presented in Table 3.

Comparison with an alternative human atria model

Model-specificity of results was investigated by conducting further simulations using the Grandi *et al.* (GB) model of the human atrial AP [33]. The GB model was previously employed by Deo *et al.* in order to demonstrate human atrial APD shortening, loss of rate-adaptation of the APD, and increased vulnerability to re-entry in homogeneous tissue associated with the E299V-Kir2.1 mutation [6]. The APD shortening observed in our study in E299V mutation conditions using the GB model is consistent with that observed in the study of Deo *et al.* [6], and is qualitatively similar with our simulation results using the CZ model (see S10A Fig). Results with the two models were also similar for the D172N mutation, although this caused a larger relative degree of APD shortening in the GB model than in the CZ model, as the GB model AP exhibits a slow repolarisation tail and lack of prominent plateau phase, showing greater sensitivity to changes in repolarising I_{K1}. Changes to single cell properties such as APA, RMP, and MUV observed in the GB model mirrored those found in the CZ model (see S4 Table).

The maximal slope of APD restitution was decreased in all SQT3 mutation conditions compared to the WT, as observed in the CZ model (see S10B Fig). In addition, global dispersion of APD was decreased more significantly by the E299V mutation than the D172N mutation in the GB model, reproducing another finding in the CZ model (S11 Fig). These findings are reassuring; changes to tissue excitability and stability of re-entry in SQT3 mutant I_{K1} conditions identified in the CZ model were mediated by the RMP, APD, and dispersion of APD–alterations to all of which were reproduced using an alternative, well-established human atrial cell model [33].

Discussion

The 3D virtual human atrium is a simulation tool for the clinical study of excitation waves in the human atria in sinus rhythm and AF [23,34,35], and has been employed in several of our previous studies [23,34,36]. This study builds on previous *in silico* work [6,37,38] on

investigating the substrate for AF in potassium channel-linked SQTS by utilising multi-scale idealised atrial tissue models as well as the 3D virtual human atrium, which incorporates heterogeneous atrial electrophysiology in an anatomically-realistic setting [23,34]. This study is the first to our knowledge to characterise *in silico* the effects of genetic mutations associated with *any* form of the SQTS with inclusion of intrinsic regional differences in cellular electrophysiology and anatomical details in the human atria. Furthermore, we present a theoretical exploration of pharmacological interventions for atrial arrhythmias in SQT3, which has potential clinical relevance.

Main findings

Our major findings are as follows. (1) Heterozygous and homozygous D172N and E299V mutations shortened the APD through different mechanisms–the D172N mutations through increased outward I_{K1} during the terminal phase of repolarisation and the E299V mutations through increased I_{K1} at depolarised membrane potentials throughout the AP. (2) SQT3 mutations shortened the wavelength of re-entrant excitation waves through a reduction in both ERP and CV, which is mediated primarily by effects on the RMP that affects tissue excitability. (3) A higher degree of APD dispersion was preserved in the D172N mutations than E299V mutations, with co-existence of short APDs and larger regional APD differences leading to a more stable form of re-entry. (4) Pharmacological modulation of AF in SQT3 showed some differences between D172N and E299V mutations. However, combined I_{Kr} and I_{Kur} block emerged as a safe pharmacological option in both forms of SQT3 in our simulations.

Pro-arrhythmic effects of SQT3 mutant I_{K1}

Up-regulation of I_{K1} is an unequivocal finding in ionic remodelling of the human atrial AP associated with AF [11–14]. Computational studies of human atrial cells have suggested this to be the primary determinant of APD shortening underlying self-perpetuation of AF [15], as well as an important determinant of rotor stability and frequency in tissue [16]. An *in silico* investigation of the familial AF V93I-Kir2.1 mutation to I_{K1} by Kharche *et al.* [17] showed that both heterozygous and homozygous forms of the mutation (which increased both inward and outward I_{K1}) shortened APD, hyperpolarised RMP, flattened restitution curves of APD, ERP, and CV, and stabilised rotors in the 3D virtual human atria. Unlike the present investigation, however, that study [17] lacked incorporation of heterogeneity of human atrial electrophysiology, which has been found to be an intrinsic characteristic of the atria [19–22].

In this study, differences in the I_{K1} I-V relationship caused by the SQT3 mutations were investigated, shown in Fig 1, manifesting differences in the way the mutations affected the AP. The SQT3 proband identified in [4], who was heterozygous for the D172N Kir2.1 mutation, presented with a history of presyncope and palpitations, which are often caused by supraventricular tachycardias originating in the atria [39]. In our simulations, the D172N mutation decreased overall ΔAPD by only a small degree in the 3D virtual human atria, whilst preserving a high degree of heterogeneity at the PV/LA junction, and increasing ΔAPD at the CT/PM junction (S3 Fig). It has previously been shown that short APDs co-existing with regional differences in APD creates a substrate favourable to the sustenance of re-entry in human [23] and canine [24] AF remodelled atria. Our results are consistent with this finding, as both the heterozygous and homozygous D172N mutation conditions were capable of sustaining re-entry in the 3D virtual human atria. In our previous study of SQT3 and ventricular repolarisation, the D172N-Kir2.1 mutations were shown to significantly shorten APD and QT interval in a computational model of human ventricular myocytes, accelerating and stabilising re-entrant excitation waves in the human ventricles [40]. As in the present study, electrical

heterogeneities were shown to play an important role in the arrhythmia substrate, as augmented transmural dispersion of APD at localised regions of the ventricles led to increased temporal vulnerability to the genesis of uni-directional conduction block and spatial vulnerability to the initiation and sustenance of re-entry.

The E299V mutation differed from the D172N mutation in that it shortened the AP by increasing outward I_{K1} throughout the duration of the AP, not just during terminal repolarisation. In the heterozygous form of the mutation, this manifested itself as a small hyperpolarisation of the RMP, reduced APA and MUV, and dramatically shortened APD_{90}. These findings are in qualitative agreement with a recent study which investigated the effects of injecting virtual 'wild-type' I_{K1}, 'loss-of-function' I_{K1} associated with Andersen-Tawil syndrome, and 'gain-of-function' heterozygous E299V mutant I_{K1} via dynamic clamp on the action potential profile of human induced pluripotent stem cell derived cardiomyocytes [41].

The SQT3 proband in [6], who was heterozygous for the E299V-Kir2.1 mutation, presented with multiple episodes of paroxysmal AF. In our simulations the E299V mutation conditions significantly reduced APD heterogeneity across the human atria, whilst affecting heterogeneity at critical junctions differentially. Whereas the profound loss of overall ΔAPD in the pure E299V condition resulted in only transient re-entrant wave behaviour in 3D simulations, the intermediate loss of APD heterogeneity combined with abbreviated APD resulted in sustained re-entry in the WT-E299V condition. Interestingly, in our 2D simulations which were electrically-homogeneous, re-entry sustained in 3/5 simulations in E299V mutant tissue and the area of meander was comparable to the WT-E299V mutation condition (see S6 Fig), suggesting APD heterogeneity played an important role in re-entry dynamics in the 3D anatomical atria. The difference in effects of the homozygous E299V mutant conditions in 2D and 3D simulations underscores the value of conducting simulations at both levels. The long term behaviour of scroll waves in the 3D virtual human atria was not investigated; it can, however, be speculated that the combination of significant drift and reduced APD heterogeneity decreases the long-term stability of re-entrant circuits in tissue which increases the likelihood of eventual self-termination of re-entrant activity, mimicking the paroxysmal AF of the proband in [6]. This is different to the more stable form of re-entry predicted for the D172N mutation conditions (especially homozygous D172N which showed stationary meander patterns–see S5 Fig and S3 Video).

Not only does a large APD dispersion facilitate the sustenance of re-entrant excitations as suggested by our simulations, but significant regional differences in APD facilitate the *genesis* of re-entrant excitations, as temporal vulnerability of tissue to uni-directional conduction block is increased. This is consistent with prior experimental evidence that large dispersion of APD in the atria increases susceptibility to re-entry through local conduction blocks [42,43]. This suggests that large regional differences in ΔAPD associated with the SQT3 mutations increase the susceptibility to the initiation of re-entrant arrhythmias. This was confirmed in supplementary simulations by an increase in the temporal window of vulnerability to uni-directional conduction block at the CT/PM junction associated with all SQT3 mutation conditions, as shown in S4 Fig.

In addition to multiple pro-arrhythmic effects identified in the SQT3 mutations, both the D172N and E299V mutations were shown to flatten the restitution of APD, which may have a mixed anti- and pro-arrhythmic effect. Whereas steep restitution slopes facilitate alternans at the cellular level leading to wave-break in tissue, flattened restitution curves represent a loss of rate-adaptation, which can facilitate maintenance of high frequency excitation waves [17,36,44]. In the case of the D172N mutation, this finding is at variance with that reported in our previous study of human ventricles, in which simulated restitution curves were steepened [40]. The reason for this difference is not completely clear. It is likely due to differences in the

models used, as this study used an atrial model and our previous study used a ventricle model [40]. It is known that there are atrio-ventricular differences in the relative roles of I_{K1}, i.e. I_{K1} has a much larger current density in the ventricles [2,45], which is likely to affect responses of the models to 'gain-in-function' mutations to I_{K1}. By way of further comparison, in our previous study of a *KCNJ2* mutation associated with familial AF, both the Kir2.1 V93I mutation which increases outward I_{K1} and a simple linear increase in maximal conductance of I_{K1} were shown to flatten APD and ERP restitution curves [17], consistent with observations in the present study.

Pharmacological effects on re-entry in 3D virtual human atria

Use of the 3D virtual human atria in this study was extended in order to glean some insight into potential pharmacological targets in the treatment of SQT3-mediated AF. To date, no specific blockers of I_{K1} are in clinical use. In the case of the WT-D172N mutation, combined 50% reduction of I_{K1} and I_{Kr} was shown to be superior to 50% reduction of either I_{K1} or I_{Kr} alone in terminating re-entry. Chloroquine, which has been shown to be an effective inhibitor of D172N mutant Kir2.1 channels [26,46,47], blocks Kir2.1 and hERG channels at overlapping concentrations [26]. The results of our modelling thus provide evidence that a drug such as chloroquine, which inhibits both I_{K1} and I_{Kr}, may be effective in treating AF associated with the D172N mutation. Previous modelling studies have highlighted the potential of chloroquine to normalize ventricular repolarisation in D172N Kir2.1-linked SQT3 and to terminate rotors in paroxysmal and chronic AF, which are associated with increased I_{K1} [47,48]. Our findings are also consistent with a study by Noujaim *et al.* [49], in which chloroquine was shown to successfully terminate re-entry associated with up-regulated Kir2.1.

In the case of the WT-E299V mutation condition, the combination of 50% block of I_{K1} and I_{Kr} was effective in preventing sustained re-entry, suggesting that an agent combining these actions may also be effective in this form of SQT3. However, I_{K1} block in the WT-E299V condition, either alone or combined with I_{Kr} block, was also shown to cause incomplete excitation of the pulmonary vein region at a normal pacing rate. This could potentially result in further abnormalities of conduction or provide a re-entrant circuit for excitation waves around the partially inexcitable pulmonary veins, suggesting I_{K1} blockers may be unsuitable for management of AF mediated by the E299V-Kir2.1 mutation.

A 50% reduction in I_{Kr} alone was also sufficient to terminate re-entry in the WT-E299V mutation condition. At the single-cell level 50% I_{Kr} block had a relatively modest effect on the baseline APD (S7 Fig). This finding could, therefore, be a consequence of the inherently unstable nature of re-entry associated with the WT-E299V mutation, which is associated with paroxysms of AF [6]. Measurement of APD dispersion at 1 Hz (S7 Fig) revealed that the PV region was affected quite significantly by blocking I_{Kr} (this region has higher expression of I_{Kr} [20,21]), potentially de-stabilising circuits around this region. Again, this highlights the usefulness of conducting multi-scale simulations, as changes to the APD at the single cell level may not necessarily be a good predictor of drug effects in electrically-heterogeneous, realistic geometries [24]. Notably, the SQT3 proband in [6] was given amiodarone, which exerts its class III anti-arrhythmic action predominantly through block of I_{Kr} [50], to effectively control AF.

Whereas a selective reduction in I_{Kur} was unable to terminate re-entrant excitation waves, combined block of I_{Kr} and I_{Kur} was shown to be effective in terminating re-entry in both WT-D172N and WT-E299V mutation conditions. Combined inhibition of I_{Kr} and I_{Kur} has previously been shown to cause a synergistic increase in APD in a mathematical model of AF-remodelled human atrial cells [51]. As I_{Kur} is not expressed in ventricles, this combination would be expected to exert a greater APD prolongation in the atria, making it more of an

atrial-specific preventative measure against AF, which was the primary clinical presentation of the proband identified in [6].

Deo *et al.* [6], who first identified the E299V mutation in KCNJ2, suggested that an I_{CaL} activator of the BayK 8644 type may have some therapeutic value in this form of SQT3. We tested this hypothesis in the 3D virtual human atria by increasing maximal conductance of I_{CaL}. A 100% increase in I_{CaL} significantly reduced the DF of re-entrant AP waveforms, from 5.3 Hz to 4.2 Hz in the WT-E299V condition. A 200% increase in I_{CaL} further reduced the DF to 3.8 Hz, yet was still insufficient to terminate re-entrant wave behaviour. We found that the increase in maximal I_{CaL} conductance necessary to terminate re-entry in the WT-E299V condition (250% in our simulations) significantly depolarised the RMP, reduced MUV, and significantly increased global APD dispersion in the human atria (S8 Fig). Deo *et al.* reported that increasing I_{CaL} to the level necessary to restore the APD produced unphysiologically large calcium transients in a model of human ventricular myocytes [6], which is likely to significantly increase hypertensive risk. Furthermore, such a large increase in inward calcium currents could promote a torsadogenic effect in the ventricles. Effects of simulated I_{CaL} agonists in the WT-D172N condition were qualitatively similar to those obtained in the WT-E299V mutation condition in that a high degree of I_{CaL} agonism demonstrated anti-arrhythmic effects but profoundly increased dispersion of APD across the human atria. Our simulations thus provide further evidence that I_{CaL} agonists may be undesirable therapeutic candidates for AF mediated by both forms of SQT3 investigated.

Limitations

Limitations of the CZ model and 3D virtual human atria, such as the validation of regional cell models using primarily canine data and lack of a complete tissue fibre structure, have been discussed in detail elsewhere [23,34]. This study aimed to investigate arrhythmia substrates arising from purely cellular electrophysiological differences in SQT3 *KCNJ2* mutations, and as such did not consider effects of electrical or intracellular gap junction remodelling or fibrosis. Evidence of electrical and structural remodelling in SQT3 patients is currently lacking; nevertheless, these factors may contribute to the arrhythmia substrate [23,52] and give rise to multiple wavelets characteristic of AF. A further limitation may come from the fact that cardiac mechanical contraction was not considered, which, for ventricular function at least, has previously been suggested to be affected in SQTS patients [53–55], and may affect re-entrant wave dynamics [56,57].

The I_{K1} formulations in this study are based on current recordings from Kir2.1 channels. The WT I_{K1} formulation recapitulated the most salient difference between human atrial and ventricular I_{K1}, i.e. the vastly reduced maximal current density [2,45] (compared with our previous study in human ventricles [40]), whilst retaining a high degree of similarity in kinetics with leading human atria models [30,33,58] (see S12 Fig). Native I_{K1} is carried by homo- or heteromeric assemblies of each of the Kir2.x (Kir2.1, Kir2.2, Kir2.3) subunits expressed in cardiac myocytes [59]. Human atrial I_{K1} has been reported to show weaker rectification than its ventricular counterpart [45], suggesting the possibility of a more prominent than in ventricle role for the Kir2.3 isoform [59]. However, whereas the Kir2.3 protein has been shown to be more abundant in human atrium than ventricle (10% versus <1%), the Kir2.1 protein was nevertheless measured to have the highest relative abundance amongst Kir2.x isoforms in human atrium (81% versus 92% in ventricle) [2]. Interestingly, a recent study found that altering the I-V profile of I_{K1} to resemble Kir2.3 in mathematical tissue models of the human atrial PV/LA junction did not affect the role that I_{K1} played in determining gradients of sodium availability, MDP, and MUV [60] which dictate drift direction; this suggests that any alteration to re-entry

dynamics by incorporation of a Kir2.3-like component would be minimal, and thus unlikely to change the fundamental conclusions drawn in our study.

Conclusions

The findings of this study add to the growing weight of evidence implicating increased I_{K1} in increased susceptibility to the initiation and maintenance of AF, and further highlight the influence of regional heterogeneities in overall response to gain-of-function mutations that increase I_{K1}. Functional differences exist between the effects of SQT3-related D172N and E299V mutations to I_{K1} on human atrial electrophysiology: distinct mechanisms of APD shortening between D172N and E299V mutants at the single cell level manifested themselves as differences in how the mutations affected APD dispersion in the human atria and influenced re-entry dynamics. Heterozygous and homozygous forms of the D172N mutation resulted in co-existence of short APDs with marked regional heterogeneities which facilitated maintenance of re-entrant excitation waves in the human atria. In contrast, the E299V mutation presented with more unstable re-entrant wave behaviour due to significantly reduced global APD dispersion. Combined reduction of I_{K1} and I_{Kr}, mimicking the effects of chloroquine, was established as an effective inhibitor of WT-D172N mutant I_{K1} in 3D re-entry simulations, whereas class III anti-arrhythmic agents which block I_{Kr} may offer a safer alternative to I_{K1} block in the WT-E299V mutation. In both SQT3 mutation conditions, I_{Kr} and I_{Kur} block combined synergistically to increase APD and terminate re-entrant wave behaviour.

Methods

I_{K1} and human atrial cell models

A contemporary human atrial cell model developed by Colman *et al.* [23] (CZ model) was used for the simulations in this study. The original equations for I_{K1} in the CZ model are native to the parent Courtemanche-Ramirez-Nattel (CRN) model [58]. In this study, a WT formulation of I_{K1} was developed based on WT $I_{Kir2.1}$ currents recorded in Chinese Hamster Ovary cells at physiological temperature [26], and the maximal conductance chosen in order to give AP characteristics such as APD and RMP within the experimental range measured for human atrial myocytes (S1A Fig). I_{K1} formulations for heterozygous (WT-D172N) and homozygous (D172N) SQT3 mutants were also developed based on fitting voltage clamp data from [26], and validated using AP clamp data [26]. The waveform used, which is the same as in [26], had small step-changes in the voltage during late repolarisation, which was reflected in both the experimental and simulated AP clamp current traces at corresponding time points. Data from [6] (kindly provided by Deo *et al.*) were used to describe homozygous E299V and heterozygous WT-E299V mutant channels. The Nelder-Mead simplex algorithm [61] was used to minimise the least-squared difference between simulated and experimental I-V relationship data for all mutation conditions. Simulated I-V relationships (and AP clamp data [26] for the D172N mutation) are shown in Fig 1, as well as the g_{K1}-V relationship computed from the same data. Details of model equations are given in Supporting S1 Text (S1 Table).

APD restitution

Restitution of the steady-state APD at 90% repolarisation (APD_{90}) was recorded in a single cell model, with multiple conditioning stimuli (of amplitude 20 pA/pF and duration 2.0 ms) employed until a stable solution was reached. A standard S1-S2 protocol was used in order to calculate the restitution of the APD against DI, details of which can be found in Supporting S1 Text. The maximal slope of restitution was calculated as the maximum $\Delta APD/\Delta DI$.

Tissue simulations

Propagation of APs in tissue was described using the monodomain equation [62],

$$\frac{\partial V}{\partial t} = \nabla \cdot \boldsymbol{D}(\nabla V) - \frac{(I_{\mathrm{ion}} + I_{\mathrm{stim}})}{C_{\mathrm{m}}}, \tag{1}$$

where V is the transmembrane voltage, $\boldsymbol{D}$ is the diffusion coefficient tensor, I_{ion} is the total ionic current, I_{stim} is an externally-applied stimulus current used to initiate APs, and C_{m} is the membrane capacitance. Eq (1) was discretised in space using a central differences finite difference method as used in previous modelling studies [23,34,63], and integrated at each time step using a combination of the Rush-Larsen method [64] for gating variables and the forward Euler method for the cellular membrane potential. In tissue simulations, $\boldsymbol{D}$ was set to 0.21 mm^2ms^{-1} with a 9-fold increase along the fibre direction [23,34,63] in order to give values of AT and CV consistent with experimental measurements [28,29].

In multicellular tissue simulations where sustained re-entry was initiated, a power spectrum was obtained through Fourier transform analysis of time series of APs recorded from tissue. The DF was computed using Matlab based on the largest peak in the power spectrum density. This simple approach to computing the DF, which has been described in our previous studies [17,23], is different from the more commonly used definition (e.g. see Ng *et al.* [65]).

Tissue restitution properties

Tissue restitution properties for WT and SQT3 mutant I_{K1} channels were determined using a 1D strand model with spatial step 0.25 mm and length 25 mm. After being paced until a stable solution was reached in a single cell environment, the 1D strand was paced using these initial conditions from one end with five consecutive conditioning S1 stimuli of spatial size 2.5 mm before application of an S2 stimulus and subsequent analysis of the final two APs (to account for APD alternans [66]). Using this protocol, restitution curves for the CV, ERP, and re-entry WL were computed.

The ERP was defined as the minimal S1-S2 coupling required to elicit an AP which propagated at least three-quarters (about 16.25 mm) of the distance down the strand. The CV was determined by dividing the distance of the centre section of the strand (measured at nodes one and three quarters along the strand) by the difference in activation times. WL was computed using WL = CV × ERP. The excitation threshold, a measure of atrial tissue excitability, in each of the WT/mutant cases was determined as described previously [17,40]. Briefly, the 1D strand model was paced using a standard S1-S2 protocol for five beats, following which a stimulus of constant duration but variable amplitude was applied, and the minimum amplitude required to elicit an AP which propagated for a given S2 was recorded as a measure of tissue excitability.

Re-entry in a 2D sheet

In supplementary simulations, spiral wave dynamics were investigated using an idealised 2D sheet of isotropic, electrically-homogeneous tissue with dimensions 100 × 100 mm^2 and spatial step 0.25 mm. A cross-field S1-S2 stimulation protocol was used to initiate re-entrant spiral waves, as in previous modelling studies [17,37]. After being paced until a stable solution was reached in a single cell environment, the 2D tissue patch was then paced using these initial conditions from one edge with four consecutive conditioning S1 stimuli before application of an S2 stimulus of area 50 × 50 mm^2 in the lower left quadrant of the patch. The S2 stimulus was applied at variable times after the ERP, inducing a wave which propagated uni-directionally and subsequently developed into a spiral wave. The cores of spiral waves were tracked

using the method of locating phase singularities [67], and the lifespan and area of meander computed as measures of the stability of re-entry.

Simulations in 3D anatomical human atria geometry

In order to characterise the behaviour of re-entrant excitation waves in an anatomically-realistic setting, the 3D virtual human atrium [34], which is based on the visible human dataset [63], was employed, as in several of our previous modelling studies [23,34,36]. The 3D geometry was taken to be electrically heterogeneous, as it is segmented into distinct regions of the atria, described fully in [23] and shown in Fig 2. The CZ model incorporates a family of regional cell models, accounting for distinct electrophysiological differences in the right atrium (RA), left atrium (LA), right atrial appendage (RAA), left atrial appendage (LAA), crista terminalis (CT), pectinate muscles (PM), atrio-ventricular ring (AVR), atrial septum (AS), Bachmann's bundle (BB), and pulmonary veins (PV). Details are provided in Supporting S1 Text, along with S1B Fig which shows model validation of regional cell models, and S2 Fig which gives a detailed validation of the PV model. In addition, a degree of fibre anisotropy along the bundles of the CT, PM, and BB is included. Re-entry was initiated using the phase distribution method [68], whereby an artificial asymmetric conduction pattern is created, leading to the development of a 3D spiral (scroll) wave (see S9 Fig and Supporting S1 Text for details). Where applicable, averaged DF and lifespan of re-entry were calculated based on time series of APs taken from several locations on the human atria geometry.

Investigating ion channel targets in SQT3-mediated AF

In order to investigate potential pharmaceutical strategies in SQT3-induced AF consequent to abbreviated APD, effects of prolonging APD via modulating variant ion channels on atrial excitation were investigated. This was done by either inhibiting potassium channel currents or augmenting the L-type calcium channel current that prolongs the repolarising phase of the cellular AP. Seeing that SQTS mutations are expressed heterozygously *in vivo*, we only investigated pharmacological modulation of the WT-D172N and WT-E299V mutants–inhibiting potassium channel currents (namely I_{K1}, I_{Kr}, and I_{Kur}) by decreasing their maximal channel conductance (as done in previous studies [24,50,69]) or increasing I_{CaL} (mimicking the effects of a calcium channel agonist [70]). As multichannel agents may show superiority in anti-arrhythmic effects over single channel modulators [71–74], combined single and multiple ion channel blockers were screened, evaluating their actions first on tissue APD dispersion in the 3D virtual human atria, and then on the dynamic behaviours of re-entrant excitation waves initiated by the phase distribution method [68] in the 3D model. In these simulations, the DF and lifespan of re-entry were calculated, where applicable, as indicators of the efficacy of ion channel block combinations.

Comparison with an alternative human atria model

To draw model independent conclusions, comparative simulations were also performed using the GB model of the human atrial AP [33] as used in [6], with simplifications to Ca^{2+} handling described in [75]. The model is mostly derived from human ventricular data [76], and thus exhibits different properties to the CZ model. The GB has a type 3 AP morphology [77], compared to the type 1 AP morphology of the model used in the present study. The aforementioned newly-developed formulations of WT and SQT3 mutant I_{K1} were incorporated into the GB model, and the conductances scaled as in the CZ model. Any additional changes to the baseline model are detailed in Supporting S1 Text.

Supporting information

S1 Text. Detailed information on development of simulation tools and supplementary investigations.
(DOCX)

S1 Fig. Comparison of baseline and regional cell model APs with experimental data. (Ai) A comparison of the baseline right atrium (RA) model AP used in this study with experimental recordings of action potentials taken from human RA myocytes (Bosch *et al*., 1999; Wang *et al*., 1993; Wettwer *et al*., 2004; Koumi *et al*., 1995; Workman *et al*., 2001). (Aii) Validation of baseline model against experimentally-measured metrics; action potential amplitude (APA), maximum upstroke velocity (MUV), action potential duration at 50% and 90% (APD_{50} and APD_{90}, respectively), and resting membrane potential (RMP), using data from Gong *et al*., 2008; Poulet *et al*., 2015; Hordof *et al*., 1976; Gelband *et al*., 1972; Pau *et al*., 2007; Redpath *et al*., 2006; Katoh *et al*., 2005; Bosch *et al*., 1999; Dobrev & Ravens, 2003; Kim *et al*., 2002). (Bi) Regional cell model action potentials from the crista terminalis (CT), right atrial appendage (RAA), atrio-ventricular ring (AVR), atrial septum (AS), Bachmann's bundle (BB), left atrium (LA), pectinate muscles (PM), and RA. (Bii) Comparison of APD_{90} ratios (APD_{95} for Feng *et al*., 1998) in regional cell models using experimental data from Gong *et al*., 2008; Katoh *et al*., 2005; Feng *et al*., 1998; Burashnikov *et al*., 2004; Li *et al*., 2001.
(DOCX)

S2 Fig. Validation of the pulmonary vein model. (A) A comparison of model changes in maximal ionic conductances of I_{to}, I_{Ks}, I_{Kr}, I_{K1}, and I_{CaL} between PV and LA models against experimental measurements. (B) Comparison of AP characteristics, namely APD_{90}, MUV, and APA, between PV and LA models against experimental data. (C) Action potentials from the LA and PV models at a pacing frequency of 2 Hz compared with experimentally-recorded APs from canine atrial myocytes (Cha *et al*., 2005) shown inset. (D) Comparison of difference in RMP between LA and PV models against experimental measurements. All experimental data are taken from Ehrlich *et al*., 2003; Datino *et al*., 2010; Cha *et al*., 2005).
(DOCX)

S3 Fig. Differences in regional cell model tissue APD. ΔAPD at the CT/PM and PV/LA junctions as determined in 1D tissue models.
(DOCX)

S4 Fig. Temporal vulnerability window to uni-directional conduction block. Space-time plots of AP propagation in 1D models of the CT/PM junction used to compute temporal vulnerability to re-entry and corresponding vulnerability window (VW) widths. Three scenarios are shown which correspond to different S2 timings: bi-directional conduction block (A), uni-directional conduction (B), and bi-directional conduction (C). A summary of VW measurements in WT and SQT3 mutation conditions (D).
(DOCX)

S5 Fig. Re-entry simulations in idealised 2D sheet. A summary of rotor trajectories in 2D re-entry simulations for different S2 timings after the effective refractory period (ERP).
(DOCX)

S6 Fig. Spiral wave characteristics in 2D re-entry simulations. (A) Bar charts showing the average lifespan of re-entrant excitations in 5 re-entry simulations corresponding to 5 different S2 timings; and (B) the average area of meander over time.
(DOCX)

S7 Fig. APD dispersion in 3D geometry under simulated pharmacological modulation conditions. (A) WT ΔAPD is shown for reference. ΔAPD in WT-D172N (B) and WT-E299V (C) tissue is shown under the following conditions: (i) control, (ii) 50% I_{Kr} block, (iii) 50% I_{K1} block, (iv) 50% I_{K1}+I_{Kr} block, (v) 50% I_{Kur} block, (vi) 50% I_{Kr}+I_{Kur} block, (vii) 100% increase in I_{CaL}, and (viii) 250% increase in I_{CaL}. The colour bar shows APD relative to the shortest APD measured in each condition, designated APD+. The scale of the colour bar is fixed at the value of ΔAPD in the WT condition (120 ms). Regions where APs failed to exceed −20 mV are shown in black. Single cell AP traces at 1 Hz pacing are shown in WT-D172N (Di, Dii) and WT-E299V (Diii, Div) conditions.
(DOCX)

S8 Fig. Summary of ΔAPD and relative APD prolongation in pharmacological simulation conditions. Floating bar charts showing ΔAPD in WT-D172N (A) and WT-E299V (B) mutant tissue under various simulated drug effect conditions. The WT ΔAPD is shown in light blue for reference.
(DOCX)

S9 Fig. Illustration of the phase distribution method and evolution of scroll waves. (A) Mapping of the membrane potential from an action potential onto the realistic 3D human atria geometry. (B) Evolution of scroll waves in the RA at different time points using phase distribution method initial conditions.
(DOCX)

S10 Fig. AP and I_{K1} profile in WT and mutation conditions using the GB model. Action potential waveforms in WT, WT-D172N, D172N, WT-E299V, and E299V conditions at a pacing frequency of 1 Hz (Ai), with corresponding current trace for I_{K1} (Aii). Restitution of the APD_{90} (Bi), and maximal slope of restitution (Bii).
(DOCX)

S11 Fig. Regional cell models and spatial dispersion of APD in GB model. Regional cell models in the GB model (A), including PM, RAA, CT, BB, AS, LA, LAA, AVR, PV. APD distribution maps in WT (Bi), WT-D172N (Bii), D172N (Biii), WT-E299V (Biv), and E299V (Bv) mutation conditions, with corresponding ΔAPD (C). The colour bar shows APD relative to the shortest APD measured in each condition, designated APD+. The scale of the colour bar is fixed at the value of ΔAPD in the WT condition (72 ms). The colour black shows regions where membrane potentials failed to exceed a threshold value (−20 mV).
(DOCX)

S12 Fig. Comparison of I_{K1} kinetics with previous studies. (A) Simulated I-V relationships for the WT I_{K1} formulation used in the human atrial cell model in the present study compared with the WT I_{K1} formulation used in our previous study in human ventricular cells (Adeniran *et al*., 2012)–the inset shows native I_{K1} recordings in human atrial and ventricular myocytes taken from Wang *et al*., 1998. (B) Simulated I-V relationships for I_{K1} used in this study compared with I_{K1} from the GPB model (Grandi *et al*., 2011), CRN model (Courtemanche *et al*., 1998), and the NFG model (Nygren *et al*., 1998).
(DOCX)

S1 Table. I_{K1} formulation parameters. Parameters of I_{K1} for WT, WT-D172N, D172N, WT-E299V, and E299V mutation conditions, obtained by fitting Equation S1 to experimental data (El Harchi *et al*., 2009; Deo *et al*., 2013). For comparison, formulations of the WT model used in Kharche *et al*., 2008; and the CRN model I_{K1} (Courtemanche *et* al., 1998) are shown.
(DOCX)

S2 Table. Ionic differences in regional cell models. A summary of conductance scaling factors, G_X, for maximal conductance of ionic current I_X relative to the baseline (RA) cell model and corresponding experimental data sources. Abbreviations are as follows: CT = crista terminalis, BB = Bachmann's bundle, PM = pectinate muscles, AVR = atrio-ventricular ring, RAA = right atrial appendage, AS = atrial septum, LA = left atrium, LAA = left atrial appendage, PV = pulmonary veins.
(DOCX)

S3 Table. Dominant frequency in 2D re-entry simulations. A summary of dominant frequencies (DF) in SQT3 mutation conditions in a representative 2D spiral wave re-entry simulation.
(DOCX)

S4 Table. AP properties in WT and SQT3 mutant conditions in the GB model. A summary of AP properties such as action potential amplitude (APA), resting membrane potential (RMP), action potential duration at 50% and 90% repolarisation (APD_{50} and APD_{90}, respectively), and maximum upstroke velocity (MUV) in the GB model in WT and SQT3 mutation conditions at a pacing frequency of 1 Hz.
(DOCX)

S1 Video. WT re-entry in 2D idealised geometry. A representative video of initiation and conduction of spiral waves in a 2D idealised geometry in the WT condition. Re-entry was induced using an S1-S2 protocol: following propagation of a planar wave elicited with four conditioning S1 stimuli at a BCL of 400 ms, an S2 stimulus was applied 40 ms after the effective refractory period in the lower left quadrant of the patch. The initiated spiral wave meanders out of the tissue boundaries in $<$200 ms. The evolution of spiral wave core trajectories (marked by white circles) is superimposed onto the video.
(AVI)

S2 Video. WT-D172N re-entry in 2D idealised geometry. A representative video of initiation and conduction of spiral waves in a 2D idealised geometry in the WT-D172N condition. Re-entry was induced using an S1-S2 protocol: following propagation of a planar wave elicited with four conditioning S1 stimuli at a BCL of 400 ms, an S2 stimulus was applied 40 ms after the effective refractory period in the lower left quadrant of the patch. The initiated spiral wave persists for the duration of the simulation. The evolution of spiral wave core trajectories (marked by white circles) is superimposed onto the video.
(AVI)

S3 Video. D172N re-entry in 2D idealised geometry. A representative video of initiation and conduction of spiral waves in a 2D idealised geometry in the D172N condition. Re-entry was induced using an S1-S2 protocol: following propagation of a planar wave elicited with four conditioning S1 stimuli at a BCL of 400 ms, an S2 stimulus was applied 40 ms after the effective refractory period in the lower left quadrant of the patch. The initiated spiral wave persists for the duration of the simulation. The evolution of spiral wave core trajectories (marked by white circles) is superimposed onto the video.
(AVI)

S4 Video. WT-E299V re-entry in 2D idealised geometry. A representative video of initiation and conduction of spiral waves in a 2D idealised geometry in the WT-E299V condition. Re-entry was induced using an S1-S2 protocol: following propagation of a planar wave elicited with four conditioning S1 stimuli at a BCL of 400 ms, an S2 stimulus was applied 40 ms after

the effective refractory period in the lower left quadrant of the patch. The initiated spiral wave persists for the duration of the simulation. The evolution of spiral wave core trajectories (marked by white circles) is superimposed onto the video.
(AVI)

S5 Video. E299V re-entry in 2D idealised geometry. A representative video of initiation and conduction of spiral waves in a 2D idealised geometry in the E299V condition. Re-entry was induced using an S1-S2 protocol: following propagation of a planar wave elicited with four conditioning S1 stimuli at a BCL of 400 ms, an S2 stimulus was applied 40 ms after the effective refractory period in the lower left quadrant of the patch. The initiated spiral wave persists for >4.0 s before meandering out of the tissue boundary shortly before the end of the simulation. The evolution of spiral wave core trajectories (marked by white circles) is superimposed onto the video.
(AVI)

S6 Video. WT re-entry in 3D anatomical human atria geometry. Re-entrant scroll waves in the WT condition initiated in the 3D human atria shown from two views–looking at the RA posterior wall (left) and into the cavities (right). A single scroll wave persists for ~3.3 s before colliding with its own refractory tail and self-terminating.
(AVI)

S7 Video. WT-D172N re-entry in 3D anatomical human atria geometry. Re-entrant scroll waves in the WT-D172N condition initiated in the 3D human atria shown from two views–looking at the RA posterior wall (left) and into the cavities (right). A single scroll wave follows a stable trajectory in the right atrium along the junction of the crista terminalis and pectinate muscles, persisting for the 5.0 s duration of the simulation.
(AVI)

S8 Video. D172N re-entry in 3D anatomical human atria geometry. Re-entrant scroll waves in the D172N condition initiated in the 3D human atria shown from two views–looking at the RA posterior wall (left) and into the cavities (right). The initial scroll wave quickly degenerates into multiple wavelets, persisting for the 5.0 s duration of the simulation.
(AVI)

S9 Video. WT-E299V re-entry in 3D anatomical human atria geometry. Re-entrant scroll waves in the WT-E299V condition initiated in the 3D human atria shown from two views–looking at the RA posterior wall (left) and into the cavities (right). Scroll waves meander significantly around the right atrial appendage and superior vena caval opening for ~2.0 s, before settling into a stable, anatomical re-entry around the opening of the inferior vena cava which persists for the 5.0 s duration of the simulation.
(AVI)

S10 Video. E299V re-entry in 3D anatomical human atria geometry. Re-entrant scroll waves in the E299V condition initiated in the 3D human atria shown from two views–looking at the RA posterior wall (left) and into the cavities (right). Scroll waves meander significantly around the right atrial appendage and right atrium, before self-terminating at ~2.4 s.
(AVI)

S11 Video. WT-D172N +50% I_{Kr}/I_{Kur} block re-entry in 3D anatomical human atria geometry. Re-entrant scroll waves in the WT-D172N +50% I_{Kr}/I_{Kur} block condition initiated in the 3D human atria shown from two views–looking at the RA posterior wall (left) and into the cavities (right). The initial scroll wave quickly breaks up, de-stabilising the re-entrant circuit and

resulting in self-termination of scroll waves after ~0.5s.
(AVI)

S12 Video. WT-E299V +50% I_{Kr}/I_{Kur} block re-entry in 3D anatomical human atria geometry. Re-entrant scroll waves in the WT-E299V +50% I_{Kr}/I_{Kur} block condition initiated in the 3D human atria shown from two views–looking at the RA posterior wall (left) and into the cavities (right). The initial scroll wave breaks into multiple wavelets which persist for ~4.5 s, before self-terminating.
(AVI)

Acknowledgments

We are grateful to Dr. Michael Colman for useful discussions and assistance. We also thank Dr. Makarand Deo [6] for kindly providing cellular electrophysiology data for the E299V mutation.

Author Contributions

Conceptualization: JCH HZ DGW.

Data curation: DGW HZ JCH AEH.

Formal analysis: DGW HN JCH HZ.

Funding acquisition: JCH HZ.

Investigation: DGW JCH HZ.

Methodology: DGW JCH HZ HN.

Project administration: HZ JCH.

Resources: HZ JCH DGW HN AEH.

Software: DGW HN.

Supervision: HZ JCH.

Validation: DGW.

Visualization: DGW.

Writing – original draft: DGW JCH HZ HN.

Writing – review & editing: DGW JCH HZ HN AEH.

References

1. Dhamoon AS, Jalife J. The inward rectifier current (I_{K1}) controls cardiac excitability and is involved in arrhythmogenesis. Heart Rhythm. 2005; 2: 316–324. https://doi.org/10.1016/j.hrthm.2004.11.012 PMID: 15851327
2. Wang Z, Yue L, White M, Pelletier G, Nattel S. Differential Distribution of Inward Rectifier Potassium Channel Transcripts in Human Atrium Versus Ventricle. Circulation. 1998; 98: 2422–2428. https://doi.org/10.1161/01.CIR.98.22.2422 PMID: 9832487
3. Tristani-Firouzi M, Etheridge SP. Kir 2.1 channelopathies: the Andersen–Tawil syndrome. Pflüg Arch—Eur J Physiol. 2010; 460: 289–294. https://doi.org/10.1007/s00424-010-0820-6 PMID: 20306271
4. Priori SG, Pandit SV, Rivolta I, Berenfeld O, Ronchetti E, Dhamoon A, et al. A Novel Form of Short QT Syndrome (SQT3) Is Caused by a Mutation in the KCNJ2 Gene. Circ Res. 2005; 96: 800–807. https://doi.org/10.1161/01.RES.0000162101.76263.8c PMID: 15761194

5. Hattori T, Makiyama T, Akao M, Ehara E, Ohno S, Iguchi M, et al. A novel gain-of-function KCNJ2 mutation associated with short-QT syndrome impairs inward rectification of Kir2.1 currents. Cardiovasc Res. 2012; 93: 666–673. https://doi.org/10.1093/cvr/cvr329 PMID: 22155372

6. Deo M, Ruan Y, Pandit SV, Shah K, Berenfeld O, Blaufox A, et al. KCNJ2 mutation in short QT syndrome 3 results in atrial fibrillation and ventricular proarrhythmia. Proc Natl Acad Sci. 2013; 110: 4291–4296. https://doi.org/10.1073/pnas.1218154110 PMID: 23440193

7. Xia M, Jin Q, Bendahhou S, He Y, Larroque M- M, Chen Y, et al. A Kir2.1 gain-of-function mutation underlies familial atrial fibrillation. Biochem Biophys Res Commun. 2005; 332: 1012–1019. https://doi.org/10.1016/j.bbrc.2005.05.054 PMID: 15922306

8. Gussak I, Brugada P, Brugada J, Wright RS, Kopecky SL, Chaitman BR, et al. Idiopathic short QT interval: a new clinical syndrome? Cardiology. 2000; 94: 99–102. 47299 https://doi.org/47299 PMID: 11173780

9. Schimpf R, Wolpert C, Gaita F, Giustetto C, Borggrefe M. Short QT syndrome. Cardiovasc Res. 2005; 67: 357–366. https://doi.org/10.1016/j.cardiores.2005.03.026 PMID: 15890322

10. Ambrosini E, Sicca F, Brignone MS, D'Adamo MC, Napolitano C, Servettini I, et al. Genetically induced dysfunctions of Kir2.1 channels: implications for short QT3 syndrome and autism–epilepsy phenotype. Hum Mol Genet. 2014; 23: 4875–4886. https://doi.org/10.1093/hmg/ddu201 PMID: 24794859

11. Bosch RF, Zeng X, Grammer JB, Popovic K, Mewis C, Kühlkamp V. Ionic mechanisms of electrical remodeling in human atrial fibrillation. Cardiovasc Res. 1999; 44: 121–131. https://doi.org/10.1016/S0008-6363(99)00178-9 PMID: 10615396

12. Dobrev D, Wettwer E, Kortner A, Knaut M, Schüler S, Ravens U. Human inward rectifier potassium channels in chronic and postoperative atrial fibrillation. Cardiovasc Res. 2002; 54: 397–404. https://doi.org/10.1016/S0008-6363(01)00555-7 PMID: 12062344

13. Workman AJ, Kane KA, Rankin AC. The contribution of ionic currents to changes in refractoriness of human atrial myocytes associated with chronic atrial fibrillation. Cardiovasc Res. 2001; 52: 226–235. https://doi.org/10.1016/S0008-6363(01)00380-7 PMID: 11684070

14. Wagoner DRV, Pond AL, McCarthy PM, Trimmer JS, Nerbonne JM. Outward K^+ Current Densities and Kv1.5 Expression Are Reduced in Chronic Human Atrial Fibrillation. Circ Res. 1997; 80: 772–781. https://doi.org/10.1161/01.RES.80.6.772 PMID: 9168779

15. Zhang H, Garratt CJ, Zhu J, Holden AV. Role of up-regulation of I_{K1} in action potential shortening associated with atrial fibrillation in humans. Cardiovasc Res. 2005; 66: 493–502. https://doi.org/10.1016/j.cardiores.2005.01.020 PMID: 15914114

16. Pandit SV, Berenfeld O, Anumonwo JMB, Zaritski RM, Kneller J, Nattel S, et al. Ionic Determinants of Functional Reentry in a 2-D Model of Human Atrial Cells During Simulated Chronic Atrial Fibrillation. Biophys J. 2005; 88: 3806–3821. https://doi.org/10.1529/biophysj.105.060459 PMID: 15792974

17. Kharche S, Garratt CJ, Boyett MR, Inada S, Holden AV, Hancox JC, et al. Atrial proarrhythmia due to increased inward rectifier current (I_{K1}) arising from KCNJ2 mutation–A simulation study. Prog Biophys Mol Biol. 2008; 98: 186–197. https://doi.org/10.1016/j.pbiomolbio.2008.10.010 PMID: 19041665

18. Lee Y-S, Hwang M, Song J-S, Li C, Joung B, Sobie EA, et al. The Contribution of Ionic Currents to Rate-Dependent Action Potential Duration and Pattern of Reentry in a Mathematical Model of Human Atrial Fibrillation. PLOS ONE. 2016; 11: e0150779. https://doi.org/10.1371/journal.pone.0150779 PMID: 26964092

19. Feng J, Yue L, Wang Z, Nattel S. Ionic Mechanisms of Regional Action Potential Heterogeneity in the Canine Right Atrium. Circ Res. 1998; 83: 541–551. https://doi.org/10.1161/01.RES.83.5.541 PMID: 9734477

20. Ehrlich JR, Cha T-J, Zhang L, Chartier D, Melnyk P, Hohnloser SH, et al. Cellular electrophysiology of canine pulmonary vein cardiomyocytes: action potential and ionic current properties. J Physiol. 2003; 551: 801–813. https://doi.org/10.1113/jphysiol.2003.046417 PMID: 12847206

21. Cha T-J, Ehrlich JR, Zhang L, Chartier D, Leung TK, Nattel S. Atrial Tachycardia Remodeling of Pulmonary Vein Cardiomyocytes. Circulation. 2005; 111: 728–735. https://doi.org/10.1161/01.CIR.0000155240.05251.D0 PMID: 15699259

22. Gong D, Zhang Y, Cai B, Meng Q, Jiang S, Li X, et al. Characterization and comparison of Na^+, K^+ and Ca^{2+} currents between myocytes from human atrial right appendage and atrial septum. Cell Physiol Biochem Int J Exp Cell Physiol Biochem Pharmacol. 2008; 21: 385–394. https://doi.org/10.1159/000129631 PMID: 18453746

23. Colman MA, Aslanidi OV, Kharche S, Boyett MR, Garratt C, Hancox JC, et al. Pro-arrhythmogenic effects of atrial fibrillation-induced electrical remodelling: insights from the three-dimensional virtual human atria. J Physiol. 2013; 591: 4249–4272. https://doi.org/10.1113/jphysiol.2013.254987 PMID: 23732649

24. Varela M, Colman MA, Hancox JC, Aslanidi OV. Atrial Heterogeneity Generates Re-entrant Substrate during Atrial Fibrillation and Anti-arrhythmic Drug Action: Mechanistic Insights from Canine Atrial Models. PLOS Comput Biol. 2016; 12: e1005245. https://doi.org/10.1371/journal.pcbi.1005245 PMID: 27984585

25. Abrams CJ, Davies NW, Shelton PA, Stanfield PR. The role of a single aspartate residue in ionic selectivity and block of a murine inward rectifier K^+ channel Kir2.1. J Physiol. 1996; 493: 643–649. https://doi.org/10.1113/jphysiol.1996.sp021411 PMID: 8799888

26. El Harchi A, McPate MJ, Zhang Y hong, Zhang H, Hancox JC. Action potential clamp and chloroquine sensitivity of mutant Kir2.1 channels responsible for variant 3 short QT syndrome. J Mol Cell Cardiol. 2009; 47: 743–747. https://doi.org/10.1016/j.yjmcc.2009.02.027 PMID: 19285083

27. Anumonwo JMB, Lopatin AN. Cardiac strong inward rectifier potassium channels. J Mol Cell Cardiol. 2010; 48: 45–54. https://doi.org/10.1016/j.yjmcc.2009.08.013 PMID: 19703462

28. Lemery R, Birnie D, Tang AS l., Green M, Gollob M, Hendry M, et al. Normal Atrial Activation and Voltage During Sinus Rhythm in the Human Heart: An Endocardial and Epicardial Mapping Study in Patients with a History of Atrial Fibrillation. J Cardiovasc Electrophysiol. 2007; 18: 402–408. https://doi.org/10.1111/j.1540-8167.2007.00762.x PMID: 17394455

29. Fedorov VV, Glukhov AV, Chang R, Kostecki G, Aferol H, Hucker WJ, et al. Optical mapping of the isolated coronary-perfused human sinus node. J Am Coll Cardiol. 2010; 56: 1386–1394. https://doi.org/10.1016/j.jacc.2010.03.098 PMID: 20946995

30. Nygren A, Fiset C, Firek L, Clark JW, Lindblad DS, Clark RB, et al. Mathematical Model of an Adult Human Atrial Cell The Role of K^+ Currents in Repolarization. Circ Res. 1998; 82: 63–81. https://doi.org/10.1161/01.RES.82.1.63 PMID: 9440706

31. Burashnikov A, Mannava S, Antzelevitch C. Transmembrane action potential heterogeneity in the canine isolated arterially perfused right atrium: effect of IKr and IKur/Ito block. Am J Physiol—Heart Circ Physiol. 2004; 286: H2393–H2400. https://doi.org/10.1152/ajpheart.01242.2003 PMID: 15148061

32. Fenton FH, Cherry EM, Hastings HM, Evans SJ. Multiple mechanisms of spiral wave breakup in a model of cardiac electrical activity. Chaos Interdiscip J Nonlinear Sci. 2002; 12: 852–892. https://doi.org/10.1063/1.1504242 PMID: 12779613

33. Grandi E, Pandit SV, Voigt N, Workman AJ, Dobrev D, Jalife J, et al. Human Atrial Action Potential and Ca^{2+} Model: Sinus Rhythm and Chronic Atrial Fibrillation. Circ Res. 2011; 109: 1055–1066. https://doi.org/10.1161/CIRCRESAHA.111.253955 PMID: 21921263

34. Aslanidi OV, Colman MA, Stott J, Dobrzynski H, Boyett MR, Holden AV, et al. 3D virtual human atria: A computational platform for studying clinical atrial fibrillation. Prog Biophys Mol Biol. 2011; 107: 156–168. https://doi.org/10.1016/j.pbiomolbio.2011.06.011 PMID: 21762716

35. Aslanidi OV, Al-Owais M, Benson AP, Colman M, Garratt CJ, Gilbert SH, et al. Virtual tissue engineering of the human atrium: Modelling pharmacological actions on atrial arrhythmogenesis. Eur J Pharm Sci. 2012; 46: 209–221. https://doi.org/10.1016/j.ejps.2011.08.014 PMID: 21888968

36. Kharche S, Adeniran I, Stott J, Law P, Boyett MR, Hancox JC, et al. Pro-arrhythmogenic effects of the S140G KCNQ1 mutation in human atrial fibrillation–insights from modelling. J Physiol. 2012; 590: 4501–4514. https://doi.org/10.1113/jphysiol.2012.229146 PMID: 22508963

37. Loewe A, Wilhelms M, Fischer F, Scholz EP, Dössel O, Seemann G. Arrhythmic potency of human ether-à-go-go-related gene mutations L532P and N588K in a computational model of human atrial myocytes. Europace. 2014; 16: 435–443. https://doi.org/10.1093/europace/eut375 PMID: 24569898

38. Whittaker DG, Colman MA, Ni H, Hancox JC, Zhang H. In silico investigation of short QT syndrome-linked potassium channel mutations on electro-mechanical function of human atrial cells. Computing in Cardiology Conference (CinC), 2015. 2015. pp. 853–856. https://doi.org/10.1109/CIC.2015.7411045

39. Delacrétaz E. Supraventricular Tachycardia. N Engl J Med. 2006; 354: 1039–1051. https://doi.org/10.1056/NEJMcp051145 PMID: 16525141

40. Adeniran I, Harchi AE, Hancox JC, Zhang H. Proarrhythmia in KCNJ2-linked short QT syndrome: insights from modelling. Cardiovasc Res. 2012; 94: 66–76. https://doi.org/10.1093/cvr/cvs082 PMID: 22308236

41. Meijer van Putten RME, Mengarelli I, Guan K, Zegers JG, van Ginneken ACG, Verkerk AO, et al. Ion channelopathies in human induced pluripotent stem cell derived cardiomyocytes: a dynamic clamp study with virtual I_{K1}. Front Physiol. 2015; 6. https://doi.org/10.3389/fphys.2015.00007 PMID: 25691870

42. Allessie MA, Bonke FI, Schopman FJ. Circus movement in rabbit atrial muscle as a mechanism of tachycardia. II. The role of nonuniform recovery of excitability in the occurrence of unidirectional block, as studied with multiple microelectrodes. Circ Res. 1976; 39: 168–177. https://doi.org/10.1161/01.RES.39.2.168 PMID: 939001

43. Spach MS, Dolber PC, Heidlage JF. Interaction of inhomogeneities of repolarization with anisotropic propagation in dog atria. A mechanism for both preventing and initiating reentry. Circ Res. 1989; 65: 1612–1631. https://doi.org/10.1161/01.RES.65.6.1612 PMID: 2582593
44. Allessie M, Ausma J, Schotten U. Electrical, contractile and structural remodeling during atrial fibrillation. Cardiovasc Res. 2002; 54: 230–246. https://doi.org/10.1016/S0008-6363(02)00258-4 PMID: 12062329
45. Koumi S, Backer CL, Arentzen CE. Characterization of Inwardly Rectifying K^+ Channel in Human Cardiac Myocytes. Circulation. 1995; 92: 164–174. https://doi.org/10.1161/01.CIR.92.2.164 PMID: 7600647
46. Rodríguez-Menchaca AA, Navarro-Polanco RA, Ferrer-Villada T, Rupp J, Sachse FB, Tristani-Firouzi M, et al. The molecular basis of chloroquine block of the inward rectifier Kir2.1 channel. Proc Natl Acad Sci. 2008; 105: 1364–1368. https://doi.org/10.1073/pnas.0708153105 PMID: 18216262
47. Lopez-Izquierdo A, Ponce-Balbuena D, Ferrer T, Sachse FB, Tristani-Firouzi M, Sánchez-Chapula JA. Chloroquine Blocks a Mutant Kir2.1 Channel Responsible for Short QT Syndrome and Normalizes Repolarization Properties in silico. Cell Physiol Biochem. 2009; 24: 153–160. https://doi.org/10.1159/000233241 PMID: 19710529
48. Tobón C, Duarte M, Duque JE, Becerra MA, Arango SS, Cardona K, et al. Chloroquine effect on rotor termination under paroxysmal and chronic atrial fibrillation. 2D simulation study. 2014 IEEE Central America and Panama Convention (CONCAPAN XXXIV). 2014. pp. 1–5. 10.1109/CONCAPAN.2014.7000401
49. Noujaim SF, Stuckey JA, Ponce-Balbuena D, Ferrer-Villada T, López-Izquierdo A, Pandit SV, et al. Structural bases for the different anti-fibrillatory effects of chloroquine and quinidine. Cardiovasc Res. 2011; 89: 862–869. https://doi.org/10.1093/cvr/cvr008 PMID: 21233253
50. Mirams GR, Cui Y, Sher A, Fink M, Cooper J, Heath BM, et al. Simulation of multiple ion channel block provides improved early prediction of compounds' clinical torsadogenic risk. Cardiovasc Res. 2011; 91: 53–61. https://doi.org/10.1093/cvr/cvr044 PMID: 21300721
51. Courtemanche M, Ramirez RJ, Nattel S. Ionic targets for drug therapy and atrial fibrillation-induced electrical remodeling: insights from a mathematical model. Cardiovasc Res. 1999; 42: 477–489. https://doi.org/10.1016/S0008-6363(99)00034-6 PMID: 10533583
52. Morgan R, Colman MA, Chubb H, Seemann G, Aslanidi OV. Slow Conduction in the Border Zones of Patchy Fibrosis Stabilizes the Drivers for Atrial Fibrillation: Insights from Multi-Scale Human Atrial Modeling. Front Physiol. 2016; 7. https://doi.org/10.3389/fphys.2016.00474 PMID: 27826248
53. Schimpf R, Antzelevitch C, Haghi D, Giustetto C, Pizzuti A, Gaita F, et al. Electromechanical coupling in patients with the short QT syndrome: Further insights into the mechanoelectrical hypothesis of the U wave. Heart Rhythm. 2008; 5: 241–245. https://doi.org/10.1016/j.hrthm.2007.10.015 PMID: 18242547
54. Adeniran I, Hancox JC, Zhang H. In silico investigation of the short QT syndrome, using human ventricle models incorporating electromechanical coupling. Front Physiol. 2013; 4. https://doi.org/10.3389/fphys.2013.00166 PMID: 23847545
55. Frea S, Giustetto C, Capriolo M, Scrocco C, Fornengo C, Benedetto S, et al. New echocardiographic insights in short QT syndrome: More than a channelopathy? Heart Rhythm. 2015; 12: 2096–2105. https://doi.org/10.1016/j.hrthm.2015.05.024 PMID: 26001507
56. Nash MP, Panfilov AV. Electromechanical model of excitable tissue to study reentrant cardiac arrhythmias. Prog Biophys Mol Biol. 2004; 85: 501–522. https://doi.org/10.1016/j.pbiomolbio.2004.01.016 PMID: 15142759
57. Keldermann RH, Nash MP, Gelderblom H, Wang VY, Panfilov AV. Electromechanical wavebreak in a model of the human left ventricle. Am J Physiol—Heart Circ Physiol. 2010; 299: H134–H143. https://doi.org/10.1152/ajpheart.00862.2009 PMID: 20400690
58. Courtemanche M, Ramirez RJ, Nattel S. Ionic mechanisms underlying human atrial action potential properties: insights from a mathematical model. Am J Physiol—Heart Circ Physiol. 1998; 275: H301–H321.
59. Dhamoon AS, Pandit SV, Sarmast F, Parisian KR, Guha P, Li Y, et al. Unique Kir2.x Properties Determine Regional and Species Differences in the Cardiac Inward Rectifier K^+ Current. Circ Res. 2004; 94: 1332–1339. https://doi.org/10.1161/01.RES.0000128408.66946.67 PMID: 15087421
60. Calvo CJ, Deo M, Zlochiver S, Millet J, Berenfeld O. Attraction of Rotors to the Pulmonary Veins in Paroxysmal Atrial Fibrillation: A Modeling Study. Biophys J. 2014; 106: 1811–1821. https://doi.org/10.1016/j.bpj.2014.02.030 PMID: 24739180
61. Nelder JA, Mead R. A Simplex Method for Function Minimization. Comput J. 1965; 7: 308–313. https://doi.org/10.1093/comjnl/7.4.308

62. Clayton RH, Bernus O, Cherry EM, Dierckx H, Fenton FH, Mirabella L, et al. Models of cardiac tissue electrophysiology: Progress, challenges and open questions. Prog Biophys Mol Biol. 2011; 104: 22–48. https://doi.org/10.1016/j.pbiomolbio.2010.05.008 PMID: 20553746

63. Seemann G, Höper C, Sachse FB, Dössel O, Holden AV, Zhang H. Heterogeneous three-dimensional anatomical and electrophysiological model of human atria. Philos Trans R Soc Lond Math Phys Eng Sci. 2006; 364: 1465–1481. https://doi.org/10.1098/rsta.2006.1781 PMID: 16766355

64. Rush S, Larsen H. A Practical Algorithm for Solving Dynamic Membrane Equations. IEEE Trans Biomed Eng. 1978; BME-25: 389–392. https://doi.org/10.1109/TBME.1978.326270 PMID: 689699

65. Ng J, Kadish AH, Goldberger JJ. Effect of electrogram characteristics on the relationship of dominant frequency to atrial activation rate in atrial fibrillation. Heart Rhythm. 2006; 3: 1295–1305. https://doi.org/10.1016/j.hrthm.2006.07.027 PMID: 17074635

66. Wilhelms M, Hettmann H, Maleckar MM, Koivumäki JT, Dössel O, Seemann G. Benchmarking electrophysiological models of human atrial myocytes. Front Physiol. 2013; 3. https://doi.org/10.3389/fphys.2012.00487 PMID: 23316167

67. Bray M- A, Wikswo JP. Use of topological charge to determine filament location and dynamics in a numerical model of scroll wave activity. IEEE Trans Biomed Eng. 2002; 49: 1086–1093. https://doi.org/10.1109/TBME.2002.803516 PMID: 12374332

68. Biktashev VN, Holden AV. Reentrant waves and their elimination in a model of mammalian ventricular tissue. Chaos Interdiscip J Nonlinear Sci. 1998; 8: 48–56. https://doi.org/10.1063/1.166307 PMID: 12779709

69. Colman MA, Varela M, Hancox JC, Zhang H, Aslanidi OV. Evolution and pharmacological modulation of the arrhythmogenic wave dynamics in canine pulmonary vein model. Europace. 2014; 16: 416–423. https://doi.org/10.1093/europace/eut349 PMID: 24569896

70. Bechem M, Schramm M. Calcium-Agonists. J Mol Cell Cardiol. 1987; 19, Supplement 2: 63–75. https://doi.org/10.1016/S0022-2828(87)80005-6

71. Sicouri S, Burashnikov A, Belardinelli L, Antzelevitch C. Synergistic Electrophysiologic and Antiarrhythmic Effects of the Combination of Ranolazine and Chronic Amiodarone in Canine AtriaCLINICAL PERSPECTIVE. Circ Arrhythm Electrophysiol. 2010; 3: 88–95. https://doi.org/10.1161/CIRCEP.109.886275 PMID: 19952329

72. Burashnikov A, Sicouri S, Di Diego JM, Belardinelli L, Antzelevitch C. Synergistic Effect of the Combination of Ranolazine and Dronedarone to Suppress Atrial Fibrillation. J Am Coll Cardiol. 2010; 56: 1216–1224. https://doi.org/10.1016/j.jacc.2010.08.600 PMID: 20883928

73. Reiffel JA, Camm AJ, Belardinelli L, Zeng D, Karwatowska-Prokopczuk E, Olmsted A, et al. The HARMONY Trial. Circ Arrhythm Electrophysiol. 2015; 8: 1048–1056. https://doi.org/10.1161/CIRCEP.115.002856 PMID: 26226999

74. Aguilar M, Xiong F, Qi XY, Comtois P, Nattel S. Potassium Channel Blockade Enhances Atrial Fibrillation–Selective Antiarrhythmic Effects of Optimized State-Dependent Sodium Channel Blockade. Circulation. 2015; 132: 2203–2211. https://doi.org/10.1161/CIRCULATIONAHA.115.018016 PMID: 26499964

75. Chang KC, Bayer JD, Trayanova NA. Disrupted Calcium Release as a Mechanism for Atrial Alternans Associated with Human Atrial Fibrillation. PLOS Comput Biol. 2014; 10: e1004011. https://doi.org/10.1371/journal.pcbi.1004011 PMID: 25501557

76. Grandi E, Pasqualini FS, Bers DM. A novel computational model of the human ventricular action potential and Ca transient. J Mol Cell Cardiol. 2010; 48: 112–121. https://doi.org/10.1016/j.yjmcc.2009.09.019 PMID: 19835882

77. Wang Z, Fermini B, Nattel S. Delayed rectifier outward current and repolarization in human atrial myocytes. Circ Res. 1993; 73: 276–285. https://doi.org/10.1161/01.RES.73.2.276 PMID: 8330373
